# National trends in adolescents’ mental health by income level in South Korea, pre– and post–COVID–19, 2006–2022

**DOI:** 10.1038/s41598-024-74073-5

**Published:** 2024-10-23

**Authors:** Jaehyeong Cho, Jaeyu Park, Hayeon Lee, Hyesu Jo, Sooji Lee, Hyeon Jin Kim, Yejun Son, Hyunjee Kim, Selin Woo, Seokjun Kim, Jiseung Kang, Damiano Pizzol, Jiyoung Hwang, Lee Smith, Dong Keon Yon

**Affiliations:** 1 Department of Medicine, CHA University School of Medicine, Seongnam, South Korea; 2 Center for Digital Health, Medical Science Research Institute, Kyung Hee University College of Medicine, 23 Kyungheedae-ro, Dongdaemun-gu, Seoul, 02447 South Korea; 3 Department of Regulatory Science, Kyung Hee University, Seoul, South Korea; 4 Department of Medicine, Kyung Hee University College of Medicine, Seoul, South Korea; 5 Department of Precision Medicine, Kyung Hee University College of Medicine, Seoul, South Korea; 6 Division of Sleep Medicine, Harvard Medical School, Boston, MA USA; 7 Department of Anesthesia, Critical Care and Pain Medicine, Massachusetts General Hospital, Boston, MA USA; 8 Health Unit Eni, Maputo, Mozambique; 9 Health Unit, Eni, San Donato Milanese, Italy; 10 Centre for Health, Performance and Wellbeing, Anglia Ruskin University, East Rd, Cambridge, CB1 1PT UK; 11 Department of Biomedical Engineering, Kyung Hee University, Yongin, South Korea; 12 Department of Pediatrics, Kyung Hee University College of Medicine, 23 Kyungheedae-ro, Dongdaemun-gu, Seoul, 02447 South Korea

**Keywords:** Adolescents, COVID–19, Household income, Mental health, Sadness, Stress, Suicide, Public health, Epidemiology

## Abstract

Despite the significant impact of the COVID–19 pandemic on various factors related to adolescent mental health problems such as stress, sadness, suicidal ideation, and suicide attempts, research on this topic has been insufficient to date. This study is based on the Korean Youth Risk Behavior Web–based Survey from 2006 to 2022. We analyzed the mental health problems of adolescents based on questionnaires with medical interviews, within five income groups and compared them with several risk factors. A total of 1,138,804 participants were included in this study, with a mean age (SD) of 15.01 (0.75) years. Of these, 587,256 were male (51.57%). In 2022, the recent period from the study, the weighted prevalence of stress in highest income group was 40.07% (95% CI, 38.67–41.48), sadness was 28.15% (26.82–29.48), suicidal ideation was 13.92% (12.87–14.97), and suicide attempts was 3.42% (2.90–3.93) while the weighted prevalence of stress in lowest income group was 62.77% (59.42–66.13), sadness was 46.83% (43.32–50.34), suicidal ideation was 31.70% (28.44–34.96), and suicide attempts was 10.45% (8.46–12.45). Lower income groups showed a higher proportion with several risk factors. Overall proportion had decreased until the onset of the pandemic. However, a significant increase has been found during the COVID–19 pandemic. Our study showed an association between household income level and the prevalence of mental illness in adolescents. Furthermore, the COVID–19 pandemic has exacerbated mental illness among adolescents from low household income level, underscoring the necessity for heightened public attention and measures targeted at this demographic.

**Supplementary Information:**

The online version contains supplementary material available at 10.1038/s41598-024-74073-5.

## Introduction

In recent years, South Korea has been identified as having the highest rates of adolescent suicide within the Organization for Economic Co-operation and Development, leading to a significant surge in interest regarding mental health problems among adolescents^1^. Cultural shift due to exposure to diverse social media, the COVID–19 pandemic, and rising individualism have drawn public attention to mental health problems of adolescents^2^. Given this perspective, deeper investigations are needed to identify the factors negatively affecting mental health of adolescents. Most studies have primarily focused on adolescents themselves by investigating academic stress or their own mental problems, but there is a need for deeper investigations into socioeconomic factors^1,3–5^. Therefore, we aimed to focus on household income inequality, an essential factor that impacts adolescents to manage their own lives^3^.

Since the onset of the COVID–19 pandemic and the subsequent lockdowns, individuals have experienced increased stress and anxiety due to fears of infection and disruptions to daily routines^6^. Additionally, many have faced social isolation, loneliness, and reduced access to therapeutic services, leading to declines in both physical and mental health^7^. These negative effects have been especially pronounced among adolescents, who have shown a significant rise in negative mental health indicators^8,9^.

This shift highlights the necessity to examine the influence of external factors, especially household income level, on the psychological well-being of adolescents during these unprecedented times^10^. Understanding the association between these factors and adolescent mental health and how this relationship has changed since the pandemic is of paramount importance. Identifying these correlations is crucial for creating more equitable support systems that address the unique challenges faced by vulnerable populations during crises like the COVID–19 pandemic^4,5,11,12^.

Adolescence represents a critical period for personal development, marked by the formation of distinct personalities^13^. The mental health status during this formative period not only shapes an individual’s core values but also has a lasting impact on their life trajectory. Consequently, ensuring the maintenance of adolescent mental well-being throughout these crucial years is imperative for fostering a healthy transition into adulthood^14^.

The process of identity formation during adolescence is significantly influenced by interactions with the surrounding environment such as family and peers^15^. However, due to global social distancing policies, including school closures and home-based education during the COVID–19 pandemic, as seen in South Korea, social interactions among adolescents have been significantly reduced, leading to a negative impact on their mental health^16,17^. This necessitates a comprehensive view of surrounding circumstances in research. Consequently, our study aimed to discover how household income level can influence the mental health of adolescents in South Korea before and during the COVID–19 pandemic.

## Methods

### Study design

This study is based on the Korea Youth Risk Behavior Web-based Survey (KYRBS). This web-based survey was annually conducted by Korea Disease Control and Prevention Agency and reviewed by the Korean Ministry of Education and Ministry of Health and Welfare^18^. Total of 1,138,804 youth enrolled in middle and high schools (aged 12 to 18) participated in the survey from 2006 to 2022. All of the students voluntarily participated in the survey (mean response rate, 95%).^19^ The sampling methodology was executed in a structured three-phase process. First, it delineated the population based on geographical and educational parameters. Second, methods of proportional allocation were employed to distribute the samples. The final step employed stratified cluster sampling through the utilization of schools and classrooms as the primary and secondary sampling units, respectively. This approach significantly reduced the potential for sampling discrepancies. Furthermore, incorporating weighting adjustments facilitated a comprehensive representation of the Korean adolescent demographics.

KYRBS data were anonymous, and the study protocol was approved by the Institutional Review Board of the Korean Disease Control and Prevention Agency (2014-06EXP-02-P-A) and by the local law of the Population Health Promotion Act 19 (117058) from the Korean government. All the participants (or their parents or legal guardians in the case of children under 16) provided written informed consent. This study was conducted in accordance with the principles of the Declaration of Helsinki.

### Covariates

Household income level was classified as five levels by categorizing the subjective perception of the survey participants, as captured by the question, ‘Please indicate your household’s socioeconomic position’. Other variables included grade (7–9th [middle school] and 10–12th [high school]), sex, residential area (rural and urban), recent alcohol consumption (yes and no), smoking status (yes and no), parental educational attainment (high school diploma or less, bachelor’s degree or higher), school performance (high, middle, and low). Following regions are included in rural (Chungbuk, Chungnam, Gangwon, Gyeongbuk, Gyeongnam, Jeonbuk, Jeonnam, and Jeju) and others are included in urban (Busan, Daegu, Daejeon, Gwangju, Gyeonggi, Incheon, Seoul, Sejong, and Ulsan)^20^. Recent alcohol intake was categorized as consuming alcohol at least once in the past month. Concurrently, the definition of smoking status encompassed individuals who had smoked any form of cigarette product at least once over the past month^21^. Additionally, the highest educational attainment of the parents was identified as the highest level of education achieved by either parent.

### End points

We investigated how adolescents’ mental health indicators have changed and assessed whether the COVID–19 pandemic has had an impact on these trends. Since the first confirmed case of COVID–19 in South Korea was reported in January 2020, we divided the entire timeline into eight periods. The pre-pandemic era was divided into five periods: period 1 (2006–2008), period 2 (2009–2011), period 3 (2012–2014), period 4 (2015–2017), and period 5 (2018–2019).

In 2020, the country experienced its first major outbreak, leading to strict social distancing measures, including school closures and remote learning^22,23^. In 2021, despite vaccination efforts, South Korea faced larger waves of infections and many preventive measures remained while some restrictions were eased. In 2022, with the emergence of the Omicron variant, South Korea experienced its highest-ever COVID–19 prevalence rate. Despite this, the mortality rate was 0.13%, the lowest among the 30 countries with the highest case counts^24^. However, South Korea also transitioned to a “living with COVID–19” strategy, gradually lifting most restrictions. Thus, the pandemic era was also divided into three periods: period 6 (2020), period 7 (2021), and period 8 (2022)^21^.

The main purpose of our study is to investigate perceived stress level, sadness, suicidal ideation, and suicide attempts in relation to the household income level^1,9^. To evaluate stress levels, participants were asked to respond to the following question: “How much stress do you typically experience?”. Responses were scored from 1 to 5, and we recategorized scores as low, middle, and high. In the case of sadness, suicidal ideation, and suicide attempts, respondents were categorized into two groups: yes or no. To assess this, questions were asked respectively: “Have you experienced sadness or despair in the past 12 months that was severe enough to interrupt your daily activities for a continuous period of two weeks?“, “Have you seriously considered suicide in the past 12 months?”, and “Have you attempted suicide in the past 12 months?”^2,17,19,25^. Household income level is divided into five categories: high, middle-high, middle, middle-low, low. Furthermore, we initially compared the trends of these indicators based on household income level and conducted more detailed examinations based on sex, grade, residential area, recent alcohol consumption, smoking status, parents’ highest educational level, and school performance before and during the COVID–19 pandemic.

### Statistical analysis

We analyzed the KYRBS data to investigate the national trends in stress, sadness, suicidal ideation, and suicide attempts during the past 17 years. All missing data in the ‘age’ covariate were imputed using the standardized mean value of the same year. Regression analysis was used to check the importance of changes over time and patterns. The findings were shared as weighted odds ratios (wORs), or as weighted β-coefficients with a 95% confidence interval (CI). Binary logistic regression and beta difference (β_diff_) were employed to compare 2019 to 2020, 2020 to 2021, and 2021 to 2022^20^. Moreover, the β_diff_ was calculated to evaluate the change in prevalence of depression and suicide attempts before and during the pandemic. We compared the shift in risk factors in the years following the onset of the COVID–19 pandemic (2019–2022) using Fisher’s exact test for types of data that fit into categories. We described the comparison among the household income levels to determine whether the difference was statistically significant, utilizing the odds ratio with a 95% confidence interval^26^. For all our data analysis, we used SAS software (version 9.4; Institute Inc., Cary, NC, USA). We considered results statistically significant if the two-sided p-value < 0.05.^27^

## Results

Between 2006 and 2022, the KYRBS included a total of 1,138,804 adolescents, comprising 587,256 males (51.57%) and 551,548 females (48.43%). The participants had a mean age [SD] of 15.01 [1.75] years. Among them, 583,626 (51.25%) were middle school students, and 555,178 (48.75%) were high school students. The crude and weighted baseline characteristics of the participants are detailed in Table1 and Table S1.

**Table 1:** Baseline characteristics of adolescents from the KYRBS, 2006–2022.

Variables		Total	Before pandemic					During pandemic		
			2006–2008	2009–2011	2012–2014	2015–2017	2018–2019	2020	2021	2022
Overall, n (%)		1,138,804 (100.00)	221,340 (19.44)	223,947 (19.67)	218,681 (19.20)	195,847 (17.20)	117,343 (10.30)	54,948 (4.83)	54,848 (4.82)	51,850 (4.56)
Age, mean (SD)		15.01 (1.75)	15.00 (1.74)	15.07 (1.74)	14.91 (1.75)	14.98 (1.74)	14.99 (1.77)	15.10 (1.75)	15.09 (1.74)	15.10 (1.73)
Sex, n (%)	Male	587,256 (51.57)	115,948 (52.38)	115,876 (51.74)	111,346 (50.92)	100,631 (51.38)	60,304 (51.39)	28,353 (51.60)	28,401 (51.78)	26,397 (50.91)
	Female	551,548 (48.43)	105,392 (47.62)	108,071 (48.26)	107,335 (49.08)	95,216 (48.62)	57,039 (48.61)	26,595 (48.40)	26,447 (48.22)	25,453 (49.09)
Grade level, n (%)	Middle school (7–9th )	583,626 (51.25)	115,183 (52.04)	114,453 (51.11)	109,983 (50.29)	97,403 (49.73)	59,613 (50.80)	28,961 (52.71)	30,015 (54.72)	28,015 (54.03)
	High school (10–12th )	555,178 (48.75)	106,157 (47.96)	109,494 (48.89)	108,698 (49.71)	98,444 (50.27)	57,730 (49.20)	25,987 (47.29)	24,833 (45.28)	23,835 (45.97)
Residential area, n (%)	Rural	413,743 (36.33)	89,425 (40.40)	86,232 (38.51)	74,446 (34.04)	67,326 (34.38)	40,196 (34.26)	19,091 (34.74)	19,040 (34.71)	17,987 (34.69)
	Urban	725,061 (63.67)	131,915 (59.60)	137,715 (61.49)	144,235 (65.96)	128,521 (65.62)	77,147 (65.74)	35,857 (65.26)	35,808 (65.29)	33,863 (65.31)
Recent alcohol consumption, n (%)	No	927,046 (81.41)	161,104 (72.79)	176,814 (78.95)	180,780 (82.67)	165,800 (84.66)	99,276 (84.60)	49,056 (89.28)	49,045 (89.42)	45,171 (87.12)
	Yes	211,758 (18.59)	60,236 (27.21)	47,133 (21.05)	37,901 (17.33)	30,047 (15.34)	18,067 (15.40)	5,892 (10.72)	5,803 (10.58)	6,679 (12.88)
Smoking status, n (%)	No	1,032,955 (90.71)	192,639 (87.03)	195,480 (87.29)	194,975 (89.16)	181,815 (92.84)	111,209 (94.77)	53,727 (97.78)	53,194 (96.98)	49,916 (96.27)
	Yes	105,849 (9.29)	28,701 (12.97)	28,467 (12.71)	23,706 (10.84)	14,032 (7.16)	6,134 (5.23)	1,221 (2.22)	1,654 (3.02)	1,934 (3.73)
Parental educational attainment, n (%)	High school diploma or less	577,814 (48.35)	132,914 (56.45)	119,797 (50.44)	105,737 (46.57)	83,309 (40.82)	56,773 (47.16)	27,947 (49.45)	27,518 (48.84)	23,819 (44.41)
	Bachelor’s degree or higher	560,990 (51.65)	88,426 (43.55)	104,150 (49.56)	112,944 (53.43)	112,538 (59.18)	60,570 (52.84)	27,001 (50.55)	27,330 (51.16)	28,031 (55.59)
Academic achievement, n (%)	Low	418,440 (37.00)	81,147 (37.05)	78,512 (35.25)	77,240 (35.37)	75,453 (38.28)	45,363 (38.42)	20,146 (36.86)	20,528 (37.14)	20,051 (38.80)
	Middle	319,483 (28.59)	59,952 (26.86)	60,245 (26.94)	60,164 (27.61)	55,388 (28.41)	34,760 (29.74)	16,585 (30.13)	16,903 (31.01)	15,486 (30.02)
	High	400,881 (34.41)	80,241 (36.09)	85,190 (37.81)	81,277 (37.02)	65,006 (33.31)	37,220 (31.84)	18,217 (33.01)	17,417 (31.85)	16,313 (31.18)

Abbreviations: SD, Standard deviation; KYRBS, the Korea Youth Risk Behavior Survey.

The overall and weighted prevalence of perceived stress levels, sadness, suicidal ideation, and suicide attempt is presented in Table1, Table S2, and Table S3. In the most recent survey conducted in 2022, the prevalence of stress levels among participants from high, middle-high, middle, middle-low, and low-income groups were 40.07% (95% CI, 38.67 to 41.48), 38.82% (37.91 to 39.73), 40.41% (39.61 to 41.21), 52.10% (50.47 to 53.73), and 62.77% (59.42 to 66.13), respectively. Similar patterns were observed for sadness [28.15% (26.82 to 29.48), 27.13% (26.32 to 27.94), 27.49% (26.80 to 28.18), 37.64% (36.07 to 39.20), 46.83% (43.32 to 50.34)], suicidal ideation [13.92% (12.87 to 14.97), 12.58% (11.94 to 13.22), 13.27% (12.76 to 13.78), 22.25% (20.87 to 23.64), 31.70% (28.44 to 34.96)], and suicide attempt [3.42% (2.90 to 3.93), 2.14% (1.87 to 2.41), 2.14% (1.87 to 2.41), 3.79% (3.18 to 4.41), 10.45% (8.46 to 12.45)]. These results suggest that adolescents from lower-income households are more likely to experience elevated stress, sadness, and suicidal behavior. However, uniquely in the case of suicide attempts, the highest income group showed a higher value in comparison with the middle and middle-high income groups (Table 2).

**Table 2:** National trends and weighted prevalence of adolescents’ mental health indicators by household income level, KYRBS, 2006–2022, weighted % (95% CI).

Variables			Pre-pandemic					Pandemic			Trend in the pre-pandemic era, β (95% CI)	Trend in the pandemic era, β (95% CI)	Trend difference, β_diff_ (95% CI)
			2006–2008	2009–2011	2012–2014	2015–2017	2018–2019	2020	2021	2022			
**Perceived stress level**													
Overall		High	38.93 (37.92–39.95)	38.09 (37.04–39.15)	33.93 (33.09–34.77)	31.96 (31.23–32.69)	34.81 (33.84–35.79)	28.30 (26.83–29.76)	36.63 (35.15–38.10)	40.07 (38.67–41.48)	**-1.41** **(-1.72 to –1.10)**	**2.39 (1.84 to 2.94) **	**3.80 (3.17 to 4.43) **
		Mid-high	40.92 (40.33–41.51)	37.68 (37.11–38.24)	35.11 (34.61–35.61)	33.40 (32.90-33.91)	37.27 (36.56–37.98)	31.75 (30.85–32.65)	36.65 (35.72–37.59)	38.82 (37.91–39.73)	**-1.21** **(-1.41﻿ to –1.01)**	**0.93 (0.56 to 1.30)**	**2.14 (1.72 to 2.56) **
		Middle	43.50 (43.02–43.97)	41.26 (40.81–41.72)	38.70 (38.28–39.13)	35.53 (35.07–35.99)	39.65 (39.05–40.26)	33.56 (32.79–34.33)	37.78 (36.96–38.60)	40.41 (39.61–41.21)	**-1.46** **(-1.63﻿ to –1.30)**	**0.59 (0.27 to 0.91) **	**2.05 (1.69 to 2.41) **
		Mid-low	53.90 (53.21–54.60)	50.83 (50.15–51.51)	49.56 (48.89–50.23)	46.76 (45.98–47.55)	52.06 (51.00-53.12)	45.82 (44.29–47.35)	50.05 (48.50–51.60)	52.10 (50.47–53.73)	**-1.11** **(-1.37﻿ to –0.85)**	**0.30 (-0.31 to 0.91) **	**1.41 (0.75 to 2.07) **
		low	63.37 (62.36–64.39)	60.53 (59.42–61.63)	60.22 (59.02–61.42)	56.60 (55.07–58.12)	59.44 (57.26–61.63)	52.85 (49.71–55.99)	54.94 (51.63–58.25)	62.77 (59.42–66.13)	**-1.40** **(-1.87﻿ to –0.94)**	**0.98 (-0.29 to 2.24) **	**2.38 (1.03 to 3.73) **
Sex	Male	High	36.30 (35.03–37.57)	35.11 (33.86–36.37)	30.58 (29.56–31.60)	28.74 (27.87–29.62)	29.14 (27.99–30.28)	24.88 (23.10-26.67)	31.91 (30.12–33.69)	36.58 (34.72–38.44)	**-2.06** **(-2.43﻿ to –1.68)**	**2.86 (2.16 to 3.56) **	**4.92 (4.13 to 5.71) **
		Mid-high	35.86 (35.10-36.62)	32.59 (31.90-33.27)	29.29 (28.69–29.90)	27.67 (27.08–28.25)	28.95 (28.17–29.74)	25.97 (24.91–27.03)	31.08 (29.84–32.31)	33.41 (32.19–34.64)	**-1.92** **(-2.16﻿ to –1.69)**	**1.83 (1.37 to 2.30)**	**3.75 (3.23 to 4.27) **
		Middle	37.63 (37.04–38.23)	34.73 (34.18–35.28)	31.52 (31.03–32.01)	28.14 (27.67–28.61)	31.20 (30.54–31.85)	27.11 (26.17–28.06)	30.34 (29.41–31.28)	34.59 (33.63–35.56)	**-2.07** **(-2.26﻿ to –1.88)**	**1.28 (0.91 to 1.66)**	**3.35 (2.93 to 3.77) **
		Mid-low	47.29 (46.33–48.25)	43.35 (42.47–44.24)	41.32 (40.45–42.18)	39.25 (38.23–40.27)	42.18 (40.82–43.53)	37.96 (35.97–39.95)	43.56 (41.42–45.69)	46.32 (44.03–48.61)	**-1.75** **(-2.09﻿ to –1.40)**	**1.70 (0.87 to 2.53) **	**3.45 (2.55 to 4.35) **
		low	56.86 (55.50-58.22)	54.77 (53.32–56.22)	52.63 (51.01–54.26)	49.92 (47.79–52.06)	52.36 (49.59–55.12)	46.51 (42.57–50.46)	47.42 (43.18–51.67)	58.98 (54.72–63.23)	**-1.63** **(-2.24﻿ to –1.02)**	**1.81 (0.20 to 3.41) **	**3.44 (1.72 to 5.16) **
	Female	High	43.71 (41.93–45.48)	44.24 (42.50-45.98)	40.23 (38.76–41.70)	37.33 (36.13–38.54)	44.41 (42.83-46.00)	33.15 (30.89–35.40)	43.67 (41.36–45.98)	44.86 (42.83–46.90)	-0.49 (-1.00 ﻿to 0.03)	**1.30 (0.45 to 2.15)**	**1.79 (0.81 to 2.77) **
		Mid-high	47.18 (46.33–48.03)	43.99 (43.19–44.78)	42.09 (41.38–42.80)	39.95 (39.22–40.67)	46.49 (45.56–47.42)	38.29 (36.95–39.63)	42.76 (41.47–44.05)	44.92 (43.67–46.16)	**-0.58** **(-0.86﻿ to –0.31)**	**0.06 (-0.57 to 0.44)**	**0.64 (0.06 to 1.22) **
		Middle	49.57 (48.96–50.17)	47.80 (47.23–48.37)	45.81 (45.29–46.33)	42.74 (42.17–43.31)	47.93 (47.18–48.67)	39.84 (38.84–40.85)	44.96 (43.97–45.96)	46.04 (45.02–47.06)	**-0.95** **(-1.16﻿ to –0.75)**	**0.10 (-0.51 to 0.30)**	**1.05 (0.60 to 1.50) **
		Mid-low	60.72 (59.88–61.56)	58.42 (57.58–59.27)	57.73 (56.96–58.50)	54.47 (53.50-55.44)	61.61 (60.31–62.91)	54.11 (52.14–56.08)	57.05 (54.94–59.16)	57.63 (55.42–59.85)	**-0.56** **(-0.89﻿ to –0.24)**	**1.70 (0.87 to 2.53) **	**2.26 (1.37 to 3.15) **
		low	71.53 (70.12–72.93)	68.02 (66.52–69.52)	69.64 (68.13–71.14)	64.92 (62.88–66.96)	69.11 (65.93–72.30)	61.44 (56.66–66.23)	66.01 (61.28–70.74)	68.11 (62.89–73.33)	**-1.04** **(-1.69﻿ to –0.40)**	-0.03 (-1.93 to 1.88)	**1.01 (-1.00 to 3.02) **
**Sadness**													
Overall		High	37.27 (36.24–38.30)	34.41 (33.37–35.45)	26.96 (26.18–27.73)	22.86 (22.20-23.53)	24.93 (24.13–25.74)	22.32 (21.09–23.55)	25.85 (24.59–27.11)	28.15 (26.82–29.48)	**-3.58** **(-3.87﻿ to –3.29)**	**1.31 (0.81 to 1.81) **	**4.89 (4.31 to 5.47) **
		Mid-high	37.36 (36.77–37.95)	32.81 (32.27–33.36)	26.71 (26.26–27.17)	23.10 (22.66–23.53)	25.91 (25.33–26.49)	23.63 (22.84–24.42)	25.28 (24.50-26.06)	27.13 (26.32–27.94)	**-3.33** **(-3.51﻿ to –3.15)**	**0.52 (0.21 to 0.84) **	**3.85 (3.49 to 4.21) **
		Middle	38.39 (37.95–38.83)	33.59 (33.18-34.00)	27.42 (27.05–27.79)	23.22 (22.85–23.59)	26.47 (25.95–26.98)	24.29 (23.59–24.99)	25.62 (24.93–26.32)	27.49 (26.80-28.18)	**-3.60** **(-3.74﻿ to –3.45)**	**0.41 (0.14 to 0.69) **	**4.01 (3.70 to 4.32) **
		Mid-low	46.48 (45.76–47.19)	41.50 (40.85–42.15)	36.08 (35.46–36.69)	30.94 (30.28–31.59)	36.42 (35.40-37.45)	33.01 (31.64–34.37)	35.21 (33.69–36.74)	37.64 (36.07–39.20)	**-3.56** **(-3.81﻿ to –3.30)**	**0.49 (-0.09 to 1.08) **	**4.05 (3.41 to 4.69) **
		low	55.67 (54.62–56.73)	51.57 (50.43–52.70)	45.48 (44.36–46.59)	42.51 (41.11–43.91)	44.87 (42.80-46.93)	42.79 (39.85–45.74)	44.83 (41.48–48.19)	46.83 (43.32–50.34)	**-3.60** **(-4.05﻿ to –3.15)**	0.72 (-0.56 to 2.01)	**4.32 (2.96 to 5.68) **
Sex	Male	High	35.35 (34.05–36.64)	32.18 (30.97–33.40)	24.11 (23.19–25.03)	20.55 (19.75–21.34)	20.68 (19.74–21.62)	19.58 (18.05–21.11)	22.77 (21.22–24.32)	24.46 (22.74–26.18)	**-4.07** **(-4.42﻿ to –3.72)**	**1.42 (0.80 to 2.05) **	**5.49 (4.77 to 6.21) **
		Mid-high	33.28 (32.52–34.03)	28.47 (27.77–29.17)	22.35 (21.77–22.92)	19.01 (18.49–19.54)	20.36 (19.67–21.06)	18.38 (17.40-19.35)	21.82 (20.84–22.80)	22.75 (21.72–23.78)	**-3.60** **(-3.83﻿ to –3.38)**	**1.05 (0.65 to 1.45) **	**4.65 (4.19 to 5.11) **
		Middle	33.76 (33.20-34.32)	28.53 (28.03–29.04)	22.24 (21.80-22.68)	18.48 (18.04–18.91)	20.25 (19.68–20.82)	18.88 (18.05–19.71)	20.68 (19.82–21.54)	22.83 (21.95–23.71)	**-3.88** **(-4.05﻿ to –3.70)**	**0.93 (0.60 to 1.26) **	**4.81 (4.44 to 5.18) **
		Mid-low	40.34 (39.36–41.32)	34.65 (33.78–35.53)	29.76 (28.95–30.58)	25.05 (24.18–25.92)	28.70 (27.41-30.00)	26.95 (25.08–28.83)	28.92 (26.92–30.93)	31.73 (29.63–33.83)	**-3.73** **(-4.07﻿ to –3.40)**	**1.04 (0.26 to 1.81) **	**4.77 (3.93 to 5.61) **
		low	50.02 (48.56–51.48)	46.34 (44.96–47.73)	38.41 (36.95–39.87)	37.11 (35.25–38.97)	38.38 (35.76–41.01)	36.21 (32.43–39.99)	38.72 (34.57–42.87)	43.02 (38.46–47.58)	**-3.81** **(-4.40﻿ to –3.21)**	**1.54 (-0.12 to 3.19) **	**5.35 (3.59 to 7.11) **
	Female	High	40.76 (39.06–42.46)	39.00 (37.27–40.74)	32.32 (30.99–33.64)	26.73 (25.59–27.87)	32.13 (30.71–33.54)	26.22 (24.26–28.18)	30.44 (28.44–32.44)	33.20 (31.13–35.27)	**-2.88** **(-3.37﻿ to –2.40)**	**0.83 (0.03 to 1.64) **	**3.71 (2.77 to 4.65) **
		Mid-high	42.41 (41.50-43.31)	38.20 (37.42–38.98)	31.95 (31.28–32.62)	27.76 (27.10-28.41)	32.06 (31.22–32.90)	29.58 (28.43–30.73)	29.06 (27.88–30.25)	32.08 (30.92–33.23)	**-3.16** **(-3.43﻿ to –2.89)**	**-0.05 (-0.51 to 0.41) **	**3.11 (2.58 to 3.64) **
		Middle	43.18 (42.58–43.79)	38.64 (38.10-39.19)	32.54 (32.04–33.03)	27.85 (27.35–28.34)	32.56 (31.85–33.27)	29.55 (28.60-30.51)	30.40 (29.47–31.34)	32.00 (31.06–32.94)	**-3.39** **(-3.59﻿ to –3.19)**	**-0.11 (-0.49 to 0.26) **	**3.28 (2.86 to 3.71) **
		Mid-low	52.80 (51.89–53.71)	48.44 (47.62–49.27)	42.33 (41.50-43.16)	36.97 (36.05–37.90)	43.88 (42.53–45.22)	39.39 (37.44–41.34)	42.00 (39.77–44.23)	43.29 (41.12–45.45)	**-3.45** **(-3.79﻿ to –3.11)**	**-0.02 (-0.82 to 0.79) **	**3.43 (2.56 to 4.30) **
		low	62.76 (61.19–64.32)	58.36 (56.66–60.05)	54.24 (52.59–55.89)	49.24 (47.15–51.34)	53.72 (50.57–56.87)	51.72 (47.17–56.27)	53.84 (48.76–58.93)	52.18 (46.90-57.47)	**-3.28** **(-3.95﻿ to –2.60)**	-0.27 (-2.20 to 1.67)	**3.01 (0.96 to 5.06) **
**Suicidal ideation**													
Overall		High	20.81 (19.93–21.68)	19.31 (18.48–20.15)	15.41 (14.74–16.09)	11.37 (10.86–11.88)	11.80 (11.13–12.46)	8.71 (7.89–9.52)	10.87 (9.95–11.79)	13.92 (12.87–14.97)	**-2.59 (-2.83 to -2.35) **	**0.85 (0.46 to 1.25)**	**3.44 (2.98 to 3.90)**
		Mid-high	19.36 (18.89–19.82)	16.98 (16.56–17.41)	14.01 (13.68–14.35)	10.63 (10.32–10.95)	9.81 (9.23–10.39)	10.63 (10.32–10.95)	11.46 (10.89–12.04)	12.58 (11.94–13.22)	**-2.25 (-2.38 to -2.11)**	**0.50 (0.26 to 0.74)**	**2.75 (2.47 to 3.03)**
		Middle	20.02 (19.65–20.39)	17.14 (16.80-17.48)	14.16 (13.88–14.44)	10.58 (10.33–10.83)	12.24 (11.89–12.58)	10.10 (9.63–10.57)	11.90 (11.38–12.42)	13.27 (12.76–13.78)	**-2.30 (-2.41 to -2.19)**	**0.47 (0.27 to 0.66)**	**2.77 (2.55 to 2.99)**
		Mid-low	26.62 (26.01–27.23)	23.74 (23.18–24.30)	20.90 (20.38–21.42)	17.00 (16.46–17.55)	20.63 (19.79–21.47)	16.94 (15.81–18.07)	20.03 (18.74–21.33)	22.25 (20.87–23.64)	**-2.15 (-2.36 to -1.93)**	**0.70 (0.20 to 1.21)**	**2.85 (2.30 to 3.40)**
		low	36.14 (35.09–37.18)	33.21 (32.14–34.29)	30.62 (29.59–31.65)	26.67 (25.38–27.96)	28.79 (26.85–30.73)	24.21 (21.56–26.87)	28.61 (25.68–31.55)	31.70 (28.44–34.96)	**-2.39 (-2.82 to -1.96)**	**1.18 (-0.01 to 2.37)**	**3.57 (2.30 to 4.84)**
Sex	Male	High	19.35 (18.28–20.42)	17.62 (16.63–18.61)	13.62 (12.85–14.39)	10.10 (9.46–10.73)	9.39 (8.65–10.13)	7.62 (6.64–8.61)	8.73 (7.63–9.84)	10.94 (9.62–12.26)	**-2.74 (-3.03 to -2.46)**	**0.55 (0.07 to 1.03) **	**3.29 (2.73 to 3.85) **
		Mid-high	16.24 (15.69–16.80)	13.77 (13.26–14.29)	11.40 (10.97–11.82)	8.45 (8.07–8.83)	6.87 (6.27–7.47)	8.45 (8.07–8.83)	8.99 (8.28–9.70)	9.66 (8.91–10.41)	** -2.20 (-2.35 to -2.04)**	**0.69 (0.41 to 0.97) **	**2.89 (2.57 to 3.21) **
		Middle	16.01 (15.60-16.42)	13.24 (12.84–13.63)	10.92 (10.58–11.26)	8.21 (7.91–8.51)	8.47 (8.09–8.85)	7.49 (6.90–8.08)	8.54 (7.96–9.12)	9.89 (9.27–10.51)	**-2.07 (-2.19 to -1.94)**	**0.52 (0.29 to 0.75) **	**2.59 (2.33 to 2.85) **
		Mid-low	20.61 (19.82–21.40)	17.81 (17.12–18.50)	16.11 (15.48–16.75)	12.84 (12.17–13.51)	14.70 (13.67–15.72)	12.35 (11.02–13.68)	14.33 (12.81–15.85)	16.80 (15.10-18.49)	**-1.86 (-2.13 to -1.59)**	**0.76 (0.14 to 1.37) **	**2.62 (1.93 to 3.31) **
		low	29.82 (28.49–31.15)	27.40 (26.10-28.71)	24.91 (23.64–26.18)	22.86 (21.24–24.48)	23.92 (21.46–26.38)	18.54 (15.34–21.74)	22.39 (19.06–25.71)	24.83 (20.80-28.86)	**-1.86 (-2.40 to -1.31)**	**0.52 (-0.94 to 1.99) **	**2.38 (0.82 to 3.94) **
	Female	High	23.45 (21.97–24.93)	22.81 (21.28–24.34)	18.79 (17.56–20.03)	13.50 (12.63–14.36)	15.87 (14.70-17.04)	10.25 (8.84–11.65)	14.05 (12.53–15.57)	18.01 (16.35–19.67)	**-2.42 (-2.84 to -2.00)**	**1.12 (0.46 to 1.78) **	**3.54 (2.76 to 4.32)**
		Mid-high	23.20 (22.42–23.98)	20.97 (20.33–21.60)	17.15 (16.63–17.67)	13.13 (12.63–13.63)	13.14 (12.24–14.05)	13.13 (12.63–13.63)	14.16 (13.27–15.06)	15.88 (14.93–16.82)	**-2.41 (-2.62 to -2.19)**	**0.29 (-0.07 to 0.66) **	**2.70 (2.28 to 3.12)**
		Middle	24.17 (23.63–24.72)	21.04 (20.58–21.50)	17.36 (16.96–17.77)	12.88 (12.51–13.25)	15.93 (15.41–16.45)	12.64 (11.93–13.34)	15.14 (14.37–15.92)	16.54 (15.77–17.30)	**-2.58 (-2.74 to -2.41)**	**0.41 (0.11 to 0.70) **	**2.99 (2.65 to 3.33)**
		Mid-low	32.81 (31.93–33.69)	29.76 (28.99–30.53)	25.64 (24.89–26.39)	21.27 (20.44–22.11)	26.36 (25.16–27.56)	21.78 (20.03–23.52)	26.19 (24.24–28.14)	27.47 (25.49–29.46)	**-2.50 (-2.81 to -2.18)**	**0.67 (-0.07 to 1.40) **	**3.17 (2.37 to 3.97)**
		low	44.05 (42.44–45.67)	40.77 (39.25–42.29)	37.70 (36.13–39.28)	31.42 (29.36–33.48)	35.44 (32.39–38.48)	31.91 (27.55–36.26)	37.78 (33.10-42.47)	41.36 (36.17–46.56)	**-3.01 (-3.68 to -2.35)**	**2.23 (0.35 to 4.11) **	**5.24 (3.25 to 7.23)**
**Suicide attempt**													
Overall		High	7.11 (6.58–7.63)	6.31 (5.80–6.82)	4.98 (4.57–5.39)	3.30 (3.01–3.58)	3.31 (2.98–3.63)	1.90 (1.53–2.28)	2.30 (1.85–2.76)	3.42 (2.90–3.93)	**-1.06** **(-1.20 to -0.92)**	**0.07 (-0.12 to 0.27)**	**1.13 (0.89 to 1.37) **
		Mid-high	4.59 (4.35–4.82)	3.81 (3.60–4.02)	3.03 (2.86–3.20)	2.04 (1.91–2.17)	2.32 (2.14–2.51)	1.78 (1.52–2.03)	1.80 (1.57–2.03)	2.14 (1.87–2.41)	**-0.64** **(-0.70 to -0.57)**	**-0.06 (-0.16 to 0.05)**	**0.58 (0.46 to 0.70)**
		Middle	4.59 (4.35–4.82)	3.81 (3.60–4.02)	3.03 (2.86–3.20)	2.04 (1.91–2.17)	2.32 (2.14–2.51)	1.78 (1.52–2.03)	1.80 (1.57–2.03)	2.14 (1.87–2.41)	**-0.55** **(-0.60 to -0.50)**	**-0.08 (-0.16 to 0.01) **	**0.47 (0.37 to 0.57) **
		Mid-low	6.29 (5.95–6.63)	5.46 (5.15–5.76)	4.39 (4.15–4.64)	3.35 (3.08–3.61)	5.21 (4.78–5.64)	3.44 (2.93–3.95)	4.02 (3.39–4.64)	3.79 (3.18–4.41)	**-0.55** **(-0.67 to -0.44)**	-0.39 (-0.63 to -0.16)	**0.16 (-0.10 to 0.42) **
		low	11.65 (10.94–12.36)	11.28 (10.59–11.97)	9.87 (9.20-10.55)	8.44 (7.65–9.24)	10.12 (8.82–11.43)	6.31 (4.79–7.82)	8.23 (6.62–9.83)	10.45 (8.46–12.45)	**-0.72** **(-1.00 to -0.43)**	**0.19 (-0.55 to 0.93) **	**0.91 (0.12 to 1.70) **
Sex	Male	High	6.76 (6.13–7.39)	5.83 (5.22–6.44)	4.46 (4.00-4.92)	3.23 (2.88–3.59)	2.86 (2.47–3.26)	1.83 (1.34–2.32)	1.72 (1.27–2.18)	2.92 (2.26–3.59)	**-1.04** **(-1.20 to -0.87)**	**0.00 (-0.24 to 0.23) **	**1.04 (0.75 to 1.33) **
		Mid-high	3.82 (3.54–4.10)	2.65 (2.42–2.89)	2.13 (1.94–2.33)	1.59 (1.43–1.74)	1.61 (1.40–1.82)	1.13 (0.87–1.39)	1.23 (0.95–1.51)	1.55 (1.25–1.86)	**-0.56** **(-0.63 to -0.48)**	**-0.01 (-0.12 to 0.11) **	**0.55 (0.41 to 0.69) **
		Middle	3.82 (3.54–4.10)	2.65 (2.42–2.89)	2.13 (1.94–2.33)	1.59 (1.43–1.74)	1.61 (1.40–1.82)	1.13 (0.87–1.39)	1.23 (0.95–1.51)	1.55 (1.25–1.86)	-0.04 (-0.50 to -0.43)	**0.03 (-0.06 to 0.13) **	**0.07 (-0.40 to 0.54) **
		Mid-low	4.27 (3.89–4.66)	3.50 (3.18–3.82)	2.77 (2.49–3.05)	2.36 (2.03–2.69)	3.15 (2.67–3.63)	2.10 (1.49–2.70)	2.29 (1.69–2.90)	2.51 (1.78–3.23)	**-0.42** **(-0.55 to -0.29)**	-0.19 (-0.46 to 0.08)	**0.23 (-0.07 to 0.53) **
		low	9.82 (8.93–10.71)	9.18 (8.35–10.02)	7.60 (6.76–8.44)	7.13 (6.09–8.17)	8.61 (7.03–10.18)	4.03 (2.60–5.46)	6.96 (4.91-9.00)	8.12 (5.66–10.58)	**-0.62** **(-0.98 to -0.26)**	**0.05 (-0.85 to 0.95) **	**0.67 (-0.30 to 1.64) **
	Female	High	7.74 (6.84–8.64)	7.30 (6.33–8.26)	5.96 (5.18–6.74)	3.40 (2.92–3.88)	4.05 (3.46–4.65)	2.01 (1.40–2.61)	3.17 (2.33–4.01)	4.10 (3.21–4.98)	**-1.12** **(-1.36 to -0.88)**	**0.16 (-0.18 to 0.50) **	**1.28 (0.86 to 1.70) **
		Mid-high	5.53 (5.17–5.90)	5.25 (4.89–5.60)	4.11 (3.82–4.40)	2.57 (2.34–2.79)	3.12 (2.82–3.42)	2.51 (2.10–2.92)	2.42 (2.04–2.79)	2.79 (2.38–3.21)	**-0.76** **(-0.86 to -0.66)**	-0.11 (-0.27 to 0.06)	**0.65 (0.46 to 0.84) **
		Middle	5.53 (5.17–5.90)	5.25 (4.89–5.60)	4.11 (3.82–4.40)	2.57 (2.34–2.79)	3.12 (2.82–3.42)	2.51 (2.10–2.92)	2.42 (2.04–2.79)	2.79 (2.38–3.21)	**-0.48** **(-0.53 to -0.42)**	**-0.19 (-0.32 to -0.06) **	**0.29 (0.15 to 0.43) **
		Mid-low	8.37 (7.80–8.95)	7.44 (6.97–7.92)	6.00 (5.60–6.40)	4.36 (3.96–4.76)	7.20 (6.52–7.88)	4.86 (4.00-5.72)	5.88 (4.79–6.96)	5.03 (4.10–5.95)	**-0.71** **(-0.89 to -0.52)**	-0.58 (-0.95 to -0.22)	**0.13 (-0.28 to 0.54) **
		low	13.93 (12.80-15.07)	14.00 (12.88–15.13)	12.70 (11.59–13.80)	10.09 (8.82–11.35)	12.20 (10.05–14.34)	9.40 (6.68–12.12)	10.10 (7.22–12.97)	13.73 (10.19–17.27)	**-0.82** **(-1.28 to -0.36)**	0.43 (-0.85 to 1.70)	**1.25 (-0.11 to 2.61) **

Abbreviations: CI, Confidence Interval; KYRBS, the Korea Youth Risk Behavior Survey.

Numbers in bold indicate a significant difference (p< 0.05).

**Table 3:** Comparative analysis of adolescents’ mental health indicators across various periods by household income levels, KYRBS, 2006–2022 wOR (95% CI).

Variables		Before Pandemic					During Pandemic		
		2006–2008	2009–2011	2012–2014	2015–2017	2018–2019	2020	2021	2022
Perceived stress level	High	**0.83** **(0.79–0.87)**	**0.88** **(0.84–0.92)**	**0.81** **(0.78–0.85)**	**0.85** **(0.82–0.89)**	**0.81** **(0.78–0.85)**	**0.78** **(0.72–0.84)**	0.95 (0.89–1.02)	0.99 (0.93–1.05)
	Mid-high	**0.90** **(0.88–0.93)**	**0.86** **(0.84–0.88)**	**0.86** **(0.84–0.88)**	**0.91** **(0.89–0.93)**	**0.90** **(0.88–0.93)**	**0.92** **(0.88–0.96)**	0.95 (0.91-1.00)	**0.94** **(0.89–0.98)**
	Middle	1.00 (Reference)	1.00 (Reference)	1.00 (Reference)	1.00 (Reference)	1.00 (Reference)	1.00 (Reference)	1.00 (Reference)	1.00 (Reference)
	Mid-low	**1.52** **(1.47–1.57)**	**1.47** **(1.43–1.51)**	**1.56** **(1.51–1.60)**	**1.59** **(1.54–1.65)**	**1.65** **(1.59–1.72)**	**1.67** **(1.57–1.79)**	**1.65** **(1.54–1.76)**	**1.60** **(1.50–1.72)**
	Low	**2.25** **(2.15–2.35)**	**2.18** **(2.08–2.29)**	**2.40** **(2.28–2.52)**	**2.37** **(2.22–2.52)**	**2.23** **(2.04–2.44)**	**2.22** **(1.95–2.52)**	**2.01** **(1.76–2.30)**	**2.49** **(2.15–2.87)**
Sadness	High	0.95 (0.91-1.00)	1.04 (0.99–1.09)	0.98 (0.94–1.02)	0.98 (0.94–1.02)	**0.92** **(0.88–0.97)**	**0.90** **(0.83–0.97)**	1.01 (0.94–1.09)	1.03 (0.96–1.11)
	Mid-high	**0.96** **(0.93–0.99)**	**0.97** **(0.94–0.99)**	**0.97** **(0.94–0.99)**	0.99 (0.97–1.02)	0.97 (0.94–1.01)	0.97 (0.92–1.01)	0.98 (0.94–1.03)	0.98 (0.94–1.03)
	Middle	1.00 (Reference)	1.00 (Reference)	1.00 (Reference)	1.00 (Reference)	1.00 (Reference)	1.00 (Reference)	1.00 (Reference)	1.00 (Reference)
	Mid-low	**1.39** **(1.35–1.44)**	**1.40** **(1.36–1.44)**	**1.49** **(1.45–1.54)**	**1.48** **(1.43–1.53)**	**1.59** **(1.52–1.67)**	**1.54** **(1.44–1.64)**	**1.58** **(1.47–1.70)**	**1.59** **(1.48–1.71)**
	Low	**2.02** **(1.93–2.11)**	**2.11** **(2.01–2.21)**	**2.21** **(2.11–2.31)**	**2.45** **(2.30–2.60)**	**2.26** **(2.08–2.46)**	**2.33** **(2.06–2.64)**	**2.36** **(2.06–2.71)**	**2.32** **(2.01–2.69)**
Suicidal ideation	High	1.05 (0.99–1.11)	**1.16** **(1.09–1.23)**	**1.11** **(1.05–1.17)**	**1.09** **(1.03–1.15)**	0.96 (0.90–1.03)	**0.85** **(0.76–0.95)**	0.90 (0.82-1.00)	1.06 (0.96–1.16)
	Mid-high	**0.96** **(0.93–0.99)**	0.99 (0.96–1.02)	0.99 (0.96–1.02)	1.01 (0.97–1.05)	**0.93** **(0.89–0.97)**	0.97 (0.90–1.04)	0.96 (0.89–1.03)	0.94 (0.88-1.00)
	Middle	1.00 (Reference)	1.00 (Reference)	1.00 (Reference)	1.00 (Reference)	1.00 (Reference)	1.00 (Reference)	1.00 (Reference)	1.00 (Reference)
	Mid-low	**1.45** **(1.40–1.50)**	**1.51** **(1.46–1.56)**	**1.60** **(1.55–1.66)**	**1.73** **(1.66–1.81)**	**1.86** **(1.76–1.97)**	**1.82** **(1.65–1.99)**	**1.86** **(1.69–2.04)**	**1.87** **(1.72–2.04)**
	Low	**2.26** **(2.15–2.37)**	**2.40** **(2.29–2.53)**	**2.68** **(2.54–2.82)**	**3.08** **(2.87–3.30)**	**2.90** **(2.63–3.20)**	**2.85** **(2.44–3.32)**	**2.97** **(2.57–3.43)**	**3.03** **(2.59–3.56)**
Suicide attempt	High	**1.68** **(1.54–1.84)**	**1.79** **(1.62–1.97)**	**1.66** **(1.51–1.83)**	**1.74** **(1.57–1.93)**	**1.31** **(1.17–1.47)**	1.13 (0.90–1.41)	1.25 (1.00-1.55)	**1.53** **(1.27–1.85)**
	Mid-high	1.06 (0.99–1.13)	1.05 (0.98–1.13)	0.99 (0.93–1.06)	1.07 (0.98–1.16)	0.91 (0.83–1.01)	1.05 (0.89–1.25)	0.97 (0.83–1.13)	0.95 (0.81–1.10)
	Middle	1.00 (Reference)	1.00 (Reference)	1.00 (Reference)	1.00 (Reference)	1.00 (Reference)	1.00 (Reference)	1.00 (Reference)	1.00 (Reference)
	Mid-low	**1.48** **(1.38–1.58)**	**1.53** **(1.43–1.64)**	**1.46** **(1.36–1.56)**	**1.77** **(1.61–1.95)**	**2.11** **(1.91–2.33)**	**2.07** **(1.71–2.50)**	**2.21** **(1.83–2.67)**	**1.71** **(1.42–2.05)**
	Low	**2.90** **(2.68–3.14)**	**3.37** **(3.11–3.66)**	**3.47** **(3.19–3.78)**	**4.72** **(4.20–5.30)**	**4.32** **(3.72–5.03)**	**3.91** **(2.95–5.20)**	**4.74** **(3.75–5.98)**	**5.06** **(4.01–6.38)**

Abbreviations: CI, Confidence Interval; KYRBS, the Korea Youth Risk Behavior Survey; wOR, weighted odds ratio.

Numbers in bold indicate a significant difference (*p* < 0.05).

**Fig. 1:**
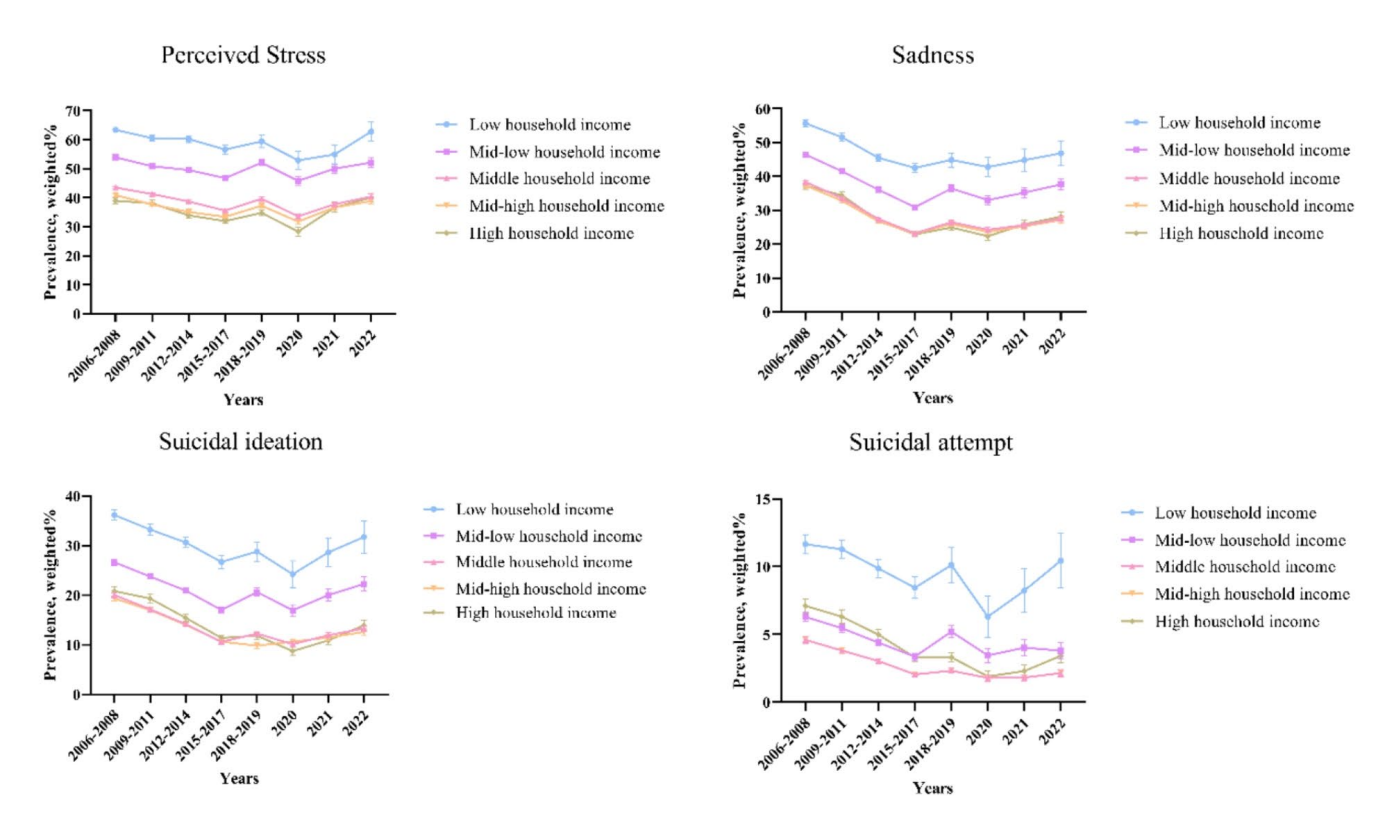
Trends in four mental health indicators by income groups, weighted %, 2006–2022.

Not just in 2022, but throughout various periods, a consistent relationship was observed between household income levels and mental health indicators. Furthermore, all four mental health indicators experienced a decline prior to the COVID–19 pandemic, followed by a notable increase during the pandemic, illustrating a U-shaped curve as depicted in Fig. 1. In the period of the pandemic, there was an escalation in perceived stress, sadness, suicidal ideation, and suicide attempt when compared to the year before. As a reference, the middle income group shows an increasing pattern among four indicators: stress (wOR, 1.12; 95% CI, 1.06 to 1.17), sadness (1.07; 1.02 to 1.13), suicidal ideation (1.20; 1.02 to 1.13), and suicide attempt (1.10; 0.95 to 1.27) when comparing 2021 with 2020, and similarly, stress (1.20; 1.14 to 1.26), sadness (1.10; 1.05–1.16), suicidal ideation (1.13; 1.06 to 1.21), and suicide attempt (1.22; 1.06 to 1.40) when comparing 2022 with 2021 (Table3 and Table S4).

**Table 4:** Comparative analysis of four adolescents’ mental health indicators using wOR (95% CI) by household income level, KYRBS, 2006–2022.

Variables			Perceived stress level						Sadness						Suicidal ideation						Suicide attempt					
			2020 vs. 2019		2021 vs. 2020		2022 vs. 2021		2020 vs. 2019		2021 vs. 2020		2022 vs. 2021		2020 vs. 2019		2021 vs. 2020		2022 vs. 2021		2020 vs. 2019		2021 vs. 2020		2022 vs. 2021	
			wOR (95% CI)	p-value	wOR (95% CI)	p-value	wOR (95% CI)	p-value	wOR (95% CI)	p-value	wOR (95% CI)	p-value	wOR (95% CI)	p-value	wOR (95% CI)	p-value	wOR (95% CI)	p-value	wOR (95% CI)	p-value	wOR (95% CI)	p-value	wOR (95% CI)	p-value	wOR (95% CI)	p-value
Overall		High	**0.75 (0.68–0.83)**	**< 0.001**	**1.47 (1.33–1.61)**	**< 0.001**	**1.16 (1.06–1.26)**	**< 0.001**	**0.85 (0.77–0.93)**	**< 0.001**	**1.21 (1.10–1.34)**	**< 0.001**	**1.12 (1.02–1.23)**	**0.014**	**0.75 (0.66–0.86)**	**< 0.001**	**1.28 (1.11–1.47)**	**< 0.001**	**1.33 (1.17–1.51)**	**< 0.001**	**0.58 (0.46–0.75)**	**< 0.001**	1.22 (0.92–1.61)	0.178	**1.50 (1.16–1.94)**	**0.002**
		Mid-high	**0.78 (0.74–0.83)**	**< 0.001**	**1.24 (1.17–1.32)**	**< 0.001**	**1.10 (1.04–1.16)**	**0.001**	**0.85 (0.80–0.90)**	**< 0.001**	**1.09 (1.03–1.16)**	**0.004**	**1.10 (1.04–1.17)**	**0.001**	**0.85 (0.78–0.92)**	**< 0.001**	**1.19 (1.09–1.30)**	**< 0.001**	**1.11 (1.03–1.21)**	**0.010**	**0.76 (0.63–0.92)**	**0.004**	1.01 (0.83–1.23)	0.908	1.19 (0.99–1.43)	0.059
		Middle	1.20 (1.14–1.26)	< 0.001	**1.12 (1.06–1.17)**	**< 0.001**	**1.20 (1.14–1.26)**	**< 0.001**	**0.87 (0.83–0.92)**	**< 0.001**	**1.07 (1.02–1.13)**	**0.008**	**1.10 (1.05–1.16)**	**< 0.001**	**0.81 (0.76–0.87)**	**< 0.001**	**1.20 (1.12–1.29)**	**< 0.001**	**1.13 (1.06–1.21)**	**< 0.001**	**0.70 (0.61–0.80)**	**< 0.001**	1.10 (0.95–1.27)	0.192	**1.22 (1.06–1.40)**	**0.005**
		Mid-low	**0.79 (0.73–0.87)**	**< 0.001**	**1.19 (1.09–1.29)**	**< 0.001**	1.09 (0.99–1.19)	0.074	**0.84 (0.77–0.92)**	**< 0.001**	**1.10 (1.01–1.21)**	**0.034**	**1.11 (1.01–1.22)**	**0.030**	**0.78 (0.70–0.87)**	**< 0.001**	**1.23 (1.10–1.38)**	**< 0.001**	**1.14 (1.02–1.28)**	**0.021**	**0.63 (0.51–0.77)**	**< 0.001**	1.17 (0.94–1.47)	0.160	0.94 (0.75–1.19)	0.620
		low	**0.81 (0.68–0.98)**	**0.029**	1.09 (0.91–1.31)	0.370	**1.38 (1.14–1.68)**	**0.001**	**0.83 (0.70–0.98)**	**0.028**	1.09 (0.91–1.30)	0.369	1.08 (0.89–1.32)	0.420	**0.74 (0.61–0.90)**	**0.003**	**1.25 (1.02–1.54)**	**0.029**	1.16 (0.94–1.43)	0.166	**0.57 (0.41–0.79)**	**< 0.001**	1.33 (0.96–1.86)	0.092	1.30 (0.96–1.76)	0.085
Sex	Male	High	**0.81 (0.71–0.92)**	**0.001**	**1.41 (1.25–1.60)**	**< 0.001**	**1.23 (1.10–1.38)**	**< 0.001**	0.90 (0.79–1.02)	0.089	**1.21 (1.06–1.38)**	**0.004**	1.10 (0.97–1.25)	0.152	0.87 (0.73–1.05)	0.159	1.16 (0.95–1.41)	0.141	**1.28 (1.06–1.56)**	**0.012**	**0.63 (0.45–0.88)**	**0.007**	0.94 (0.64–1.38)	0.749	**1.72 (1.20–2.45)**	**0.003**
		Mid-high	**0.88 (0.81–0.95)**	**< 0.001**	**1.29 (1.19–1.39)**	**< 0.001**	**1.11 (1.03–1.21)**	**0.008**	**0.85 (0.78–0.93)**	**< 0.001**	**1.24 (1.14–1.35)**	**< 0.001**	1.06 (0.97–1.15)	0.201	**0.86 (0.76–0.98)**	**0.026**	**1.34 (1.18–1.52)**	**< 0.001**	1.08 (0.96–1.22)	0.205	0.78 (0.58–1.06)	0.112	1.09 (0.79–1.51)	0.606	1.27 (0.94–1.72)	0.125
		Middle	**0.82 (0.77–0.87)**	**< 0.001**	**1.17 (1.10–1.25)**	**< 0.001**	**1.21 (1.14–1.29)**	**< 0.001**	**0.89 (0.83–0.96)**	**0.003**	**1.12 (1.04–1.21)**	**0.003**	**1.14 (1.06–1.22)**	**< 0.001**	**0.88 (0.78–0.98)**	**0.017**	**1.15 (1.03–1.29)**	**0.013**	**1.18 (1.06–1.30)**	**0.002**	**0.76 (0.59–0.97)**	**0.029**	1.20 (0.93–1.53)	0.159	**1.27 (1.01–1.60)**	**0.044**
		Mid-low	**0.83 (0.74–0.92)**	**< 0.001**	**1.26 (1.12–1.42)**	**< 0.001**	1.12 (0.99–1.27)	0.084	0.90 (0.78–1.02)	0.108	1.10 (0.96–1.26)	0.159	1.14 (1.00-1.31)	0.058	**0.80 (0.67–0.95)**	**0.010**	1.19 (1.00-1.41)	0.054	**1.21 (1.02–1.44)**	**0.033**	0.71 (0.49–1.03)	0.070	1.10 (0.74–1.63)	0.654	1.10 (0.73–1.64)	0.655
		low	0.86 (0.69–1.08)	0.195	1.04 (0.82–1.31)	0.758	**1.59 (1.25–2.04)**	**< 0.001**	**0.78 (0.62–0.99)**	**0.037**	1.11 (0.88–1.41)	0.380	1.20 (0.93–1.54)	0.171	**0.66 (0.50–0.88)**	**0.005**	1.27 (0.95–1.69)	0.104	1.15 (0.86–1.53)	0.356	**0.43 (0.27–0.68)**	**< 0.001**	**1.78 (1.10–2.89)**	**0.020**	1.18 (0.75–1.87)	0.472
	Female	High	**0.65 (0.56–0.74)**	**< 0.001**	**1.56 (1.36–1.80)**	**< 0.001**	1.05 (0.93–1.19)	0.448	**0.76 (0.66–0.87)**	**< 0.001**	**1.23 (1.07–1.41)**	**0.003**	1.14 (1.00-1.30)	0.060	**0.61 (0.51–0.75)**	**< 0.001**	**1.43 (1.18–1.75)**	**< 0.001**	**1.34 (1.14–1.59)**	**0.001**	**0.52 (0.35–0.76)**	**< 0.001**	**1.60 (1.06–2.41)**	**0.026**	1.30 (0.92–1.86)	0.140
		Mid-high	**0.70 (0.65–0.76)**	**< 0.001**	**1.20 (1.11–1.30)**	**< 0.001**	**1.09 (1.02–1.17)**	**0.018**	**0.85 (0.78–0.91)**	**< 0.001**	0.98 (0.90–1.06)	0.537	**1.15 (1.07–1.25)**	**< 0.001**	**0.84 (0.75–0.93)**	**0.001**	1.09 (0.98–1.22)	0.117	**1.14 (1.03–1.27)**	**0.010**	**0.76 (0.61–0.94)**	**0.011**	0.96 (0.76–1.21)	0.745	1.16 (0.93–1.45)	0.190
		Middle	**0.73 (0.69–0.78)**	**< 0.001**	**1.23 (1.16–1.31)**	**< 0.001**	1.04 (0.99–1.11)	0.140	**0.85 (0.80–0.91)**	**< 0.001**	1.04 (0.98–1.11)	0.212	**1.08 (1.01–1.15)**	**0.018**	**0.78 (0.72–0.85)**	**< 0.001**	**1.23 (1.13–1.35)**	**< 0.001**	**1.11 (1.02–1.21)**	**0.012**	**0.68 (0.58–0.80)**	**< 0.001**	1.06 (0.89–1.25)	0.530	**1.20 (1.01–1.42)**	**0.040**
		Mid-low	**0.78 (0.69–0.87)**	**< 0.001**	**1.13 (1.00-1.27)**	**0.046**	1.02 (0.90–1.16)	0.709	**0.81 (0.72–0.91)**	**< 0.001**	1.11 (0.99–1.26)	0.084	1.05 (0.93–1.20)	0.417	**0.78 (0.67–0.89)**	**< 0.001**	**1.27 (1.10–1.47)**	**0.001**	1.07 (0.93–1.23)	0.364	**0.61 (0.48–0.77)**	**< 0.001**	1.22 (0.93–1.60)	0.145	0.85 (0.64–1.12)	0.241
		low	**0.72 (0.54–0.97)**	**0.028**	1.22 (0.91–1.63)	0.184	1.10 (0.80–1.51)	0.559	0.87 (0.67–1.13)	0.290	1.09 (0.83–1.43)	0.541	0.94 (0.70–1.26)	0.657	0.81 (0.61–1.08)	0.150	1.30 (0.98–1.72)	0.072	1.16 (0.87–1.56)	0.315	0.70 (0.45–1.07)	0.102	1.08 (0.69–1.70)	0.729	1.42 (0.92–2.19)	0.116

Abbreviations: CI, Confidence Interval; KYRBS, the Korea Youth Risk Behavior Survey; wOR, weighted odds ratio.

Numbers in bold indicate a significant difference (p< 0.05).

From 2006 to 2022, significant risk factors for mental health indicators included female sex, high school enrollment, urban residency, alcohol consumption, smoking, higher educational levels of parents, and superior academic performance of students (Table S5). Especially, female sex, alcohol consumption, and smoking status represents tremendous effect to the indicators. Within the middle income group, as a reference point, female sex participants have overall odds ratios (OR) of 1.68 (1.60 to 1.75), 1.63 (1.56 to 1.71), 1.65 (1.60 to 1.69), 1.99(1.71 to 2.32) for stress, sadness, suicidal ideation, and suicide attempts, respectively, compared to male sex participants. Additionally, for drinking, the OR values are 1.40 (1.31 to 1.50), 1.98 (1.85 to 2.12), 1.91 (1.86 to 1.97), 3.05 (2.56 to 3.63). Similarly, for smoking participants, the OR values are 1.56 (1.37 to 1.77) for stress, 2.50 (2.21 to 2.83) for sadness, 2.08 (2.00 to 2.17) for suicidal ideation, and 5.06 (3.98 to 6.45) for suicide attempts.

## Discussion

### Key findings

This study is the first long-term follow-up over 17 years to examine the relationship between household income level and mental health among adolescents, as well as their trends. Our findings showed that lower household income levels are associated with mental health indicators such as stress, sadness, suicidal ideation, and suicide attempts among Korean adolescents. Furthermore, our study indicated that the COVID–19 pandemic has significantly altered the trend in adolescent mental well-being. While there was a decreasing pattern of negative mental health indicators in the pre-pandemic era, there was a surge and a shift to an increasing pattern during the pandemic. This underscores the substantial impact of the COVID–19 pandemic on adolescent mental well-being, emphasizing the need to address not only physical symptoms like respiratory issues but also the mental health challenges faced by adolescents. In addition, we identified the most significant risk factors for negative mental health as being female, alcohol consumption, and smoking status.

### Plausible mechanism

We found that students from lower-income households tended to experience higher levels of stress, sadness, suicidal ideation, and suicide attempts. This could stem from feelings of relative deprivation compared to their peers and the recent social trend that places high importance on wealth^28^. Adolescents from lower-income households may feel disconnected from their higher-income peers, which can lead to low self-esteem and psychologically depressive thoughts and behaviors.

Additionally, the COVID-19 pandemic has exacerbated these issues by highlighting and intensifying existing inequalities. During lockdown periods, lower-income students were more likely to experience cramped living conditions, limited privacy, and difficulties accessing online education, potentially intensifying feelings of stress and isolation^29^. Additionally, lower-income families were more likely to experience job losses or reduced work hours during the pandemic, further increasing financial stress and its impact on adolescent mental health^30^.

In addition, parental psychological distress, rather than socioeconomic status itself, could contribute to these outcomes^31^. Parental care is crucial for the development of adolescents’ identity, and poor parental care can lead to psychological instability in their children. In collectivistic cultures like Korea, family plays a significant role in interpersonal relationships, and poor family function is closely linked to poor mental health in children^32^. This phenomenon can result in adolescents feeling isolated, with fewer peers around them^33^. School factors also play a role, as adolescents’ self-esteem related to school social capital has a similar impact on their mental health as parental factors^34^. Another potential mechanism is the increased academic pressure in lower-income families, where adolescents might feel a greater need to succeed academically to improve their future prospects^35^.

Additionally, female sex, alcohol consumption, and smoking status are established risk factors for poor mental health in previous studies. This trend is particularly pronounced among adolescents from low-income backgrounds^36–38^. These adolescents may engage in these risky behaviors as coping mechanisms to deal with the heightened stress and psychological burden they face, which further exacerbates their mental health issues^39^. The interplay between these factors creates a vicious cycle that is difficult to break, underscoring the need for targeted interventions that address both the socioeconomic and behavioral aspects of adolescent mental health.

These combined factors contribute to the negative mental health outcomes observed in adolescents from lower-income households.

### Comparison with previous studies

Several studies have reported the association between household income and adolescents’ mental health, including research in Kenya (*n* = 2,195), Norway (*n* = 1,354,393), Canada (*n* = 29,722), and Germany (*n* = 2,111)^10,40-42^. While these studies indicated an association between lower household income levels and increased instances of mental illness among adolescents, they were limited by their small sample sizes and the broad range of participant ages, which included both children and adolescents. In contrast, our investigation is distinguished by its large population-based design, specifically focusing on the age group of 12 to 18 years. Furthermore, our research extends over 17 years, providing insights into the long-term trends and patterns concerning the mental well-being of adolescents. Additionally, we divided income into five groups, which provides a detailed understanding of the impact of income inequality on adolescents’ mental health in South Korea. This approach allows the government or society to implement political interventions and support programs tailored to the specific needs of each income group. By considering both the COVID–19 pandemic and income levels, our study offers a comprehensive understanding of how external factors like the pandemic differently affect each income group. This underscores the necessity of a comprehensive understanding when examining the impact of the pandemic on changes in mental health indicators.

### Policy implications

Several previous studies have mainly focused on predicting and preventing suicidal behavior in adolescents and identifying related factors^43^. However, our study emphasizes that perceived stress levels and sadness are also crucial indicators of adolescents’ mental health, as they can lead to suicidal behavior^44^. Adolescents are vulnerable and can be influenced by various factors, so it is essential for governments and schools to focus on these negative mental health indicators^45^. Especially, we discovered female sex, alcohol, and smoking are significant risk factors for the negative mental health indicators. Therefore, find the reason why lower income female students are fragile to mental illness and solution for this. Also, school- or society-based substances abuse prevention programs, like alcohol and tobacco, needed to be prepared delicately for the risky teenagers^46^.

Moreover, numerous efforts aimed at improving adolescents’ mental health have led to a reduction in the overall incidence of mental health issues among this group^47,48^. However, the advent of the pandemic has precipitated a significant increase in these mental health challenges^2,17^. This issue demands urgent and serious discussion and resolution. Addressing the mental health of adolescents is critical, necessitating a collaborative effort from both governments and educational institutions to support students afflicted with mental health conditions. Early identification of mental health issues in each student and the provision of counseling services are essential steps^49^. Furthermore, encouraging physical activity and in-person social interactions among students, away from the digital confines of smartphones and SNS, is crucial^50^. In addition, there’s a need to promote the cultivation of social connections that were severed during the lockdowns imposed by the COVID–19 pandemic.

### Strengths and limitations

To our knowledge, this is the first study to investigate the association between household income and adolescents’ mental health indicators using a South Korean database. Moreover, our research is distinguished by its long-term and large population-based approach, encompassing data from multiple countries. This database enabled us to identify trends both before and during the COVID–19 pandemic. Consequently, we were able to provide a comprehensive understanding of the relationship between income and mental health, as well as how these trends were affected by the pandemic. Additionally, we identified several risk factors, including female sex, alcohol consumption, and smoking status, which further worsened the relationship between income and mental health.

However, our study also has several limitations. First, the KYRBS is conducted among school students, which introduces a bias by excluding non-school students who are homeschooled or have dropped out. Second, the data represents subjective responses rather than objective metrics, which is a limitation inherent to survey-based cohort studies. Adolescents may lack awareness of their household income, potentially introducing biases into their responses. In addition, due to the sensitivity surrounding mental health issues in Korean society, participants might have felt pressured to provide socially acceptable answers, potentially underreporting their symptoms, even though the survey was anonymous. This pressure, combined with the potential reluctance to discuss sensitive topics, could also exacerbate non-response bias, as certain participants might avoid or skip questions they find uncomfortable. Third, the KYRBS dataset utilized does not include a temporal component, preventing us from analyzing the causal relationship between depression and other variables. Finally, since this study is based entirely on pre-existing survey data with predefined questions, it is challenging to explore the specific reasons and mechanisms by which household income levels affect adolescents’ mental health.

Even though we have various limitations, this study still has advantages.

## Conclusion

This is the first study to investigate how household income levels affect adolescents’ mental health problems using a long-term, large nationwide survey-based cohort study. The findings indicate the potential association, suggesting that lower-income are more vulnerable to stress, sadness, suicidal ideation, and suicide attempts among Korean adolescents. Despite that various policies temporarily reduced mental health problems among adolescents, this trend surged with the onset of the COVID–19 pandemic. This is based on evidence that lockdown policies have impacted various aspects of the lives of lower-income adolescents, such as limited access to online education, reduced privacy, and increased feelings of isolation compared to their higher-income peers. As this study is based on survey data to identify associations, future research should investigate the mechanisms by which income influences negative mental health indicators. Furthermore, given that the mental health indicators assessed are related to other psychiatric conditions such as depression, this study provides crucial baseline data for developing policies aimed at the prevention and support of adolescents experiencing mental health challenges.

## Electronic supplementary material

Below is the link to the electronic supplementary material.

Supplementary Material 1

## Data Availability

The data are available on reasonable request. Study protocol, statistical code: available from DKY (email: yonkkang@gmail.com). Data set: available from the Korea Disease Control and Prevention Agency through a data use agreement.
