# Supplementary material for: National trends in adolescents’ mental health by income level in South Korea, pre– and post–COVID–19, 2006–2022
Source: Sci Rep. 2024 Oct 23;14:25021. doi: 10.1038/s41598-024-74073-5 (PMC11499596; doi:10.1038/s41598-024-74073-5)
Supplement: Supplementary file 1 — Supplementary Material 1 [file 41598_2024_74073_MOESM1_ESM.docx]

| Supplementary Material |
| --- |

**National trends in adolescents' mental health by income level in South Korea, pre– and post–COVID–19, 2006–2022**

**Running title:** Association between mental health and household income levels among adolescents

Jaehyeong Cho^1,2#^, Jaeyu Park^2,3#^, Hayeon Lee^2#^, Hyesu Jo^2,3^, Sooji Lee^2,4^, Hyeon Jin Kim^2,3^, Yejun Son^2,5^, Hyunjee Kim^2,5^, Selin Woo^2,4^, Seokjun Kim^2,4^, Jiseung Kang^6,7^, Damiano Pizzol^8,9^, Jiyoung Hwang^2,4*^, Lee Smith^10*^, Dong Keon Yon^2,3,4,5,11,12*^

^#^ The authors were contributed equally as co-first authors.

***** The authors were contributed equally as corresponding authors.

**Corresponding authors**

**Jiyoung Hwang**, PhD

Center for Digital Health, Medical Science Research Institute, Kyung Hee University College of Medicine, 23 Kyungheedae-ro, Dongdaemun-gu, Seoul, 02447, South Korea

Email: [cindy.jyhwang@gmail.com](mailto:cindy.jyhwang@gmail.com)

**Dong Keon Yon**, MD, PhD, FACAAI, FAAAAI (lead contact)

Department of Pediatrics, Kyung Hee University College of Medicine, 23 Kyungheedae-ro, Dongdaemun-gu, Seoul, 02447, South Korea

Tel: +82-2-6935-2476

Fax: +82-504-478-0201

Email: [yonkkang@gmail.com](mailto:yonkkang@gmail.com)

**Contents of supplementary appendix**

| Supplementary Material | Supplementary Material |
| --- | --- |
| Table S1 | Weighted baseline characteristics of adolescents reported in KYRBS, 2006–2022, weighted % (95% CI) |
| Table S2 | National Trends and Weighted Prevalence of Adolescent Mental Health Indicators by Household Income Level, KYRBS, 2006–2022, Weighted %, 95% CI |
| Table S3 | National trends and weighted prevalence of adolescents’ mental health indicators with various influencing factors, KYRBS, 2006–2022, weighted % (95% CI) |
| Table S4 | Comparative analysis of adolescents’ mental health indicators with various influencing factors: wOR approach based on KYRBS, 2006–2022 |
| Table S5 | Two-year comparative analysis of adolescents’ mental health indicators with various influencing factors, before and during the COVID–19 period: using wOR (95% CI) based on KYRBS, 2006–2022 |

**Table S1.** Weighted baseline characteristics of adolescents reported in KYRBS, 2006–2022, weighted % (95% CI)

| Variables | | Total | Before Pandemic | | | | | During Pandemic | | |
| --- | --- | --- | --- | --- | --- | --- | --- | --- | --- | --- |
|  |  |  | 2006-2008 | 2009-2011 | 2012-2014 | 2015-2017 | 2018-2019 | 2020 | 2021 | 2022 |
| Overall | | 100.00 | 15.42 (15.14-15.70) | 15.47 (15.20-15.74) | 14.45 (14.21-14.70) | 12.62 (12.40-12.84) | 10.95 (10.72-11.19) | 10.42 (10.11-10.73) | 10.41 (10.11-10.71) | 10.25 (9.93-10.57) |
| Age, mean (SD) | | 15.01 (1.75) | 15.00 (1.74) | 15.07 (1.74) | 14.91 (1.75) | 14.98 (1.74) | 14.99 (1.77) | 15.10 (1.75) | 15.09 (1.74) | 15.10 (1.73) |
| Sex | Male | 52.26 (51.58-52.94) | 53.05  (51.38-54.72) | 52.82 (51.15-54.49) | 52.33 (50.74-53.92) | 52.13 (50.51-53.75) | 52.02 (50.13-53.90) | 51.86 (49.38-54.34) | 51.67 (49.28-54.05) | 51.56 (49.20-53.92) |
|  | Female | 47.74 (47.06-48.42) | 46.95  (45.28-48.62) | 47.18 (45.51-48.85) | 47.67 (46.08-49.26) | 47.87 (46.25-49.49) | 47.98 (46.10-49.87) | 48.14 (45.66-50.62) | 48.33 (45.95-50.72) | 48.44 (46.08-50.80) |
| Grade level | Middle school  (7-9^th^) | 49.65 (49.24-50.06) | 52.78 (51.83-53.74) | 50.07 (49.13-51.01) | 48.70 (47.82-49.58) | 45.91 (45.03-46.80) | 47.13 (46.01-48.25) | 49.60 (48.00-51.19) | 50.96 (49.41-52.50) | 51.64 (49.98-53.30) |
|  | High school (10-12^th^) | 50.35 (49.94-50.76) | 47.22 (46.26-48.17)) | 49.93 (48.99-50.88) | 51.30 (50.42-52.18) | 54.09 (53.20-54.97) | 52.87 (51.75-53.99) | 50.40 (48.81-52.00) | 49.04 (47.50-50.59) | 48.36 (46.70-50.02) |
| Residential area | Rural | 30.79 (30.39-31.19) | 30.17 (29.29-31.04) | 30.56 (29.64-31.47) | 31.15 (30.24-32.06) | 31.10 (30.22-31.97) | 31.02 (29.90-32.13) | 30.90 (29.36-32.43) | 30.78 (29.34-32.23) | 30.84 (29.21-32.47) |
|  | Urban | 69.21 (68.81-69.61) | 69.83 (68.96-70.71) | 69.44 (68.53-70.36) | 68.85 (67.94-69.76) | 68.90 (68.03-69.78) | 68.98 (67.87-70.10) | 69.10 (67.57-70.64) | 69.22 (67.77-70.66) | 69.16 (67.53-70.79) |
| Recent alcohol consumption | No | 82.78 (82.63-82.94) | 73.21 (72.79-73.63) | 79.08 (78.70-79.46) | 82.51 (82.16-82.87) | 84.08 (83.74-84.42) | 84.03 (83.63-84.43) | 89.34 (88.89-89.78) | 89.27 (88.80-89.73) | 86.95 (86.42-87.48) |
|  | Yes | 17.22 (17.06-17.37) | 26.79 (26.37-27.21) | 20.92 (20.54-21.30) | 17.49 (17.13-17.84) | 15.92 (15.58-16.26) | 15.97 (15.57-16.37) | 10.66 (10.22-11.11) | 10.73 (10.27-11.20) | 13.05 (12.52-13.58) |
| Smoking status | No | 91.93 (91.80-92.05) | 87.06 (86.67-87.45) | 87.29 (86.90-87.68) | 88.93 (88.56-89.29) | 92.54 (92.24-92.85) | 94.39 (94.10-94.67) | 97.80 (97.61-97.98) | 96.85 (96.62-97.09) | 96.14 (95.86-96.42) |
|  | Yes | 8.07 (7.95-8.20) | 12.94 (12.55-13.33) | 12.71 (12.32-13.10) | 11.07 (10.71-11.44) | 7.46 (7.15-7.76) | 5.61 (5.33-5.90) | 2.21 (2.02-2.39) | 3.15 (2.91-3.38) | 3.86 (3.58-4.14) |
| Parental educational attainment | High school diploma or less | 48.35 (48.07-48.63) | 56.45 (55.68-57.21) | 50.44 (49.73-51.15) | 46.57 (45.94-47.20) | 40.82 (40.18-41.47) | 47.16 (46.42-47.89) | 49.45 (48.49-50.42) | 48.84 (47.87-49.82) | 44.41 (43.44-45.38) |
|  | Bachelor's degree or higher | 51.65 (51.37-51.93) | 43.55 (42.79-44.32) | 49.56 (48.85-50.27) | 53.43 (52.80-54.06) | 59.18 (58.53-59.82) | 52.84 (52.11-53.58) | 50.55 (49.58-51.51) | 51.16 (50.19-52.13) | 55.59 (54.62-56.56) |
| Academic achievement | Low | 37.00 (36.84-37.17) | 37.05 (36.73-37.37) | 35.25 (34.92-35.58) | 35.37 (35.04-35.70) | 38.28 (37.94-38.62) | 38.42 (38.00-38.84) | 36.86 (36.15-37.57) | 37.14 (36.49-37.79) | 38.80 (38.08-39.52) |
|  | Middle | 28.59 (28.47-28.70) | 26.86 (26.60-27.11) | 26.94 (26.69-27.19) | 27.61 (27.38-27.83) | 28.41 (28.17-28.65) | 29.74 (29.43-30.05) | 30.13 (29.68-30.58) | 31.01 (30.55-31.46) | 30.02 (29.55-30.49) |
|  | High | 34.41 (34.26-34.56) | 36.09 (35.79-36.40) | 37.81 (37.50-38.13) | 37.02 (36.71-37.34) | 33.31 (33.00-33.62) | 31.84 (31.46-32.22) | 33.01 (32.37-33.65) | 31.85 (31.27-32.44) | 31.18 (30.56-31.79) |

Abbreviations: CI, Confidence Interval; KYRBS, Korea Youth Risk Behavior Survey.

**Table S2.** National Trends and Weighted Prevalence of Adolescent Mental Health Indicators by Household Income Level, KYRBS, 2006–2022, Weighted %, 95% CI

| Variables | | Pre-pandemic | | | | | | | | | | | | | | Pandemic | | |
| --- | --- | --- | --- | --- | --- | --- | --- | --- | --- | --- | --- | --- | --- | --- | --- | --- | --- | --- |
|  |  | 2006 | 2007 | 2008 | 2009 | 2010 | 2011 | 2012 | 2013 | 2014 | 2015 | 2016 | 2017 | 2018 | 2019 | 2020 | 2021 | 2022 |
| Perceived stress level | High | 37.95 (36.28-39.61) | 40.71 (38.97-42.46) | 38.46 (36.59-40.34) | 40.06 (38.16-41.96) | 38.20 (36.27-40.12) | 36.10 (34.52-37.69) | 35.33 (33.75-36.91) | 34.81 (33.45-36.16) | 31.81 (30.37-33.25) | 30.35 (29.16-31.55) | 32.94 (31.63-34.25) | 32.54 (31.27-33.82) | 35.21 (33.87-36.55) | 34.40 (32.98-35.83) | 28.30 (26.83-29.76) | 36.63 (35.15-38.10) | 40.07 (38.67-41.48) |
|  | Mid-high | 41.97 (40.88-43.07) | 41.72 (40.72-42.72) | 39.06 (38.07-40.04) | 37.72 (36.67-38.77) | 38.48 (37.46-39.50) | 36.85 (35.97-37.72) | 36.82 (35.91-37.73) | 36.05 (35.17-36.94) | 32.47 (31.68-33.27) | 31.88 (31.06-32.70) | 34.39 (33.49-35.28) | 33.99 (33.09-34.90) | 37.31 (36.33-38.29) | 37.23 (36.21-38.25) | 31.75 (30.85-32.65) | 36.65 (35.72-37.59) | 38.82 (37.91-39.73) |
|  | Middle | 44.95 (44.10-45.80) | 44.29 (43.51-45.07) | 41.28 (40.46-42.11) | 41.53 (40.71-42.36) | 41.76 (40.96-42.57) | 40.48 (39.73-41.22) | 40.08 (39.35-40.81) | 40.43 (39.64-41.23) | 35.52 (34.85-36.20) | 34.18 (33.43-34.92) | 36.14 (35.32-36.95) | 36.40 (35.58-37.22) | 39.83 (38.95-40.70) | 39.47 (38.64-40.30) | 33.56 (32.79-34.33) | 37.78 (36.96-38.60) | 40.41 (39.61-41.21) |
|  | Mid-low | 55.61 (54.31-56.92) | 54.90 (53.69-56.11) | 51.30 (50.24-52.37) | 50.01 (48.81-51.22) | 52.44 (51.24-53.65) | 50.05 (48.95-51.15) | 50.75 (49.63-51.87) | 50.28 (49.12-51.44) | 47.17 (45.99-48.36) | 45.04 (43.81-46.28) | 47.47 (46.05-48.89) | 48.21 (46.78-49.64) | 52.50 (51.02-53.98) | 51.59 (50.06-53.11) | 45.82 (44.29-47.35) | 50.05 (48.50-51.60) | 52.10 (50.47-53.73) |
|  | Low | 64.07 (62.22-65.92) | 62.63 (60.83-64.44) | 63.47 (61.84-65.10) | 58.80 (56.91-60.69) | 61.91 (60.00-63.83) | 61.16 (59.27-63.04) | 59.94 (57.81-62.06) | 62.08 (60.17-63.98) | 58.38 (56.20-60.56) | 55.84 (53.39-58.28) | 56.03 (53.27-58.78) | 58.35 (55.62-61.08) | 60.74 (57.80-63.67) | 57.93 (54.65-61.20) | 52.85 (49.71-55.99) | 54.94 (51.63-58.25) | 62.77 (59.42-66.13) |
| Sadness | High | 36.21 (34.51-37.90) | 38.01 (36.23-39.78) | 37.87 (35.99-39.76) | 38.41 (36.35-40.47) | 35.50 (33.79-37.22) | 29.42 (27.96-30.88) | 28.29 (26.77-29.80) | 28.00 (26.71-29.30) | 24.74 (23.53-25.95) | 22.71 (21.62-23.80) | 23.81 (22.54-25.08) | 22.13 (21.04-23.23) | 24.57 (23.44-25.70) | 25.31 (24.15-26.47) | 22.32 (21.09-23.55) | 25.85 (24.59-27.11) | 28.15 (26.82-29.48) |
|  | Mid-high | 37.55 (36.56-38.54) | 38.32 (37.27-39.36) | 36.21 (35.17-37.26) | 34.31 (33.31-35.31) | 34.27 (33.29-35.26) | 29.95 (29.14-30.76) | 28.00 (27.18-28.81) | 27.55 (26.75-28.36) | 24.60 (23.86-25.34) | 21.62 (20.91-22.32) | 23.66 (22.87-24.46) | 24.05 (23.30-24.81) | 25.14 (24.34-25.94) | 26.77 (25.93-27.61) | 23.63 (22.84-24.42) | 25.28 (24.50-26.06) | 27.13 (26.32-27.94) |
|  | Middle | 39.96 (39.11-40.80) | 38.88 (38.19-39.57) | 36.37 (35.63-37.12) | 35.05 (34.27-35.84) | 34.66 (33.96-35.37) | 30.98 (30.37-31.59) | 28.28 (27.67-28.88) | 29.14 (28.44-29.84) | 24.77 (24.17-25.38) | 22.02 (21.42-22.61) | 24.07 (23.41-24.72) | 23.65 (22.98-24.32) | 26.06 (25.35-26.77) | 26.89 (26.14-27.63) | 24.29 (23.59-24.99) | 25.62 (24.93-26.32) | 27.49 (26.80-28.18) |
|  | Mid-low | 48.47 (47.18-49.75) | 47.24 (46.02-48.46) | 43.86 (42.65-45.07) | 42.14 (40.99-43.28) | 44.05 (42.83-45.26) | 38.18 (37.21-39.14) | 36.32 (35.33-37.31) | 37.82 (36.70-38.95) | 33.75 (32.67-34.83) | 29.11 (28.10-30.12) | 32.24 (31.07-33.41) | 31.83 (30.57-33.09) | 35.94 (34.54-37.34) | 36.96 (35.46-38.46) | 33.01 (31.64-34.37) | 35.21 (33.69-36.74) | 37.64 (36.07-39.20) |
|  | Low | 56.39 (54.51-58.27) | 57.21 (55.47-58.95) | 53.69 (51.83-55.54) | 52.34 (50.21-54.47) | 52.76 (50.91-54.62) | 49.09 (47.29-50.89) | 45.31 (43.50-47.11) | 47.72 (45.80-49.64) | 43.00 (40.87-45.13) | 41.39 (39.09-43.70) | 41.33 (38.93-43.73) | 45.48 (42.88-48.08) | 42.69 (39.79-45.58) | 47.44 (44.51-50.36) | 42.79 (39.85-45.74) | 44.83 (41.48-48.19) | 46.83 (43.32-50.34) |
| Suicidal ideation | High | 20.90 (19.47-22.33) | 23.03 (21.35-24.71) | 18.62 (17.21-20.04) | 19.21 (17.70-20.72) | 19.60 (18.05-21.16) | 19.11 (17.87-20.34) | 17.38 (16.03-18.73) | 16.35 (15.16-17.53) | 12.74 (11.76-13.71) | 11.00 (10.16-11.84) | 12.05 (11.11-13.00) | 11.09 (10.21-11.96) | 12.33 (11.37-13.29) | 11.25 (10.33-12.16) | 8.71 (7.89-9.52) | 10.87 (9.95-11.79) | 13.92 (12.87-14.97) |
|  | Mid-high | 20.07 (19.20-20.94) | 21.11 (20.31-21.92) | 16.87 (16.12-17.62) | 16.50 (15.74-17.27) | 16.88 (16.12-17.63) | 17.55 (16.87-18.22) | 15.85 (15.25-16.45) | 14.52 (13.91-15.14) | 11.67 (11.16-12.18) | 10.26 (9.74-10.78) | 10.59 (10.03-11.15) | 11.07 (10.52-11.61) | 11.47 (10.93-12.01) | 11.40 (10.81-11.99) | 9.81 (9.23-10.39) | 11.46 (10.89-12.04) | 12.58 (11.94-13.22) |
|  | Middle | 22.04 (21.31-22.77) | 21.46 (20.88-22.05) | 16.61 (16.03-17.20) | 16.99 (16.38-17.59) | 16.95 (16.32-17.58) | 17.49 (16.95-18.02) | 15.92 (15.39-16.45) | 15.01 (14.51-15.51) | 11.47 (11.06-11.88) | 10.23 (9.82-10.63) | 10.64 (10.20-11.08) | 10.90 (10.44-11.36) | 12.35 (11.87-12.83) | 12.12 (11.62-12.62) | 10.10 (9.63-10.57) | 11.90 (11.38-12.42) | 13.27 (12.76-13.78) |
|  | Mid-low | 28.76 (27.62-29.90) | 28.30 (27.27-29.32) | 22.94 (21.94-23.94) | 23.23 (22.28-24.17) | 24.07 (23.02-25.13) | 23.95 (23.05-24.85) | 23.71 (22.76-24.66) | 20.61 (19.76-21.46) | 17.57 (16.74-18.40) | 16.30 (15.46-17.13) | 17.77 (16.76-18.78) | 17.05 (16.04-18.06) | 20.47 (19.36-21.59) | 20.80 (19.54-22.07) | 16.94 (15.81-18.07) | 20.03 (18.74-21.33) | 22.25 (20.87-23.64) |
|  | Low | 37.90 (36.08-39.72) | 37.80 (35.96-39.63) | 33.17 (31.42-34.91) | 31.97 (30.06-33.87) | 33.24 (31.36-35.12) | 34.84 (33.15-36.53) | 33.81 (32.07-35.55) | 30.63 (28.89-32.38) | 26.16 (24.28-28.04) | 26.70 (24.64-28.76) | 26.09 (23.87-28.32) | 27.33 (24.81-29.84) | 27.62 (25.04-30.20) | 30.17 (27.23-33.11) | 24.21 (21.56-26.87) | 28.61 (25.68-31.55) | 31.70 (28.44-34.96) |
| Suicidal attempt | High | 7.62 (6.75-8.48) | 7.69 (6.68-8.70) | 5.95 (5.08-6.82) | 6.67 (5.70-7.63) | 6.50 (5.57-7.43) | 5.76 (5.02-6.50) | 5.17 (4.30-6.03) | 5.72 (5.02-6.43) | 4.09 (3.54-4.63) | 3.01 (2.57-3.46) | 3.67 (3.10-4.24) | 3.21 (2.75-3.68) | 3.39 (2.92-3.86) | 3.22 (2.77-3.67) | 1.90 (1.53-2.28) | 2.30 (1.85-2.76) | 3.42 (2.90-3.93) |
|  | Mid-high | 4.74 (4.34-5.14) | 4.83 (4.44-5.22) | 4.19 (3.76-4.62) | 3.79 (3.42-4.16) | 4.23 (3.82-4.63) | 3.43 (3.14-3.73) | 3.30 (3.00-3.61) | 3.41 (3.09-3.73) | 2.38 (2.12-2.64) | 2.07 (1.83-2.30) | 1.87 (1.65-2.09) | 2.19 (1.96-2.42) | 2.33 (2.08-2.58) | 2.32 (2.06-2.58) | 1.78 (1.52-2.03) | 1.80 (1.57-2.03) | 2.14 (1.87-2.41) |
|  | Middle | 4.60 (4.30-4.91) | 4.79 (4.49-5.08) | 3.68 (3.37-3.98) | 3.53 (3.28-3.77) | 3.89 (3.57-4.22) | 3.47 (3.25-3.70) | 3.31 (3.09-3.54) | 3.51 (3.26-3.75) | 2.34 (2.16-2.53) | 1.84 (1.67-2.01) | 1.83 (1.66-2.00) | 2.10 (1.91-2.29) | 2.69 (2.46-2.91) | 2.39 (2.19-2.60) | 1.69 (1.52-1.86) | 1.86 (1.68-2.04) | 2.26 (2.04-2.47) |
|  | Mid-low | 6.40 (5.71-7.08) | 6.91 (6.32-7.50) | 5.56 (5.09-6.04) | 5.44 (4.93-5.95) | 6.02 (5.42-6.62) | 4.89 (4.46-5.32) | 4.82 (4.40-5.23) | 4.64 (4.19-5.09) | 3.55 (3.16-3.95) | 3.26 (2.85-3.68) | 3.39 (2.92-3.86) | 3.40 (2.91-3.90) | 5.07 (4.49-5.64) | 5.37 (4.73-6.01) | 3.44 (2.93-3.95) | 4.02 (3.39-4.64) | 3.79 (3.18-4.41) |
|  | Low | 11.50 (10.09-12.91) | 12.81 (11.52-14.10) | 10.71 (9.69-11.74) | 10.65 (9.57-11.73) | 11.72 (10.40-13.04) | 11.59 (10.41-12.76) | 10.41 (9.26-11.56) | 9.96 (8.78-11.14) | 9.02 (7.84-10.20) | 8.87 (7.51-10.23) | 7.29 (6.11-8.46) | 9.24 (7.65-10.83) | 9.74 (7.91-11.57) | 10.58 (8.73-12.43) | 6.31 (4.79-7.82) | 8.23 (6.62-9.83) | 10.45 (8.46-12.45) |

Abbreviations: CI, Confidence Interval; KYRBS, the Korea Youth Risk Behavior Survey.

**Table S3.** National trends and weighted prevalence of adolescents’ mental health indicators with various influencing factors, KYRBS, 2006–2022, weighted % (95% CI)

| Variables | | | Pre-pandemic | | | | | Pandemic | | | Trend in the pre-pandemic era, β (95% CI) | Trend in the pandemic era, β (95% CI) | Trend difference, β_diff_ (95% CI) |
| --- | --- | --- | --- | --- | --- | --- | --- | --- | --- | --- | --- | --- | --- |
|  |  |  | 2006-2008 | 2009-2011 | 2012-2014 | 2015-2017 | 2018-2019 | 2020 | 2021 | 2022 |  |  |  |
| **Stress** | | | | | | | | | | | | | |
| Grade level | Middle school (7-9^th^) | High | 36.06 (34.82-37.29) | 35.45 (34.15-36.74) | 30.78 (29.74-31.81) | 29.02 (28.11-29.94) | 31.68 (30.45-32.90) | 24.84 (23.11-26.57) | 34.76 (32.81-36.71) | 37.64 (35.91-39.38) | **-1.51 (-1.89 to -1.13)** | **2.83 (2.13 to 3.53)** | **4.34 (3.54 to 5.14)** |
|  |  | Mid-high | 37.89 (37.15-38.64) | 34.78 (34.07-35.49) | 32.17 (31.55-32.79) | 29.97 (29.35-30.60) | 34.15 (33.24-35.06) | 27.87 (26.78-28.97) | 34.11 (32.91-35.32) | 37.01 (35.80-38.22) | **-1.33 (-1.58 to -1.08)** | **1.50 (1.02 to 1.99)** | **2.83 (2.28 to 3.38)** |
|  |  | Middle | 40.51 (39.89-41.14) | 38.86 (38.26-39.46) | 36.85 (36.28-37.42) | 32.64 (32.11-33.17) | 37.53 (36.75-38.31) | 30.19 (29.23-31.15) | 35.70 (34.67-36.72) | 39.38 (38.26-40.50) | **-1.35 (-1.56 to -1.13)** | **1.09 (0.66 to 1.53)** | **2.44 (1.95 to 2.93)** |
|  |  | Mid-low | 52.94 (51.98-53.90) | 49.49 (48.53-50.45) | 50.03 (49.04-51.01) | 47.24 (46.09-48.40) | 52.01 (50.39-53.63) | 47.11 (44.87-49.36) | 50.99 (48.76-53.22) | 53.94 (51.57-56.32) | **-0.71 (-1.09 to -0.32)** | **0.96 (0.04 to 1.88)** | **1.67 (0.67 to 2.67)** |
|  |  | low | 61.55 (59.98-63.13) | 58.91 (57.26-60.56) | 59.43 (57.61-61.25) | 54.62 (52.09-57.15) | 59.52 (56.01-63.04) | 49.58 (44.41-54.76) | 55.97 (50.57-61.37) | 63.12 (57.93-68.31) | **-1.10 (-1.83 to -0.37)** | 1.79 (-0.24 to 3.83) | 2.89 (0.73 to 5.05) |
|  | High school (10-12^th^) | High | 44.98 (43.15-46.81) | 42.52 (40.77-44.27) | 39.45 (38.01-40.89) | 36.31 (35.11-37.52) | 39.27 (37.69-40.86) | 34.15 (31.70-36.59) | 39.65 (37.40-41.90) | 44.20 (41.83-46.57) | **-1.68 (-2.20 to -1.15)** | **1.92 (1.02 to 2.83)** | **3.60 (2.55 to 4.65)** |
|  |  | Mid-high | 45.56 (44.60-46.52) | 41.64 (40.74-42.55) | 38.91 (38.09-39.72) | 37.17 (36.38-37.97) | 40.66 (39.58-41.74) | 36.34 (34.90-37.78) | 39.83 (38.39-41.27) | 41.11 (39.73-42.48) | **-1.41 (-1.72 to -1.10)** | 0.43 (-0.13 to 0.99) | **1.84 (1.20 to 2.48)** |
|  |  | Middle | 46.74 (46.02-47.45) | 43.61 (42.92-44.30) | 40.40 (39.77-41.02) | 37.78 (37.08-38.48) | 41.36 (40.48-42.25) | 36.52 (35.36-37.67) | 39.75 (38.48-41.01) | 41.35 (40.22-42.49) | **-1.76 (-2.00 to -1.51)** | 0.25 (-0.21 to 0.71) | **2.01 (1.49 to 2.53)** |
|  |  | Mid-low | 54.62 (53.65-55.59) | 51.76 (50.83-52.70) | 49.29 (48.40-50.17) | 46.53 (45.51-47.55) | 52.09 (50.71-53.48) | 44.98 (42.94-47.03) | 49.44 (47.33-51.55) | 50.69 (48.48-52.91) | **-1.39 (-1.74 to -1.04)** | -0.19 (-1.01 to 0.63) | **1.20 (0.31 to 2.09)** |
|  |  | low | 64.66 (63.34-65.98) | 61.57 (60.09-63.04) | 60.62 (59.06-62.17) | 57.37 (55.50-59.25) | 59.41 (56.66-62.16) | 54.51 (50.57-58.45) | 54.39 (50.21-58.57) | 62.52 (58.12-66.92) | **-1.68 (-2.27 to -1.10)** | 0.54 (-1.07 to 2.15) | **2.22 (0.51 to 3.93)** |
| Residential area | Rural | High | 36.97 (35.35-38.58) | 38.21 (36.20-40.22) | 34.58 (32.97-36.20) | 31.75 (30.36-33.13) | 33.38 (31.53-35.24) | 28.19 (25.97-30.40) | 33.97 (31.59-36.36) | 39.20 (36.63-41.76) | **-1.43 (-1.80 to -1.06)** | **2.41 (1.75 to 3.07)** | **3.84 (3.08 to 4.60)** |
|  |  | Mid-high | 39.38 (38.45-40.31) | 37.07 (36.11-38.03) | 34.38 (33.37-35.39) | 33.21 (32.27-34.15) | 36.49 (35.16-37.81) | 30.64 (28.93-32.34) | 35.42 (33.74-37.11) | 36.91 (35.24-38.58) | **-1.29 (-1.53 to -1.05)** | **1.07 (0.63 to 1.51)** | **2.36 (1.86 to 2.86)** |
|  |  | Middle | 42.29 (41.54-43.03) | 40.94 (40.11-41.76) | 38.45 (37.67-39.23) | 35.37 (34.59-36.15) | 38.90 (37.87-39.94) | 32.72 (31.36-34.07) | 36.75 (35.31-38.20) | 38.55 (37.17-39.92) | **-1.52 (-1.73 to -1.32)** | **0.76 (0.37 to 1.16)** | **2.28 (1.83 to 2.73)** |
|  |  | Mid-low | 53.82 (52.72-54.92) | 51.70 (50.58-52.82) | 49.32 (48.09-50.55) | 47.18 (45.84-48.53) | 50.13 (48.29-51.97) | 45.58 (43.12-48.04) | 50.41 (47.67-53.15) | 49.69 (47.09-52.28) | **-0.97 (-1.30 to -0.65)** | 0.32 (-0.45 to 1.09) | **1.29 (0.45 to 2.13)** |
|  |  | low | 62.68 (61.12-64.24) | 59.93 (58.31-61.55) | 58.81 (56.65-60.96) | 57.02 (54.37-59.66) | 57.50 (53.41-61.59) | 54.76 (49.47-60.04) | 56.70 (51.54-61.86) | 59.67 (54.24-65.09) | **-1.35 (-1.92 to -0.79)** | 1.10 (-0.46 to 2.66) | **2.45 (0.79 to 4.11)** |
|  | Urban | High | 39.69 (38.43-40.96) | 38.05 (36.81-39.28) | 33.68 (32.69-34.66) | 32.04 (31.18-32.90) | 35.34 (34.19-36.49) | 28.34 (26.50-30.18) | 37.69 (35.88-39.51) | 40.41 (38.74-42.07) | **-1.36 (-1.91 to -0.81)** | **2.34 (1.34 to 3.34)** | **3.70 (2.56 to 4.84)** |
|  |  | Mid-high | 41.52 (40.78-42.25) | 37.92 (37.23-38.62) | 35.42 (34.85-35.98) | 33.48 (32.88-34.08) | 37.58 (36.75-38.42) | 32.20 (31.14-33.25) | 37.13 (36.01-38.25) | 39.58 (38.51-40.65) | **-1.01 (-1.35 to -0.66)** | 0.58 (-0.10 to 1.26) | **1.59 (0.83 to 2.35)** |
|  |  | Middle | 44.03 (43.44-44.63) | 41.41 (40.86-41.96) | 38.82 (38.32-39.33) | 35.60 (35.04-36.17) | 40.02 (39.27-40.76) | 33.97 (33.04-34.90) | 38.26 (37.27-39.26) | 41.31 (40.33-42.30) | **-1.32 (-1.59 to -1.04)** | 0.24 (-0.31 to 0.79) | **1.56 (0.95 to 2.17)** |
|  |  | Mid-low | 53.94 (53.07-54.81) | 50.45 (49.61-51.30) | 49.67 (48.87-50.46) | 46.56 (45.60-47.52) | 53.04 (51.74-54.33) | 45.94 (44.01-47.86) | 49.86 (47.98-51.74) | 53.33 (51.27-55.39) | **-1.41 (-1.84 to -0.97)** | 0.28 (-0.74 to 1.29) | **1.69 (0.59 to 2.79)** |
|  |  | low | 63.74 (62.43-65.04) | 60.81 (59.38-62.25) | 60.91 (59.47-62.35) | 56.39 (54.53-58.25) | 60.43 (57.85-63.00) | 51.86 (47.95-55.76) | 53.93 (49.64-58.22) | 64.24 (60.04-68.45) | **-1.51 (-2.31 to -0.71)** | 0.73 (-1.42 to 2.87) | 2.24 (-0.05 to 4.53) |
| Recent alcohol consumption | No | High | 36.37 (35.18-37.55) | 35.90 (34.76-37.03) | 32.09 (31.18-33.00) | 30.56 (29.78-31.34) | 32.66 (31.63-33.70) | 27.15 (25.64-28.66) | 35.61 (34.02-37.21) | 38.94 (37.48-40.41) | **-1.24 (-1.58 to -0.90)** | **2.77 (2.18 to 3.35)** | **4.01 (3.33 to 4.69)** |
|  |  | Mid-high | 38.81 (38.12-39.49) | 36.12 (35.50-36.74) | 33.49 (32.96-34.02) | 32.13 (31.60-32.66) | 35.94 (35.21-36.68) | 30.74 (29.81-31.67) | 35.97 (35.01-36.93) | 38.09 (37.14-39.05) | **-0.99 (-1.20 to -0.77)** | **1.17 (0.78 to 1.56)** | **2.16 (1.71 to 2.61)** |
|  |  | Middle | 41.50 (40.98-42.02) | 39.88 (39.38-40.37) | 37.33 (36.88-37.77) | 34.53 (34.06-34.99) | 38.45 (37.83-39.07) | 32.39 (31.61-33.17) | 37.06 (36.22-37.89) | 39.42 (38.59-40.25) | **-1.23 (-1.41 to -1.06)** | **0.74 (0.41 to 1.07)** | **1.97 (1.60 to 2.34)** |
|  |  | Mid-low | 51.66 (50.89-52.43) | 49.12 (48.35-49.88) | 48.32 (47.60-49.04) | 45.65 (44.80-46.49) | 50.84 (49.68-52.01) | 44.42 (42.80-46.04) | 48.92 (47.27-50.57) | 51.55 (49.81-53.30) | **-0.78 (-1.07 to -0.49)** | 0.56 (-0.10 to 1.23) | **1.34 (0.61 to 2.07)** |
|  |  | low | 60.68 (59.36-62.01) | 58.79 (57.49-60.09) | 58.28 (56.87-59.69) | 55.07 (53.33-56.81) | 57.50 (54.91-60.09) | 50.58 (47.17-53.99) | 53.09 (49.40-56.77) | 60.90 (56.97-64.82) | **-1.17 (-1.73 to -0.60)** | 1.11 (-0.37 to 2.60) | **2.28 (0.69 to 3.87)** |
|  | Yes | High | 46.77 (44.81-48.73) | 46.22 (44.01-48.44) | 43.16 (41.10-45.21) | 39.76 (37.70-41.83) | 45.55 (43.19-47.90) | 38.42 (34.24-42.60) | 45.45 (41.08-49.83) | 47.55 (43.38-51.71) | **-0.90 (-1.58 to -0.22)** | 0.97 (-0.54 to 2.48) | **1.87 (0.21 to 3.53)** |
|  |  | Mid-high | 47.69 (46.53-48.84) | 44.55 (43.33-45.77) | 44.34 (43.08-45.60) | 41.24 (39.92-42.55) | 45.35 (43.67-47.03) | 41.28 (38.50-44.06) | 43.16 (40.50-45.82) | 44.10 (41.55-46.65) | **-0.96 (-1.40 to -0.53)** | -0.3 (-1.27 to 0.67) | 0.66 (-0.40 to 1.72) |
|  |  | Middle | 49.28 (48.43-50.13) | 47.02 (46.11-47.94) | 45.72 (44.81-46.64) | 41.06 (40.01-42.11) | 46.27 (44.94-47.60) | 43.92 (41.75-46.10) | 44.03 (41.75-46.31) | 47.33 (45.21-49.46) | **-1.39 (-1.73 to -1.06)** | 0.22 (-0.57 to 1.01) | **1.61 (0.75 to 2.47)** |
|  |  | Mid-low | 58.89 (57.59-60.18) | 56.18 (54.92-57.44) | 54.06 (52.76-55.36) | 51.16 (49.52-52.80) | 56.82 (54.55-59.09) | 54.02 (50.02-58.02) | 56.35 (52.37-60.33) | 54.76 (50.72-58.81) | **-1.38 (-1.91 to -0.86)** | -0.48 (-1.92 to 0.96) | 0.90 (-0.63 to 2.43) |
|  |  | low | 67.89 (66.34-69.44) | 64.21 (62.42-65.99) | 64.76 (62.56-66.97) | 60.67 (57.88-63.47) | 64.35 (60.68-68.02) | 62.50 (55.78-69.21) | 61.95 (55.13-68.76) | 68.78 (62.28-75.29) | **-1.39 (-2.15 to -0.62)** | 1.08 (-1.26 to 3.42) | **2.47 (0.01 to 4.93)** |
| Smoking status | No | High | 37.22 (36.12-38.33) | 36.28 (35.21-37.35) | 32.68 (31.79-33.56) | 31.16 (30.40-31.92) | 33.93 (32.92-34.93) | 27.62 (26.15-29.10) | 36.27 (34.75-37.78) | 39.36 (37.97-40.74) | **-1.12 (-1.45 to -0.80)** | **2.50 (1.95 to 3.06)** | **3.62 (2.98 to 4.26)** |
|  |  | Mid-high | 39.96 (39.33-40.60) | 36.81 (36.21-37.40) | 34.30 (33.77-34.83) | 33.00 (32.48-33.52) | 36.99 (36.27-37.71) | 31.55 (30.65-32.44) | 36.32 (35.38-37.26) | 38.55 (37.62-39.47) | **-1.00 (-1.21 to -0.80)** | **0.94 (0.56 to 1.31)** | **1.94 (1.51 to 2.37)** |
|  |  | Middle | 42.42 (41.92-42.93) | 40.54 (40.06-41.02) | 37.95 (37.51-38.40) | 35.19 (34.72-35.66) | 39.40 (38.79-40.01) | 33.43 (32.65-34.21) | 37.56 (36.73-38.39) | 40.08 (39.27-40.89) | **-1.24 (-1.41 to -1.07)** | **0.58 (0.26 to 0.91)** | **1.82 (1.45 to 2.19)** |
|  |  | Mid-low | 52.82 (52.09-53.54) | 49.95 (49.24-50.67) | 48.99 (48.30-49.69) | 46.42 (45.60-47.25) | 52.02 (50.93-53.11) | 45.63 (44.10-47.16) | 49.53 (47.95-51.12) | 51.94 (50.28-53.60) | **-0.82 (-1.09 to -0.55)** | 0.26 (-0.37 to 0.89) | **1.08 (0.39 to 1.77)** |
|  |  | low | 61.70 (60.50-62.91) | 59.10 (57.86-60.34) | 59.06 (57.70-60.41) | 56.66 (54.98-58.35) | 59.22 (56.86-61.58) | 51.90 (48.69-55.12) | 54.55 (51.13-57.97) | 61.90 (58.17-65.64) | **-0.94 (-1.47 to -0.42)** | 0.87 (-0.51 to 2.25) | **1.81 (0.33 to 3.29)** |
|  | Yes | High | 50.20 (47.54-52.87) | 48.20 (45.55-50.85) | 43.42 (40.97-45.86) | 40.18 (37.52-42.84) | 46.27 (42.77-49.76) | 52.22 (43.59-60.86) | 45.06 (37.62-52.49) | 51.71 (44.79-58.64) | **-1.85 (-2.79 to -0.92)** | 1.32 (-1.14 to 3.77) | **3.17 (0.54 to 5.80)** |
|  |  | Mid-high | 49.03 (47.24-50.81) | 45.21 (43.53-46.89) | 43.53 (41.95-45.10) | 39.56 (37.63-41.49) | 43.04 (40.15-45.92) | 41.57 (35.14-48.00) | 48.73 (43.10-54.36) | 46.90 (42.06-51.73) | **-2.07 (-2.75 to -1.39)** | 1.66 (-0.12 to 3.44) | **3.73 (1.82 to 5.64)** |
|  |  | Middle | 51.63 (50.35-52.91) | 47.04 (45.86-48.22) | 45.44 (44.27-46.61) | 40.39 (38.92-41.87) | 44.43 (42.03-46.82) | 40.51 (36.04-44.99) | 45.68 (41.49-49.88) | 50.25 (46.43-54.08) | **-2.51 (-3.03 to -2.00)** | **1.84 (0.43 to 3.25)** | **4.35 (2.85 to 5.85)** |
|  |  | Mid-low | 59.84 (58.10-61.58) | 55.83 (54.28-57.39) | 53.10 (51.49-54.71) | 49.90 (47.68-52.13) | 52.64 (48.99-56.29) | 52.75 (43.80-61.70) | 60.37 (53.36-67.39) | 55.19 (47.86-62.52) | **-2.57 (-3.30 to -1.83)** | 1.56 (-0.91 to 4.02) | **4.13 (1.56 to 6.70)** |
|  |  | low | 68.39 (66.43-70.35) | 64.85 (62.70-67.01) | 63.89 (61.47-66.31) | 56.35 (53.20-59.50) | 60.73 (55.32-66.14) | 67.41 (55.82-79.01) | 58.93 (47.41-70.44) | 68.64 (59.82-77.46) | **-2.73 (-3.71 to -1.76)** | 1.93 (-1.37 to 5.23) | **4.66 (1.22 to 8.10)** |
| Parental educational attainment | High school diploma or less | High | 38.39 (36.81-39.98) | 39.98 (38.04-41.93) | 34.96 (33.34-36.58) | 31.59 (30.18-32.99) | 34.28 (32.82-35.73) | 27.88 (25.81-29.94) | 36.25 (34.22-38.28) | 40.26 (38.16-42.37) | **-1.51 (-2.00 to -1.01)** | **2.64 (1.82 to 3.46)** | **4.15 (3.19 to 5.11)** |
|  |  | Mid-high | 39.44 (38.57-40.31) | 36.94 (36.01-37.87) | 34.81 (33.99-35.64) | 32.48 (31.65-33.31) | 36.95 (35.88-38.01) | 31.72 (30.41-33.04) | 36.45 (35.02-37.88) | 37.30 (35.85-38.75) | **-0.94 (-1.24 to -0.64)** | **0.58 (0.01 to 1.15)** | 1.52 (0.88 to 2.16) |
|  |  | Middle | 42.44 (41.87-43.00) | 40.64 (40.05-41.22) | 38.43 (37.89-38.98) | 34.78 (34.17-35.38) | 38.88 (38.13-39.64) | 33.59 (32.62-34.57) | 37.07 (36.00-38.13) | 40.49 (39.42-41.55) | **-1.38 (-1.59 to -1.18)** | **0.75 (0.33 to 1.16)** | **2.13 (1.67 to 2.59)** |
|  |  | Mid-low | 53.31 (52.54-54.08) | 50.38 (49.54-51.22) | 49.12 (48.29-49.95) | 46.17 (45.17-47.17) | 52.00 (50.67-53.33) | 45.05 (43.16-46.94) | 49.94 (47.95-51.93) | 51.65 (49.50-53.81) | **-1.06 (-1.37 to -0.74)** | 0.23 (-0.56 to 1.02) | **1.29 (0.44 to 2.14)** |
|  |  | low | 63.02 (61.90-64.14) | 60.04 (58.82-61.26) | 59.69 (58.30-61.09) | 56.67 (54.86-58.48) | 58.92 (56.34-61.50) | 51.63 (47.89-55.36) | 52.14 (48.28-56.00) | 62.84 (58.97-66.70) | **-1.40 (-1.93 to -0.86)** | 0.96 (-0.52 to 2.44) | **2.36 (0.79 to 3.93)** |
|  | Bachelor's degree or higher | High | 39.26 (37.91-40.61) | 37.31 (36.09-38.53) | 33.53 (32.55-34.50) | 32.09 (31.26-32.93) | 35.14 (33.93-36.35) | 28.58 (26.76-30.39) | 36.89 (34.85-38.93) | 39.96 (38.21-41.72) | **-1.35 (-1.74 to -0.96)** | **2.24 (1.55 to 2.93)** | **3.59 (2.80 to 4.38)** |
|  |  | Mid-high | 41.89 (41.12-42.66) | 38.04 (37.37-38.72) | 35.25 (34.66-35.83) | 33.76 (33.17-34.34) | 37.46 (36.62-38.30) | 31.77 (30.65-32.88) | 36.78 (35.62-37.94) | 39.65 (38.55-40.75) | **-1.38 (-1.62 to -1.14)** | **1.12 (0.68 to 1.57)** | **2.50 (1.99 to 3.01)** |
|  |  | Middle | 45.03 (44.34-45.72) | 41.98 (41.36-42.59) | 38.97 (38.43-39.51) | 36.15 (35.61-36.70) | 40.46 (39.68-41.25) | 33.53 (32.49-34.58) | 38.59 (37.52-39.66) | 40.34 (39.24-41.43) | **-1.63 (-1.85 to -1.40)** | 0.42 (-0.01 to 0.85) | **2.05 (1.56 to 2.54)** |
|  |  | Mid-low | 55.40 (54.10-56.69) | 51.71 (50.66-52.76) | 50.30 (49.33-51.27) | 47.59 (46.46-48.72) | 52.16 (50.51-53.81) | 47.08 (44.66-49.50) | 50.22 (47.82-52.62) | 52.75 (50.27-55.22) | **-1.32 (-1.75 to -0.89)** | 0.39 (-0.55 to 1.34) | **1.71 (0.67 to 2.75)** |
|  |  | low | 64.64 (62.37-66.91) | 61.86 (59.80-63.93) | 61.54 (59.53-63.54) | 56.45 (53.84-59.07) | 60.65 (56.87-64.44) | 55.87 (50.23-61.50) | 63.39 (57.00-69.77) | 62.61 (56.48-68.74) | **-1.56 (-2.44 to -0.68)** | 1.18 (-1.09 to 3.46) | **2.74 (0.30 to 5.18)** |
| Academic achievement | Low | High | 39.05 (37.71-40.38) | 36.70 (35.47-37.94) | 32.41 (31.36-33.45) | 31.15 (30.25-32.06) | 34.09 (32.83-35.34) | 27.43 (25.71-29.16) | 36.44 (34.47-38.41) | 38.90 (37.18-40.62) | **-1.51 (-1.90 to -1.11)** | **2.31 (1.62 to 3.00)** | **3.82 (3.02 to 4.62)** |
|  |  | Mid-high | 39.53 (38.73-40.33) | 35.82 (35.06-36.58) | 32.35 (31.69-33.00) | 31.46 (30.82-32.09) | 35.46 (34.57-36.35) | 29.81 (28.60-31.02) | 35.06 (33.84-36.27) | 37.88 (36.61-39.15) | **-1.33 (-1.59 to -1.07)** | **1.22 (0.73 to 1.72)** | **2.55 (1.99 to 3.11)** |
|  |  | Middle | 42.02 (41.29-42.75) | 38.80 (38.07-39.54) | 36.19 (35.54-36.84) | 33.94 (33.28-34.60) | 38.12 (37.22-39.01) | 31.77 (30.55-32.99) | 37.01 (35.71-38.30) | 39.51 (38.15-40.87) | **-1.45 (-1.69 to -1.20)** | **0.84 (0.33 to 1.35)** | **2.29 (1.72 to 2.86)** |
|  |  | Mid-low | 52.57 (51.24-53.91) | 47.25 (46.06-48.44) | 46.58 (45.36-47.79) | 44.92 (43.52-46.31) | 49.61 (47.55-51.67) | 42.26 (39.20-45.32) | 49.20 (45.70-52.69) | 50.25 (46.90-53.61) | **-1.28 (-1.78 to -0.78)** | 0.69 (-0.55 to 1.94) | **1.97 (0.63 to 3.31)** |
|  |  | low | 60.32 (57.95-62.69) | 57.73 (55.12-60.34) | 56.02 (53.38-58.66) | 53.65 (50.51-56.78) | 55.84 (51.44-60.23) | 47.71 (40.69-54.72) | 56.12 (49.09-63.15) | 57.13 (49.00-65.26) | **-1.56 (-2.56 to -0.57)** | 0.86 (-1.95 to 3.66) | 2.42 (-0.56 to 5.40) |
|  | Middle | High | 36.92 (34.61-39.23) | 35.86 (33.28-38.43) | 32.22 (30.21-34.24) | 28.92 (27.26-30.58) | 32.31 (30.33-34.29) | 25.35 (22.77-27.93) | 34.69 (31.65-37.73) | 39.49 (36.29-42.68) | **-1.53 (-2.21 to -0.85)** | **3.05 (1.85 to 4.24)** | **4.58  (3.21 to 5.95)** |
|  |  | Mid-high | 39.78 (38.63-40.93) | 35.88 (34.83-36.93) | 34.40 (33.52-35.29) | 32.68 (31.80-33.56) | 36.91 (35.76-38.05) | 32.05 (30.51-33.58) | 35.99 (34.45-37.52) | 37.25 (35.71-38.79) | **-0.90 (-1.25 to -0.55)** | 0.48 (-0.14 to 1.09) | **1.38  (0.67 to 2.09)** |
|  |  | Middle | 41.25 (40.51-41.99) | 39.42 (38.71-40.13) | 36.43 (35.79-37.08) | 33.12 (32.45-33.78) | 37.45 (36.60-38.30) | 31.04 (29.79-32.30) | 35.77 (34.58-36.96) | 38.09 (36.88-39.31) | **-1.47 (-1.72 to -1.23)** | 0.62 (0.15 to 1.10) | **2.09  (1.56 to 2.62)** |
|  |  | Mid-low | 51.91 (50.67-53.14) | 50.12 (48.88-51.37) | 46.98 (45.78-48.18) | 44.89 (43.44-46.33) | 50.80 (48.86-52.74) | 44.06 (41.21-46.90) | 47.94 (45.03-50.86) | 48.66 (45.39-51.94) | **-1.12 (-1.59 to -0.64)** | -0.34 (-1.53 to 0.86) | 0.78  (-0.51 to 2.07) |
|  |  | low | 64.22 (61.68-66.75) | 59.00 (56.21-61.78) | 58.45 (55.51-61.39) | 55.74 (52.05-59.42) | 56.32 (51.17-61.47) | 48.11 (40.68-55.54) | 48.46 (40.04-56.87) | 62.54 (54.07-71.02) | **-2.19 (-3.31 to -1.07)** | 1.34 (-1.85 to 4.54) | **3.53  (0.14 to 6.92)** |
|  | High | High | 40.14 (37.91-42.37) | 44.53 (42.24-46.82) | 39.76 (37.95-41.57) | 36.99 (35.35-38.63) | 39.65 (37.47-41.83) | 33.61 (30.57-36.66) | 39.17 (36.11-42.23) | 44.78 (41.56-48.01) | **-0.82 (-1.50 to -0.13)** | **2.09 (0.84 to 3.34)** | **2.91  (1.48 to 4.34)** |
|  |  | Mid-high | 45.49 (44.31-46.68) | 43.59 (42.51-44.67) | 41.83 (40.84-42.82) | 38.76 (37.78-39.74) | 42.06 (40.76-43.35) | 35.68 (33.77-37.58) | 41.60 (39.57-43.63) | 43.24 (41.38-45.10) | **-1.23 (-1.61 to -0.85)** | **0.93 (0.19 to 1.66)** | **2.16  (1.33 to 2.99)** |
|  |  | Middle | 46.73 (46.01-47.44) | 44.64 (43.99-45.28) | 42.44 (41.83-43.04) | 39.07 (38.39-39.75) | 43.07 (42.15-43.98) | 37.30 (36.13-38.46) | 40.35 (39.11-41.59) | 43.39 (42.14-44.64) | **-1.41 (-1.66 to -1.16)** | 0.36 (-0.13 to 0.86) | **1.77  (1.22 to 2.32)** |
|  |  | Mid-low | 55.61 (54.65-56.57) | 52.92 (51.99-53.85) | 52.17 (51.30-53.04) | 48.59 (47.55-49.63) | 53.85 (52.49-55.20) | 48.30 (46.31-50.29) | 51.39 (49.19-53.59) | 54.72 (52.51-56.93) | **-1.06 (-1.41 to -0.71)** | 0.42 (-0.40 to 1.24) | **1.48  (0.59 to 2.37)** |
|  |  | low | 64.13 (62.86-65.41) | 61.59 (60.29-62.89) | 61.77 (60.37-63.18) | 57.85 (55.93-59.77) | 61.53 (58.81-64.24) | 55.43 (51.61-59.25) | 56.28 (52.26-60.30) | 64.26 (60.25-68.26) | **-1.12 (-1.69 to -0.55)** | 0.80  (-0.75 to 2.34) | **1.92  (1.35 to 2.49)** |
| **Sadness** | | | | | | | | | | | | | |
| Grade level | Middle school (7-9^th^) | High | 34.82 (33.60-36.05) | 31.23 (29.93-32.53) | 24.40 (23.46-25.34) | 20.59 (19.80-21.39) | 23.08 (22.09-24.06) | 19.39 (17.98-20.80) | 25.48 (23.98-26.99) | 26.43 (24.80-28.06) | **-3.42 (-3.77 to -3.06)** | **1.63 (1.02 to 2.25)** | **5.05 (4.34 to 5.76)** |
|  |  | Mid-high | 34.32 (33.57-35.08) | 29.75 (29.06-30.43) | 24.40 (23.82-24.97) | 20.72 (20.17-21.27) | 24.01 (23.24-24.78) | 21.25 (20.22-22.28) | 24.54 (23.55-25.53) | 26.27 (25.18-27.36) | **-3.09 (-3.32 to -2.85)** | **1.02 (0.59 to 1.45)** | **4.11 (3.62 to 4.60)** |
|  |  | Middle | 35.11 (34.56-35.67) | 31.20 (30.65-31.75) | 26.10 (25.59-26.61) | 21.62 (21.13-22.11) | 25.64 (24.92-26.35) | 22.70 (21.84-23.55) | 24.84 (23.89-25.79) | 27.34 (26.35-28.33) | **-3.05 (-3.25 to -2.86)** | **0.72 (0.33 to 1.10)** | **3.77 (3.34 to 4.20)** |
|  |  | Mid-low | 45.04 (44.01-46.07) | 40.33 (39.36-41.30) | 36.37 (35.43-37.31) | 31.37 (30.32-32.43) | 37.50 (35.90-39.11) | 33.57 (31.47-35.68) | 36.70 (34.34-39.06) | 40.68 (38.31-43.05) | **-2.90 (-3.29 to -2.51)** | **1.26 (0.34 to 2.18)** | **4.16 (3.16 to 5.16)** |
|  |  | low | 53.56 (51.90-55.23) | 50.28 (48.58-51.99) | 45.85 (43.88-47.83) | 41.32 (38.90-43.74) | 46.41 (42.78-50.03) | 40.69 (35.48-45.89) | 46.84 (41.61-52.08) | 48.09 (42.52-53.65) | **-2.88 (-3.63 to -2.12)** | 1.13 (-1.01 to 3.27) | **4.01 (1.74 to 6.28)** |
|  | High school (10-12^th^) | High | 42.42 (40.52-44.32) | 39.74 (38.03-41.44) | 31.44 (30.07-32.80) | 26.23 (25.07-27.38) | 27.57 (26.21-28.94) | 27.28 (25.15-29.41) | 26.44 (24.22-28.66) | 31.07 (28.83-33.31) | **-4.24 (-4.74 to -3.73)** | **0.94 (0.11 to 1.78)** | **5.18 (4.20 to 6.16)** |
|  |  | Mid-high | 42.01 (41.08-42.95) | 37.01 (36.12-37.89) | 29.70 (28.96-30.44) | 25.71 (25.03-26.38) | 27.98 (27.11-28.85) | 26.45 (25.23-27.67) | 26.20 (24.96-27.44) | 28.23 (27.02-29.44) | **-3.93 (-4.21 to -3.65)** | 0.03 (-0.44 to 0.51) | **3.96 (3.41 to 4.51)** |
|  |  | Middle | 41.95 (41.28-42.62) | 35.92 (35.32-36.52) | 28.62 (28.09-29.16) | 24.46 (23.92-24.99) | 27.14 (26.41-27.87) | 25.68 (24.61-26.75) | 26.37 (25.35-27.38) | 27.62 (26.66-28.59) | **-4.25 (-4.46 to -4.04)** | 0.18 (-0.20 to 0.57) | **4.43 (3.99 to 4.87)** |
|  |  | Mid-low | 47.54 (46.55-48.53) | 42.31 (41.44-43.17) | 35.90 (35.10-36.71) | 30.72 (29.89-31.55) | 35.82 (34.51-37.14) | 32.64 (30.86-34.42) | 34.26 (32.27-36.26) | 35.32 (33.26-37.37) | **-3.99 (-4.33 to -3.65)** | -0.1 (-0.86 to 0.67) | **3.89 (3.05 to 4.73)** |
|  |  | low | 57.17 (55.81-58.52) | 52.39 (50.87-53.90) | 45.29 (43.93-46.64) | 42.98 (41.27-44.68) | 44.18 (41.67-46.69) | 43.86 (40.28-47.44) | 43.76 (39.45-48.08) | 45.91 (41.38-50.44) | **-4.06 (-4.62 to -3.50)** | 0.44 (-1.18 to 2.05) | **4.50 (2.79 to 6.21)** |
| Residential area | Rural | High | 36.03 (34.25-37.80) | 34.11 (32.30-35.92) | 26.42 (24.95-27.89) | 23.39 (22.18-24.59) | 23.93 (22.50-25.36) | 22.56 (20.32-24.80) | 25.87 (23.72-28.03) | 28.35 (26.04-30.66) | **-3.63 (-3.97 to -3.28)** | **1.18 (0.58 to 1.78)** | **4.81 (4.12 to 5.50)** |
|  |  | Mid-high | 37.03 (36.06-38.00) | 31.91 (30.99-32.83) | 25.51 (24.62-26.40) | 22.81 (22.01-23.61) | 24.96 (23.86-26.05) | 22.68 (21.30-24.06) | 24.90 (23.50-26.30) | 26.90 (25.39-28.41) | **-3.30 (-3.52 to -3.08)** | **0.41 (0.04 to 0.79)** | **3.71 (3.28 to 4.14)** |
|  |  | Middle | 37.88 (37.20-38.56) | 33.00 (32.32-33.68) | 27.15 (26.49-27.82) | 22.64 (22.01-23.27) | 25.08 (24.22-25.93) | 23.89 (22.70-25.07) | 25.09 (23.87-26.31) | 26.16 (24.99-27.33) | **-3.53 (-3.71 to -3.34)** | **0.40 (0.06 to 0.74)** | **3.93 (3.54 to 4.32)** |
|  |  | Mid-low | 46.10 (45.08-47.12) | 41.17 (40.12-42.22) | 35.85 (34.68-37.01) | 30.79 (29.72-31.87) | 35.56 (33.68-37.44) | 33.86 (31.79-35.93) | 37.57 (34.92-40.21) | 35.25 (32.57-37.92) | **-3.55 (-3.87 to -3.23)** | 0.61 (-0.11 to 1.33) | **4.16 (3.37 to 4.95)** |
|  |  | low | 55.24 (53.67-56.81) | 50.94 (49.13-52.76) | 43.39 (41.38-45.41) | 42.10 (39.69-44.52) | 42.06 (38.43-45.68) | 40.05 (35.23-44.86) | 42.80 (37.86-47.74) | 42.43 (36.53-48.34) | **-3.38 (-3.95 to -2.82)** | 0.89 (-0.69 to 2.48) | **4.27 (2.59 to 5.95)** |
|  | Urban | High | 37.75 (36.50-39.00) | 34.53 (33.27-35.79) | 27.16 (26.26-28.07) | 22.67 (21.88-23.46) | 25.30 (24.33-26.28) | 22.23 (20.76-23.70) | 25.84 (24.30-27.38) | 28.07 (26.46-29.68) | **-3.47 (-3.98 to -2.96)** | **1.67 (0.79 to 2.54)** | **5.14 (4.13 to 6.15)** |
|  |  | Mid-high | 37.49 (36.76-38.22) | 33.18 (32.52-33.85) | 27.21 (26.69-27.74) | 23.21 (22.70-23.73) | 26.29 (25.61-26.97) | 24.01 (23.05-24.97) | 25.43 (24.49-26.36) | 27.22 (26.27-28.18) | **-3.38 (-3.70 to -3.07)** | **0.80 (0.21 to 1.39)** | **4.18 (3.51 to 4.85)** |
|  |  | Middle | 38.62 (38.06-39.18) | 33.86 (33.35-34.37) | 27.54 (27.10-27.99) | 23.50 (23.04-23.95) | 27.15 (26.51-27.79) | 24.48 (23.62-25.34) | 25.88 (25.03-26.72) | 28.13 (27.28-28.99) | **-3.73 (-3.97 to -3.50)** | 0.43 (-0.03 to 0.89) | **4.16 (3.64 to 4.68)** |
|  |  | Mid-low | 46.65 (45.72-47.58) | 41.64 (40.83-42.46) | 36.18 (35.46-36.90) | 31.00 (30.18-31.83) | 36.85 (35.64-38.07) | 32.60 (30.85-34.35) | 33.99 (32.12-35.85) | 38.86 (36.94-40.78) | **-3.55 (-3.98 to -3.13)** | 0.27 (-0.75 to 1.30) | **3.82 (2.71 to 4.93)** |
|  |  | low | 55.90 (54.52-57.28) | 51.86 (50.42-53.30) | 46.49 (45.15-47.83) | 42.71 (40.99-44.44) | 46.29 (43.77-48.81) | 44.22 (40.52-47.92) | 45.99 (41.55-50.44) | 48.91 (44.58-53.24) | **-4.04 (-4.78 to -3.29)** | 0.36 (-1.81 to 2.53) | **4.40 (2.11 to 6.69)** |
| Recent alcohol consumption | No | High | 50.21 (48.08-52.35) | 30.31 (29.25-31.38) | 23.80 (23.00-24.60) | 20.49 (19.81-21.17) | 21.66 (20.79-22.53) | 20.84 (19.58-22.11) | 24.21 (22.91-25.50) | 25.85 (24.48-27.21) | **-3.20 (-3.52 to -2.88)** | **1.60 (1.08 to 2.12)** | **4.80 (4.19 to 5.41)** |
|  |  | Mid-high | 34.01 (33.36-34.66) | 29.99 (29.42-30.57) | 24.49 (24.01-24.96) | 21.35 (20.90-21.79) | 23.92 (23.32-24.51) | 22.18 (21.38-22.98) | 23.90 (23.10-24.70) | 25.51 (24.65-26.37) | **-2.90 (-3.10 to -2.71)** | **0.65 (0.32 to 0.99)** | **3.55 (3.16 to 3.94)** |
|  |  | Middle | 35.04 (34.56-35.51) | 30.93 (30.51-31.35) | 25.34 (24.95-25.72) | 21.50 (21.12-21.88) | 24.31 (23.78-24.83) | 22.64 (21.93-23.34) | 24.11 (23.43-24.80) | 25.86 (25.15-26.56) | **-3.20 (-3.36 to -3.05)** | **0.60 (0.32 to 0.88)** | **3.80 (3.48 to 4.12)** |
|  |  | Mid-low | 42.01 (41.24-42.79) | 38.75 (38.01-39.48) | 33.40 (32.73-34.07) | 28.46 (27.74-29.17) | 33.69 (32.56-34.81) | 30.97 (29.54-32.40) | 32.56 (30.97-34.16) | 36.57 (34.98-38.17) | **-3.08 (-3.36 to -2.80)** | **0.71 (0.06 to 1.36)** | **3.79 (3.08 to 4.50)** |
|  |  | low | 51.19 (49.86-52.52) | 47.24 (45.93-48.55) | 41.31 (39.96-42.67) | 37.99 (36.40-39.59) | 40.62 (38.13-43.11) | 40.13 (36.95-43.32) | 42.32 (38.65-45.99) | 42.72 (38.73-46.70) | **-3.53 (-4.08 to -2.98)** | 0.84 (-0.63 to 2.32) | **4.37 (2.80 to 5.94)** |
|  | Yes | High | 33.03 (31.82-34.25) | 49.57 (47.39-51.75) | 42.82 (40.70-44.94) | 36.06 (34.08-38.03) | 41.27 (38.96-43.59) | 35.37 (31.22-39.53) | 40.13 (35.79-44.47) | 43.35 (39.54-47.17) | **-3.12 (-3.81 to -2.42)** | 0.84 (-0.58 to 2.26) | **3.96 (2.38 to 5.54)** |
|  |  | Mid-high | 48.10 (46.94-49.26) | 45.29 (44.01-46.57) | 39.35 (38.09-40.62) | 33.83 (32.65-35.01) | 38.07 (36.52-39.63) | 37.34 (34.56-40.13) | 38.46 (35.85-41.07) | 38.97 (36.71-41.23) | **-3.32 (-3.74 to -2.90)** | 0.35 (-0.54 to 1.23) | **3.67 (2.69 to 4.65)** |
|  |  | Middle | 48.10 (47.27-48.93) | 44.62 (43.68-45.56) | 38.04 (37.13-38.94) | 32.70 (31.72-33.67) | 38.39 (37.12-39.65) | 38.85 (36.60-41.10) | 38.69 (36.52-40.86) | 38.92 (36.89-40.94) | **-3.47 (-3.79 to -3.15)** | 0.16 (-0.60 to 0.91) | **3.63 (2.81 to 4.45)** |
|  |  | Mid-low | 56.39 (55.10-57.69) | 50.08 (48.82-51.34) | 45.80 (44.49-47.11) | 40.71 (39.16-42.25) | 47.10 (44.93-49.27) | 44.95 (41.29-48.61) | 50.00 (45.87-54.14) | 58.15 (50.89-65.40) | **-3.53 (-4.04 to -3.01)** | 0.36 (-1.01 to 1.73) | **3.89 (2.43 to 5.35)** |
|  |  | low | 63.21 (61.52-64.89) | 60.70 (58.86-62.54) | 55.20 (53.13-57.26) | 54.52 (51.73-57.32) | 55.57 (51.87-59.27) | 54.08 (46.91-61.25) | 54.37 (47.36-61.37) | 60.00 (52.86-67.15) | **-2.61 (-3.39 to -1.82)** | 1.19 (-1.32 to 3.70) | **3.80 (1.17 to 6.43)** |
| Smoking status | No | High | 34.99 (33.89-36.09) | 31.52 (30.49-32.54) | 24.82 (24.02-25.62) | 21.25 (20.57-21.92) | 23.63 (22.79-24.48) | 21.67 (20.42-22.92) | 25.02 (23.78-26.27) | 26.77 (25.45-28.09) | **-3.22 (-3.53 to -2.92)** | **1.28 (0.78 to 1.79)** | **4.50 (3.91 to 5.09)** |
|  |  | Mid-high | 35.82 (35.19-36.44) | 31.35 (30.78-31.92) | 25.45 (24.98-25.91) | 22.19 (21.75-22.63) | 25.29 (24.71-25.88) | 23.28 (22.49-24.07) | 24.79 (24.00-25.58) | 26.38 (25.55-27.20) | **-3.06 (-3.25 to -2.87)** | **0.48 (0.15 to 0.80)** | **3.54 (3.16 to 3.92)** |
|  |  | Middle | 36.73 (36.28-37.18) | 32.10 (31.67-32.54) | 26.02 (25.64-26.40) | 22.47 (22.10-22.85) | 25.76 (25.24-26.29) | 23.87 (23.17-24.57) | 25.15 (24.46-25.85) | 26.83 (26.14-27.53) | **-3.30 (-3.45 to -3.15)** | **0.43 (0.15 to 0.71)** | **3.73 (3.41 to 4.05)** |
|  |  | Mid-low | 44.36 (43.60-45.13) | 39.89 (39.20-40.57) | 34.23 (33.58-34.87) | 29.67 (28.99-30.35) | 35.63 (34.58-36.69) | 32.48 (31.09-33.86) | 34.09 (32.54-35.65) | 35.74 (34.00-37.49) | **-3.23 (-3.50 to -2.96)** | 0.37 (-0.23 to 0.98) | **3.60 (2.94 to 4.26)** |
|  |  | low | 52.87 (51.66-54.08) | 47.98 (46.72-49.24) | 42.24 (40.97-43.52) | 39.22 (37.64-40.80) | 43.05 (40.87-45.24) | 41.74 (38.76-44.72) | 42.75 (39.34-46.15) | 43.92 (40.24-47.60) | **-3.42 (-3.92 to -2.92)** | 0.33 (-1.01 to 1.67) | **3.75 (2.32 to 5.18)** |
|  | Yes | High | 52.33 (49.49-55.17) | 50.53 (47.82-53.24) | 43.16 (40.62-45.69) | 39.52 (36.94-42.10) | 41.73 (38.13-45.32) | 45.48 (36.77-54.20) | 45.07 (37.67-52.48) | 50.47 (44.35-56.60) | **-3.42 (-4.40 to -2.45)** | **2.71 (0.43 to 4.98)** | **6.13 (3.65 to 8.61)** |
|  |  | Mid-high | 50.40 (48.75-52.05) | 45.49 (43.81-47.17) | 39.83 (38.17-41.48) | 36.97 (35.05-38.90) | 38.60 (35.85-41.36) | 40.65 (34.60-46.69) | 43.10 (37.73-48.48) | 49.59 (45.24-53.93) | **-3.62 (-4.27 to -2.97)** | **3.48 (1.84 to 5.11)** | **7.10 (5.34 to 8.86)** |
|  |  | Middle | 51.00 (49.78-52.21) | 45.40 (44.24-46.55) | 39.94 (38.81-41.08) | 33.83 (32.43-35.24) | 40.00 (37.55-42.46) | 45.71 (40.47-50.94) | 42.53 (38.19-46.86) | 47.40 (43.39-51.41) | **-4.07 (-4.59 to -3.56)** | **2.12 (0.65 to 3.59)** | **6.19 (4.63 to 7.75)** |
|  |  | Mid-low | 58.03 (56.27-59.80) | 50.68 (49.02-52.33) | 47.65 (45.98-49.32) | 42.61 (40.29-44.93) | 46.10 (42.53-49.67) | 52.38 (44.23-60.54) | 57.75 (50.86-64.64) | 46.88 (43.11-50.64) | **-3.98 (-4.73 to -3.24)** | **4.38 (1.94 to 6.82)** | **8.36 (5.81 to 10.91)** |
|  |  | low | 64.10 (61.89-66.32) | 62.42 (60.21-64.64) | 55.65 (53.37-57.93) | 55.59 (52.39-58.80) | 55.32 (49.94-60.70) | 58.99 (46.32-71.67) | 66.42 (55.14-77.69) | 66.47 (56.97-75.98) | **-2.97 (-3.98 to -1.95)** | **4.02 (0.54 to 7.50)** | **6.99 (3.37 to 10.62)** |
| Parental educational attainment | High school diploma or less | High | 34.96 (33.45-36.47) | 34.60 (32.76-36.45) | 27.11 (25.65-28.58) | 21.82 (20.58-23.06) | 23.58 (22.31-24.86) | 20.92 (18.95-22.89) | 26.07 (24.16-27.99) | 28.70 (26.63-30.77) | **-3.37 (-3.82 to -2.92)** | **2.05 (1.27 to 2.83)** | **5.42 (4.52 to 6.32)** |
|  |  | Mid-high | 36.02 (35.13-36.91) | 31.37 (30.53-32.20) | 25.70 (24.92-26.49) | 21.62 (20.88-22.35) | 24.92 (24.00-25.83) | 23.32 (22.12-24.51) | 25.36 (24.13-26.59) | 26.41 (25.16-27.66) | **-3.19 (-3.47 to -2.91)** | **0.65 (0.16 to 1.14)** | **3.84 (3.28 to 4.40)** |
|  |  | Middle | 36.74 (36.22-37.26) | 32.48 (31.96-33.00) | 26.67 (26.18-27.16) | 21.72 (21.23-22.22) | 25.11 (24.44-25.78) | 23.94 (23.11-24.78) | 25.41 (24.52-26.30) | 27.45 (26.53-28.37) | **-3.55 (-3.73 to -3.37)** | **0.82 (0.46 to 1.18)** | **4.37 (3.97 to 4.77)** |
|  |  | Mid-low | 45.40 (44.62-46.19) | 40.43 (39.65-41.21) | 35.20 (34.43-35.96) | 29.56 (28.73-30.39) | 35.77 (34.51-37.02) | 31.83 (30.10-33.55) | 35.57 (33.66-37.49) | 36.56 (34.55-38.57) | **-3.54 (-3.85 to -3.24)** | 0.52 (-0.22 to 1.27) | **4.06 (3.25 to 4.87)** |
|  |  | low | 55.12 (53.95-56.29) | 50.66 (49.35-51.97) | 44.30 (43.01-45.60) | 41.95 (40.22-43.68) | 44.06 (41.50-46.62) | 42.08 (38.72-45.43) | 42.23 (38.58-45.88) | 45.62 (41.41-49.82) | **-3.69 (-4.22 to -3.15)** | 0.40 (-1.14 to 1.95) | **4.09 (2.45 to 5.73)** |
|  | Bachelor's degree or higher | High | 38.66 (37.35-39.96) | 34.33 (33.13-35.53) | 26.90 (25.99-27.81) | 23.23 (22.47-23.99) | 25.75 (24.68-26.82) | 23.25 (21.70-24.81) | 25.70 (24.09-27.30) | 27.83 (26.07-29.58) | **-3.70 (-4.06 to -3.34)** | **0.86 (0.21 to 1.52)** | **4.56 (3.81 to 5.31)** |
|  |  | Mid-high | 38.24 (37.46-39.01) | 33.53 (32.87-34.20) | 27.17 (26.64-27.69) | 23.66 (23.16-24.16) | 26.49 (25.80-27.17) | 23.84 (22.83-24.86) | 25.23 (24.20-26.26) | 27.53 (26.51-28.55) | **-3.43 (-3.65 to -3.21)** | **0.44 (0.05 to 0.84)** | **3.87 (3.42 to 4.32)** |
|  |  | Middle | 40.78 (40.08-41.48) | 34.86 (34.28-35.43) | 28.16 (27.68-28.65) | 24.46 (24.00-24.92) | 27.91 (27.25-28.57) | 24.68 (23.69-25.68) | 25.87 (24.97-26.78) | 27.53 (26.62-28.43) | **-3.78 (-3.99 to -3.58)** | -0.02 (-0.38 to 0.35) | **3.76 (3.34 to 4.18)** |
|  |  | Mid-low | 49.18 (47.78-50.58) | 43.56 (42.52-44.60) | 37.54 (36.62-38.46) | 32.86 (31.86-33.85) | 37.47 (35.87-39.08) | 34.94 (32.74-37.14) | 34.64 (32.35-36.93) | 39.21 (36.86-41.56) | **-3.83 (-4.27 to -3.39)** | 0.42 (-0.48 to 1.32) | **4.25 (3.25 to 5.25)** |
|  |  | low | 57.67 (55.40-59.94) | 54.06 (51.94-56.17) | 48.38 (46.28-50.48) | 43.72 (41.30-46.13) | 46.73 (42.97-50.48) | 44.56 (38.70-50.42) | 52.70 (46.19-59.21) | 49.99 (43.45-56.53) | **-3.64 (-4.51 to -2.77)** | 1.72 (-0.66 to 4.09) | **5.36 (2.83 to 7.89)** |
| Academic achievement | Low | High | 36.24 (34.91-37.57) | 32.53 (31.39-33.66) | 25.49 (24.51-26.48) | 21.86 (21.02-22.69) | 24.04 (23.00-25.08) | 20.55 (19.12-21.97) | 24.21 (22.75-25.68) | 25.17 (23.58-26.77) | **-3.47 (-3.83 to -3.10)** | **0.69 (0.09 to 1.30)** | **4.16 (3.45 to 4.87)** |
|  |  | Mid-high | 34.19 (33.43-34.94) | 29.66 (28.96-30.36) | 23.29 (22.71-23.87) | 20.07 (19.53-20.61) | 23.06 (22.31-23.81) | 21.24 (20.16-22.32) | 21.80 (20.77-22.82) | 24.09 (23.03-25.15) | **-3.27 (-3.50 to -3.04)** | 0.37 (-0.05 to 0.78) | **3.64 (3.17 to 4.11)** |
|  |  | Middle | 35.57 (34.90-36.24) | 29.42 (28.79-30.06) | 23.85 (23.26-24.43) | 20.16 (19.63-20.70) | 23.41 (22.65-24.16) | 21.14 (20.08-22.20) | 22.52 (21.38-23.66) | 24.56 (23.35-25.77) | **-3.60 (-3.82 to -3.38)** | 0.43 (-0.01 to 0.88) | **4.03 (3.53 to 4.53)** |
|  |  | Mid-low | 42.90 (41.67-44.12) | 36.98 (35.81-38.16) | 30.44 (29.36-31.51) | 27.86 (26.66-29.05) | 32.57 (30.71-34.42) | 31.36 (28.78-33.94) | 31.93 (28.84-35.02) | 35.83 (32.77-38.89) | **-3.61 (-4.06 to -3.16)** | 0.97 (-0.16 to 2.10) | **4.58 (3.36 to 5.80)** |
|  |  | low | 52.45 (50.21-54.69) | 46.69 (44.18-49.21) | 41.78 (39.20-44.35) | 38.40 (35.41-41.39) | 41.47 (37.30-45.64) | 33.87 (27.30-40.44) | 47.82 (40.63-55.00) | 49.45 (42.16-56.75) | **-3.60 (-4.54 to -2.65)** | **3.39 (0.79 to 6.00)** | **6.99 (4.22 to 9.76)** |
|  | Middle | High | 36.15 (33.83-38.47) | 32.37 (29.86-34.87) | 25.28 (23.43-27.13) | 21.78 (20.22-23.34) | 23.53 (21.71-25.35) | 21.34 (18.70-23.98) | 25.82 (22.85-28.78) | 30.64 (27.46-33.81) | **-3.47 (-4.12 to -2.81)** | **2.56 (1.40 to 3.71)** | **6.03  (4.70 to 7.36)** |
|  |  | Mid-high | 38.54 (37.40-39.67) | 33.20 (32.20-34.21) | 26.98 (26.17-27.79) | 23.70 (22.93-24.48) | 25.88 (24.86-26.90) | 24.18 (22.76-25.59) | 26.06 (24.55-27.57) | 27.42 (25.87-28.96) | **-3.48 (-3.82 to -3.15)** | **0.64 (0.05 to 1.23)** | **4.12  (3.44 to 4.80)** |
|  |  | Middle | 37.10 (36.42-37.79) | 32.47 (31.83-33.11) | 25.64 (25.09-26.20) | 22.11 (21.55-22.67) | 24.92 (24.18-25.66) | 22.39 (21.41-23.36) | 23.67 (22.67-24.66) | 25.23 (24.16-26.30) | **-3.60 (-3.81 to -3.38)** | 0.20 (-0.21 to 0.61) | **3.80  (3.34 to 4.26)** |
|  |  | Mid-low | 45.08 (43.79-46.37) | 40.20 (39.01-41.39) | 34.33 (33.18-35.49) | 28.57 (27.29-29.85) | 35.11 (33.21-37.01) | 31.48 (28.79-34.16) | 33.38 (30.36-36.40) | 32.93 (30.07-35.79) | **-3.67 (-4.15 to -3.20)** | -0.50 (-1.60 to 0.59) | **3.17  (1.98 to 4.36)** |
|  |  | low | 56.52 (53.89-59.16) | 50.32 (47.50-53.14) | 40.99 (38.21-43.78) | 39.10 (35.51-42.68) | 43.10 (37.92-48.28) | 41.38 (34.01-48.75) | 44.58 (36.65-52.51) | 42.58 (33.92-51.24) | **-4.71 (-5.85 to -3.58)** | 0.19 (-2.96 to 3.33) | **4.90  (1.56 to 8.24)** |
|  | High | High | 40.94 (38.68-43.20) | 42.42 (40.02-44.83) | 32.59 (30.80-34.39) | 26.76 (25.32-28.21) | 29.21 (27.19-31.23) | 28.29 (25.34-31.24) | 31.19 (28.08-34.30) | 36.25 (33.07-39.43) | **-3.88 (-4.55 to -3.21)** | **2.40 (1.19 to 3.61)** | **6.28  (4.90 to 7.66)** |
|  |  | Mid-high | 43.56 (42.35-44.76) | 39.21 (38.12-40.29) | 33.73 (32.79-34.66) | 29.25 (28.33-30.16) | 32.72 (31.49-33.95) | 28.24 (26.50-29.98) | 32.81 (30.93-34.68) | 34.20 (32.51-35.89) | **-3.26 (-3.64 to -2.89)** | **0.89 (0.21 to 1.56)** | **4.15  (3.38 to 4.92)** |
|  |  | Middle | 41.97 (41.27-42.66) | 37.64 (37.02-38.27) | 31.52 (30.94-32.10) | 26.80 (26.21-27.39) | 30.51 (29.70-31.32 | 28.47 (27.36-29.58) | 29.89 (28.79-30.99) | 31.95 (30.84-33.05) | **-3.57 (-3.80 to -3.34)** | **0.56 (0.12 to 0.99)** | **4.13  (3.64 to 4.62)** |
|  |  | Mid-low | 49.10 (48.11-50.10) | 44.33 (43.42-45.23) | 39.53 (38.71-40.35) | 33.62 (32.70-34.54) | 38.90 (37.49-40.32) | 34.52 (32.55-36.49) | 37.41 (35.41-39.42) | 40.88 (38.71-43.04) | **-3.53 (-3.89 to -3.18)** | 0.75 (-0.06 to 1.56) | **4.28  (3.40 to 5.16)** |
|  |  | low | 56.49 (55.16-57.81) | 53.11 (51.73-54.49) | 47.49 (46.12-48.86) | 44.81 (42.98-46.64) | 46.53 (43.97-49.08) | 45.54 (41.72-49.36) | 43.95 (39.65-48.26) | 47.06 (42.80-51.31) | **-3.28 (-3.84 to -2.71)** | -0.02 (-1.61 to 1.56) | **3.26  (1.58 to 4.94)** |
| **Suicidal ideation** | | | | | | | | | | | | | |
| Grade level | Middle school (7-9^th^) | High | 20.04 (19.05-21.03) | 18.88 (17.86-19.90) | 14.74 (13.87-15.62) | 10.61 (10.02-11.20) | 11.02 (10.18-11.85) | 7.68 (6.79-8.56) | 11.00 (9.80-12.19) | 13.76 (12.42-15.11) | **-2.63  (-2.91 to -2.34)** | **1.19  (0.68 to 1.70)** | **3.82 (3.24 to 4.40)** |
|  |  | Mid-high | 19.42 (18.81-20.02) | 17.10 (16.53-17.66) | 14.03 (13.58-14.47) | 10.36 (9.95-10.78) | 9.06 (8.38-9.75) | 10.36 (9.95-10.78) | 11.81 (11.06-12.55) | 13.54 (12.61-14.46) | **-2.23  (-2.41 to -2.05)** | **0.78  (0.44 to 1.12)** | **3.01 (2.63 to 3.39)** |
|  |  | Middle | 20.36 (19.87-20.85) | 17.72 (17.24-18.19) | 15.23 (14.82-15.65) | 11.50 (11.12-11.87) | 13.72 (13.20-14.24) | 9.65 (9.02-10.28) | 13.07 (12.36-13.78) | 14.82 (14.01-15.63) | **-2.23  (-2.41 to -2.05)** | **0.78  (0.44 to 1.12)** | **3.01 (2.63 to 3.39)** |
|  |  | Mid-low | 28.45 (27.52-29.38) | 25.50 (24.62-26.38) | 24.59 (23.70-25.48) | 20.15 (19.16-21.13) | 24.80 (23.37-26.22) | 19.46 (17.73-21.19) | 23.14 (21.04-25.25) | 25.47 (23.26-27.67) | **-1.52  (-1.87 to -1.17)** | 0.56  (-0.28 to 1.40) | **2.08 (1.17 to 2.99)** |
|  |  | low | 37.77 (36.16-39.39) | 35.18 (33.54-36.81) | 34.44 (32.68-36.20) | 28.24 (25.97-30.51) | 34.84 (31.35-38.32) | 26.53 (21.86-31.21) | 33.50 (28.49-38.50) | 36.47 (31.31-41.63) | **-1.63  (-2.36 to -0.90)** | 1.22  (-0.78 to 3.22) | **2.85 (0.72 to 4.98)** |
|  | High school (10-12^th^) | High | 22.42 (20.68-24.15) | 20.04 (18.62-21.47) | 16.59  (15.52-17.66) | 12.49 (11.57-13.42) | 12.91 (11.83-13.99) | 10.45 (8.87-12.02) | 10.66 (9.23-12.10) | 14.19 (12.50-15.88) | **-2.62  (-3.06 to -2.19)** | 0.36  (-0.27 to 0.99) | **2.98 (2.21 to 3.75)** |
|  |  | Mid-high | 19.26 (18.54-19.99) | 16.83 (16.19-17.46) | 13.99 (13.49-14.50) | 10.93 (10.46-11.40) | 10.70 (9.74-11.66) | 10.93 (10.46-11.40) | 11.03 (10.14-11.92) | 11.37 (10.56-12.19) | **-2.26  (-2.46 to -2.06)** | 0.16  (-0.17 to 0.48) | **2.42 (2.04 to 2.80)** |
|  |  | Middle | 19.65 (19.10-20.21) | 16.58 (16.09-17.06) | 13.17 (12.79-13.55) | 9.86 (9.53-10.19) | 11.04 (10.58-11.50) | 10.49 (9.81-11.17) | 10.79 (10.04-11.55) | 11.85 (11.20-12.49) | **-2.26  (-2.46 to -2.06)** | 0.16  (-0.17 to 0.48) | **2.42 (2.04 to 2.80)** |
|  |  | Mid-low | 25.27 (24.47-26.07) | 22.52 (21.80-23.25) | 18.75 (18.12-19.37) | 15.46 (14.81-16.12) | 18.32 (17.30-19.34) | 15.31 (13.84-16.78) | 18.04 (16.42-19.66) | 19.80 (18.07-21.54) | **-2.36  (-2.63 to -2.09)** | 0.59  (-0.03 to 1.22) | **2.95 (2.27 to 3.63)** |
|  |  | low | 34.98 (33.61-36.35) | 31.96 (30.54-33.38) | 28.73 (27.47-29.99) | 26.06 (24.49-27.62) | 26.10 (23.78-28.42) | 23.03 (19.81-26.26) | 26.01 (22.40-29.62) | 28.22 (23.99-32.45) | **-2.57  (-3.10 to -2.03)** | 0.78  (-0.70 to 2.26) | **3.35 (1.78 to 4.92)** |
| Residential area | Rural | High | 21.66 (20.53-22.80) | 19.45 (18.37-20.52) | 15.58 (14.66-16.50) | 11.52 (10.78-12.26) | 11.61 (10.67-12.54) | 8.76 (7.51-10.01) | 10.24 (9.05-11.43) | 13.62 (12.05-15.18) | **-2.69  (-2.98 to -2.40)** | **0.83  (0.34 to 1.32)** | **3.52 (2.95 to 4.09)** |
|  |  | Mid-high | 19.47 (18.84-20.11) | 17.15 (16.55-17.75) | 14.62 (14.13-15.10) | 11.21 (10.74-11.68) | 9.64 (8.74-10.53) | 11.21 (10.74-11.68) | 11.08 (10.21-11.95) | 12.37 (11.41-13.32) | **-2.23  (-2.39 to -2.07)** | **0.54  (0.26 to 0.83)** | **2.77 (2.44 to 3.10)** |
|  |  | Middle | 20.06 (19.53-20.58) | 17.19 (16.73-17.65) | 14.41 (14.00-14.83) | 10.47 (10.08-10.85) | 12.15 (11.61-12.69) | 9.49 (8.75-10.24) | 11.19 (10.40-11.97) | 13.00 (12.20-13.80) | **-2.23  (-2.39 to -2.07)** | **0.54  (0.26 to 0.83)** | **2.77 (2.44 to 3.10)** |
|  |  | Mid-low | 26.22 (25.38-27.06) | 23.35 (22.57-24.12) | 20.89 (20.11-21.66) | 16.44 (15.64-17.24) | 20.87 (19.60-22.14) | 16.55 (14.91-18.18) | 18.74 (16.74-20.74) | 21.41 (19.41-23.42) | **-2.23  (-2.50 to -1.97)** | **0.84  (0.20 to 1.47)** | **3.07 (2.38 to 3.76)** |
|  |  | low | 35.93 (34.52-37.33) | 33.01 (31.42-34.60) | 31.22 (29.65-32.78) | 26.87 (24.90-28.84) | 29.18 (26.31-32.06) | 21.33 (17.47-25.18) | 27.68 (23.79-31.56) | 30.30 (25.64-34.96) | **-2.23  (-2.76 to -1.69)** | **1.54  (0.04 to 3.04)** | **3.77 (2.18 to 5.36)** |
|  | Urban | High | 20.01 (18.70-21.32) | 19.20 (17.94-20.45) | 15.26 (14.27-16.25) | 11.24 (10.53-11.95) | 11.96 (11.02-12.90) | 8.66 (7.60-9.73) | 11.38 (10.03-12.73) | 14.16 (12.75-15.57) | **-2.32  (-2.72 to -1.91)** | **0.92  (0.27 to 1.57)** | **3.24 (2.47 to 4.01)** |
|  |  | Mid-high | 19.25 (18.57-19.92) | 16.84 (16.25-17.44) | 13.50 (13.04-13.96) | 10.16 (9.74-10.57) | 9.95 (9.19-10.70) | 10.16 (9.74-10.57) | 11.75 (10.98-12.51) | 12.74 (11.88-13.59) | **-2.28  (-2.52 to -2.04)** | 0.39  (-0.04 to 0.82) | **2.67 (2.18 to 3.16)** |
|  |  | Middle | 19.99 (19.47-20.51) | 17.10 (16.61-17.59) | 13.97 (13.59-14.34) | 10.65 (10.32-10.98) | 12.30 (11.85-12.75) | 10.51 (9.91-11.12) | 12.39 (11.69-13.08) | 13.45 (12.79-14.12) | **-2.28  (-2.52 to -2.04)** | 0.39  (-0.04 to 0.82) | **2.67 (2.18 to 3.16)** |
|  |  | Mid-low | 26.99 (26.12-27.87) | 24.07 (23.28-24.87) | 20.91 (20.21-21.60) | 17.43 (16.68-18.17) | 20.45 (19.34-21.56) | 17.23 (15.68-18.79) | 20.96 (19.27-22.65) | 22.86 (20.98-24.75) | **-1.96  (-2.32 to -1.61)** | 0.44  (-0.40 to 1.28) | **2.40 (1.49 to 3.31)** |
|  |  | low | 36.32 (34.80-37.85) | 33.39 (31.93-34.85) | 30.17 (28.81-31.54) | 26.53 (24.82-28.24) | 28.49 (25.85-31.13) | 26.46 (22.82-30.09) | 29.34 (25.09-33.60) | 32.73 (28.22-37.24) | **-2.71  (-3.43 to -1.99)** | 0.44  (-1.45 to 2.34) | **3.15 (1.12 to 5.18)** |
| Recent alcohol consumption | No | High | 17.61 (16.67-18.55) | 16.84 (15.98-17.69) | 13.24 (12.54-13.94) | 10.06 (9.53-10.59) | 9.70 (9.06-10.35) | 7.80 (6.97-8.63) | 9.72 (8.78-10.65) | 12.31 (11.22-13.39) | **-2.26  (-2.50 to -2.01)** | **0.99  (0.59 to 1.40)** | **3.25 (2.78 to 3.72)** |
|  |  | Mid-high | 17.48 (16.96-17.99) | 15.32 (14.88-15.76) | 12.60 (12.25-12.94) | 9.72 (9.41-10.04) | 8.83 (8.28-9.39) | 9.72 (9.41-10.04) | 10.76 (10.18-11.35) | 11.50 (10.85-12.15) | **-1.99  (-2.14 to -1.85)** | **0.53  (0.28 to 0.78)** | **2.52 (2.23 to 2.81)** |
|  |  | Middle | 18.05 (17.64-18.45) | 15.60 (15.26-15.94) | 12.82 (12.53-13.11) | 9.83 (9.57-10.09) | 11.26 (10.91-11.62) | 9.19 (8.73-9.65) | 11.22 (10.71-11.72) | 12.33 (11.79-12.87) | **-1.99  (-2.14 to -1.85)** | **0.53  (0.28 to 0.78)** | **2.52 (2.23 to 2.81)** |
|  |  | Mid-low | 24.01 (23.35-24.66) | 21.72 (21.13-22.31) | 19.29 (18.75-19.84) | 15.76 (15.18-16.34) | 18.56 (17.65-19.47) | 15.60 (14.48-16.73) | 18.28 (16.96-19.61) | 20.83 (19.31-22.34) | **-1.88  (-2.11 to -1.65)** | **0.89  (0.34 to 1.44)** | **2.77 (2.17 to 3.37)** |
|  |  | low | 31.98 (30.67-33.29) | 29.28 (28.06-30.51) | 26.73 (25.56-27.91) | 23.83 (22.36-25.29) | 25.47 (23.27-27.66) | 21.63 (18.74-24.52) | 24.21 (21.16-27.26) | 28.72 (25.11-32.34) | **-2.09  (-2.60 to -1.59)** | 1.15  (-0.17 to 2.47) | **3.24 (1.83 to 4.65)** |
|  | Yes | High | 30.57 (28.64-32.49) | 28.47 (26.53-30.41) | 26.32 (24.36-28.29) | 18.65 (16.98-20.32) | 22.24 (20.27-24.22) | 16.73 (13.52-19.94) | 20.90 (17.57-24.22) | 24.57 (21.07-28.06) | **-2.63  (-3.24 to -2.02)** | 0.87  (-0.39 to 2.13) | **3.50 (2.10 to 4.90)** |
|  |  | Mid-high | 25.38 (24.33-26.42) | 24.34 (23.25-25.43) | 22.04 (20.98-23.10) | 16.22 (15.27-17.18) | 19.03 (16.76-21.29) | 16.22 (15.27-17.18) | 18.12 (15.87-20.38) | 20.47 (18.45-22.48) | **-2.31  (-2.66 to -1.96)** | 0.73  (-0.02 to 1.48) | **3.04 (2.21 to 3.87)** |
|  |  | Middle | 25.73 (24.98-26.49) | 23.51 (22.69-24.32) | 20.96 (20.22-21.70) | 14.70 (14.01-15.38) | 17.61 (16.66-18.55) | 18.08 (16.28-19.89) | 17.80 (16.03-19.58) | 19.86 (18.20-21.52) | **-2.31  (-2.66 to -1.96)** | 0.73  (-0.02 to 1.48) | **3.04 (2.21 to 3.87)** |
|  |  | Mid-low | 32.43 (31.24-33.63) | 30.04 (28.86-31.22) | 26.72 (25.54-27.91) | 21.92 (20.61-23.22) | 28.70 (26.65-30.76) | 24.78 (21.59-27.98) | 29.80 (25.95-33.65) | 29.21 (25.68-32.74) | **-2.03  (-2.50 to -1.55)** | 0.46  (-0.82 to 1.74) | **2.49 (1.12 to 3.86)** |
|  |  | low | 43.12 (41.34-44.90) | 41.52 (39.65-43.40) | 39.71 (37.72-41.70) | 34.24 (31.57-36.91) | 37.16 (33.33-41.00) | 35.20 (28.57-41.82) | 45.31 (38.00-52.61) | 41.24 (34.27-48.20) | **-2.03  (-2.84 to -1.23)** | 2.08  (-0.41 to 4.57) | **4.11 (1.49 to 6.73)** |
| Smoking status | No | High | 19.00 (18.13-19.88) | 17.03 (16.24-17.82) | 13.57 (12.90-14.24) | 10.22 (9.72-10.72) | 10.70 (10.04-11.37) | 8.22 (7.41-9.03) | 10.19 (9.29-11.08) | 36.82 (31.00-42.63) | **-2.32  (-2.55 to -2.08)** | **0.75  (0.35 to 1.14)** | **3.07 (2.61 to 3.53)** |
|  |  | Mid-high | 18.24 (17.75-18.72) | 15.94 (15.50-16.37) | 13.05 (12.71-13.39) | 10.21 (9.89-10.52) | 9.63 (9.05-10.20) | 10.21 (9.89-10.52) | 11.18 (10.60-11.77) | 12.10 (11.45-12.74) | **-2.02  (-2.16 to -1.89)** | **0.46  (0.22 to 0.70)** | **2.48 (2.20 to 2.76)** |
|  |  | Middle | 18.90 (18.50-19.30) | 16.12 (15.78-16.47) | 13.20 (12.92-13.49) | 10.18 (9.93-10.43) | 11.93 (11.58-12.28) | 9.85 (9.37-10.32) | 11.63 (11.12-12.15) | 12.88 (12.36-13.39) | **-2.02  (-2.16 to -1.89)** | **0.46  (0.22 to 0.70)** | **2.48 (2.20 to 2.76)** |
|  |  | Mid-low | 25.31 (24.67-25.94) | 22.48 (21.89-23.07) | 19.74 (19.20-20.28) | 16.38 (15.81-16.95) | 19.90 (19.04-20.77) | 16.56 (15.43-17.68) | 19.06 (17.78-20.34) | 21.41 (20.00-22.82) | **-1.94  (-2.17 to -1.72)** | **0.63  (0.11 to 1.15)** | **2.57 (2.00 to 3.14)** |
|  |  | low | 33.27 (32.10-34.45) | 30.03 (28.87-31.20) | 27.67 (26.52-28.82) | 24.48 (23.07-25.88) | 26.65 (24.66-28.65) | 22.80 (20.15-25.44) | 26.66 (23.69-29.64) | 29.21 (25.78-32.64) | **-2.15  (-2.62 to -1.68)** | 1.07  (-0.16 to 2.31) | **3.22 (1.90 to 4.54)** |
|  | Yes | High | 32.70 (29.95-35.46) | 32.05 (29.53-34.56) | 29.41 (27.03-31.79) | 23.21 (20.97-25.45) | 25.92 (22.73-29.10) | 25.93 (18.65-33.20) | 26.74 (20.29-33.19) | 14.33 (12.67-15.98) | **-2.33  (-3.23 to -1.44)** | **3.30  (1.20 to 5.40)** | **5.63 (3.35 to 7.91)** |
|  |  | Mid-high | 28.81 (27.29-30.33) | 26.03 (24.57-27.48) | 23.98 (22.53-25.42) | 17.12 (15.61-18.64) | 18.83 (14.15-23.50) | 17.12 (15.61-18.64) | 21.61 (17.34-25.89) | 27.02 (22.69-31.36) | **-2.99  (-3.55 to -2.44)** | **2.58  (1.07 to 4.09)** | **5.57 (3.96 to 7.18)** |
|  |  | Middle | 28.53 (27.36-29.70) | 25.20 (24.15-26.25) | 22.71 (21.71-23.72) | 16.25 (15.17-17.32) | 18.14 (16.37-19.91) | 23.03 (18.78-27.29) | 21.53 (18.03-25.03) | 25.17 (21.60-28.74) | **-2.99  (-3.55 to -2.44)** | **2.58  (1.07 to 4.09)** | **5.57 (3.96 to 7.18)** |
|  |  | Mid-low | 33.80 (32.03-35.57) | 30.91 (29.45-32.38) | 28.17 (26.70-29.63) | 22.75 (20.86-24.64) | 29.53 (26.16-32.91) | 30.92 (22.87-38.97) | 39.64 (32.27-47.01) | 38.47 (31.59-45.35) | **-2.33  (-3.04 to -1.62)** | **3.49  (1.15 to 5.83)** | **5.82 (3.37 to 8.27)** |
|  |  | low | 44.74 (42.50-46.98) | 42.86 (40.65-45.06) | 39.92 (37.71-42.13) | 35.40 (32.26-38.53) | 41.09 (35.94-46.23) | 46.02 (33.36-58.67) | 48.73 (36.79-60.67) | 48.49 (38.71-58.27) | **-2.09  (-3.09 to -1.09)** | 2.61  (-0.89 to 6.11) | **4.70 (1.06 to 8.34)** |
| Parental educational attainment | High school diploma or less | High | 20.43 (19.07-21.78) | 20.14 (18.68-21.60) | 16.82 (15.53-18.11) | 10.87 (9.88-11.86) | 10.90 (9.90-11.90) | 8.22 (7.04-9.39) | 10.62 (9.23-12.01) | 13.68 (12.37-15.00) | **-2.74  (-3.12 to -2.36)** | **1.26  (0.65 to 1.87)** | **4.00 (3.28 to 4.72)** |
|  |  | Mid-high | 18.53 (17.86-19.19) | 16.16 (15.50-16.83) | 13.19 (12.62-13.76) | 9.65 (9.13-10.17) | 9.34 (8.53-10.15) | 9.65 (9.13-10.17) | 10.92 (10.03-11.82) | 12.03 (11.08-12.98) | **-2.24  (-2.44 to -2.04)** | **0.62  (0.26 to 0.98)** | **2.86 (2.45 to 3.27)** |
|  |  | Middle | 19.17 (18.73-19.61) | 16.33 (15.91-16.76) | 13.71 (13.35-14.06) | 9.81 (9.48-10.14) | 11.33 (10.90-11.76) | 9.85 (9.30-10.40) | 11.12 (10.46-11.78) | 12.76 (12.08-13.43) | **-2.24  (-2.44 to -2.04)** | **0.62  (0.26 to 0.98)** | **2.86 (2.45 to 3.27)** |
|  |  | Mid-low | 26.24 (25.53-26.94) | 23.05 (22.40-23.70) | 20.26 (19.62-20.89) | 16.33 (15.64-17.03) | 20.20 (19.18-21.22) | 15.75 (14.39-17.11) | 19.13 (17.58-20.68) | 22.35 (20.44-24.26) | **-2.19  (-2.45 to -1.93)** | **0.85  (0.18 to 1.51)** | **3.04 (2.33 to 3.75)** |
|  |  | low | 35.58 (34.44-36.72) | 32.35 (31.10-33.60) | 30.10 (28.88-31.31) | 26.41 (24.84-27.99) | 27.95 (25.65-30.25) | 23.86 (20.68-27.04) | 26.08 (22.92-29.24) | 30.83 (26.90-34.76) | **-2.37  (-2.87 to -1.88)** | 0.96  (-0.46 to 2.37) | **3.33 (1.83 to 4.83)** |
|  | Bachelor's degree or higher | High | 21.03 (19.98-22.09) | 18.97 (17.99-19.96) | 14.87 (14.11-15.63) | 11.54 (10.95-12.14) | 12.34 (11.50-13.17) | 9.03 (8.00-10.07) | 11.04 (9.82-12.27) | 13.16 (11.82-14.50) | **-2.5  (-2.79 to -2.21)** | **0.60  (0.10 to 1.10)** | **3.10 (2.52 to 3.68)** |
|  |  | Mid-high | 19.90 (19.28-20.52) | 17.39 (16.87-17.91) | 14.38 (13.98-14.77) | 11.01 (10.65-11.37) | 10.13 (9.41-10.85) | 11.01 (10.65-11.37) | 11.81 (11.08-12.53) | 12.88 (12.11-13.65) | **-2.27  (-2.44 to -2.10)** | **0.43  (0.13 to 0.72)** | **2.70 (2.36 to 3.04)** |
|  |  | Middle | 21.25 (20.68-21.82) | 18.05 (17.59-18.52) | 14.61 (14.23-14.99) | 11.21 (10.87-11.54) | 13.20 (12.71-13.69) | 10.38 (9.71-11.05) | 12.78 (12.08-13.48) | 13.75 (13.08-14.42) | **-2.27  (-2.44 to -2.10)** | **0.43  (0.13 to 0.72)** | **2.70 (2.36 to 3.04)** |
|  |  | Mid-low | 27.59 (26.49-28.69) | 25.07 (24.14-25.99) | 21.97 (21.18-22.76) | 17.93 (17.08-18.79) | 21.32 (19.95-22.68) | 18.88 (17.08-20.68) | 21.48 (19.44-23.52) | 22.12 (20.06-24.18) | **-2.21  (-2.57 to -1.85)** | 0.45  (-0.33 to 1.24) | **2.66 (1.80 to 3.52)** |
|  |  | low | 38.16 (35.78-40.53) | 35.59 (33.64-37.54) | 31.93 (29.99-33.87) | 27.22 (25.00-29.45) | 30.73 (27.22-34.23) | 25.10 (20.25-29.95) | 36.25 (29.67-42.83) | 33.97 (27.72-40.22) | **-2.65  (-3.50 to -1.80)** | 1.90  (-0.35 to 4.16) | **4.55 (2.14 to 6.96)** |
| Academic achievement | Low | High | 19.96 (18.89-21.02) | 18.74 (17.80-19.68) | 15.01 (14.18-15.84) | 10.98 (10.34-11.63) | 11.46 (10.65-12.27) | 8.87 (7.86-9.89) | 11.32 (10.16-12.48) | 11.90 (9.64-14.16) | **-2.47  (-2.76 to -2.18)** | **0.75  (0.25 to 1.25)** | **3.22 (2.64 to 3.80)** |
|  |  | Mid-high | 17.99 (17.33-18.64) | 15.53 (15.01-16.06) | 12.23 (11.79-12.67) | 9.67 (9.26-10.08) | 9.10 (8.34-9.86) | 9.67 (9.26-10.08) | 10.32 (9.56-11.07) | 11.95 (11.07-12.82) | **-2.17  (-2.35 to -1.99)** | **0.63  (0.31 to 0.96)** | **2.80 (2.43 to 3.17)** |
|  |  | Middle | 18.09 (17.57-18.61) | 15.22 (14.71-15.74) | 12.50 (12.06-12.93) | 9.50 (9.11-9.89) | 11.18 (10.63-11.73) | 9.20 (8.42-9.97) | 11.02 (10.17-11.86) | 13.12 (12.19-14.05) | **-2.17  (-2.35 to -1.99)** | **0.63  (0.31 to 0.96)** | **2.80 (2.43 to 3.17)** |
|  |  | Mid-low | 23.97 (22.90-25.04) | 21.12 (20.13-22.10) | 19.29 (18.33-20.26) | 15.44 (14.46-16.42) | 18.05 (16.48-19.61) | 16.49 (14.40-18.58) | 19.39 (16.65-22.13) | 19.81 (17.30-22.32) | **-1.97  (-2.36 to -1.59)** | 0.77  (-0.17 to 1.70) | **2.74 (1.73 to 3.75)** |
|  |  | low | 32.98 (30.64-35.31) | 29.16 (26.93-31.38) | 27.37 (25.02-29.72) | 24.58 (21.82-27.35) | 27.22 (23.16-31.29) | 22.07 (16.01-28.13) | 35.38 (28.65-42.12) | 31.61 (23.87-39.34) | **-1.96  (-2.89 to -1.03)** | 2.53  (-0.12 to 5.17) | **4.49 (1.69 to 7.29)** |
|  | Middle | High | 18.57 (16.59-20.56) | 16.71 (14.74-18.69) | 13.83 (12.37-15.28) | 10.67 (9.55-11.79) | 9.98 (8.67-11.28) | 6.52 (4.99-8.05) | 8.99 (7.18-10.80) | 18.53 (15.87-21.19) | **-2.3  (-2.82 to -1.78)** | 0.80  (-0.02 to 1.61) | **3.10 (2.13 to 4.07)** |
|  |  | Mid-high | 18.59 (17.75-19.43) | 15.29 (14.51-16.06) | 13.71 (13.08-14.33) | 10.08 (9.56-10.60) | 8.73 (7.75-9.72) | 10.08 (9.56-10.60) | 11.75 (10.71-12.79) | 11.64 (10.56-12.73) | **-2.00  (-2.24 to -1.77)** | **0.42  (0.01 to 0.83)** | **2.42 (1.95 to 2.89)** |
|  |  | Middle | 18.25 (17.64-18.85) | 15.88 (15.36-16.39) | 12.66 (12.25-13.07) | 9.42 (9.03-9.81) | 10.94 (10.44-11.44) | 8.86 (8.21-9.52) | 10.43 (9.75-11.11) | 11.12 (10.39-11.85) | **-2.00  (-2.24 to -1.77)** | **0.42  (0.01 to 0.83)** | **2.42 (1.95 to 2.89)** |
|  |  | Mid-low | 24.95 (23.85-26.04) | 22.82 (21.82-23.83) | 18.37 (17.45-19.29) | 15.45 (14.44-16.45) | 20.11 (18.52-21.71) | 15.83 (13.93-17.72) | 18.72 (16.36-21.08) | 20.40 (17.71-23.09) | **-2.06  (-2.45 to -1.66)** | 0.31  (-0.67 to 1.28) | **2.37 (1.32 to 3.42)** |
|  |  | low | 35.67 (33.22-38.12) | 30.80 (28.17-33.43) | 27.60 (25.04-30.16) | 25.43 (22.22-28.64) | 25.14 (20.62-29.65) | 18.58 (12.67-24.50) | 26.04 (18.93-33.16) | 34.84 (26.77-42.91) | **-2.97  (-3.99 to -1.95)** | **3.30  (0.39 to 6.21)** | **6.27 (3.19 to 9.35)** |
|  | High | High | 24.82 (22.89-26.75) | 23.20 (21.06-25.33) | 17.82 (16.39-19.26) | 13.12 (11.96-14.27) | 14.69 (13.14-16.25) | 10.37 (8.48-12.27) | 11.26 (9.22-13.30) | 18.53 (15.87-21.19) | **-3.04  (-3.59 to -2.48)** | **1.23  (0.26 to 2.21)** | **4.27 (3.15 to 5.39)** |
|  |  | Mid-high | 23.45 (22.44-24.46) | 21.91 (20.98-22.85) | 18.17 (17.41-18.93) | 13.54 (12.85-14.23) | 12.80 (11.46-14.15) | 13.54 (12.85-14.23) | 13.89 (12.50-15.28) | 15.41 (13.95-16.87) | **-2.66  (-2.95 to -2.36)** | 0.35  (-0.20 to 0.90) | **3.01 (2.39 to 3.63)** |
|  |  | Middle | 23.24 (22.61-23.87) | 19.62 (19.09-20.15) | 16.62 (16.18-17.06) | 12.53 (12.12-12.95) | 14.38 (13.81-14.95) | 11.95 (11.19-12.71) | 14.02 (13.16-14.88) | 15.51 (14.69-16.33) | **-2.66  (-2.95 to -2.36)** | 0.35  (-0.20 to 0.90) | **3.01 (2.39 to 3.63)** |
|  |  | Mid-low | 28.88 (27.98-29.79) | 25.46 (24.64-26.28) | 22.84 (22.12-23.56) | 18.54 (17.76-19.32) | 22.11 (20.96-23.26) | 17.71 (16.09-19.33) | 20.92 (19.22-22.62) | 24.36 (22.41-26.30) | **-2.31  (-2.62 to -2.01)** | **0.86  (0.16 to 1.57)** | **3.17 (2.40 to 3.94)** |
|  |  | low | 37.25 (35.95-38.56) | 34.80 (33.54-36.06) | 32.19 (30.89-33.49) | 27.72 (26.09-29.35) | 30.29 (27.70-32.87) | 26.22 (22.85-29.58) | 27.14 (23.61-30.67) | 31.07 (27.01-35.12) | **-2.35  (-2.90 to -1.80)** | 0.26  (-1.25 to 1.77) | **2.61 (1.00 to 4.22)** |
| **Suicidal attempts** | | | | | | | | | | | | | |
| Grade level | Middle school (7-9^th^) | High | 7.07 (6.43-7.71) | 6.37 (5.73-7.02) | 5.04 (4.51-5.57) | 3.04 (2.71-3.37) | 3.38 (2.96-3.79) | 1.49 (1.09-1.90) | 2.22 (1.71-2.73) | 2.94 (2.37-3.50) | 0.34 (-0.15 to 0.84) | -0.46 (-1.83 to 0.90) | -0.80 (-2.25 to 0.65) |
|  |  | Mid-high | 4.84 (4.53-5.14) | 4.23 (3.94-4.51) | 3.40 (3.16-3.63) | 2.38 (2.19-2.57) | 2.66 (2.40-2.92) | 1.74 (1.40-2.08) | 1.87 (1.55-2.18) | 2.24 (1.87-2.61) | -0.63 (-0.72 to -0.55) | -0.11 (-0.25 to 0.04) | **0.52 (0.35 to 0.69)** |
|  |  | Middle | 4.84 (4.53-5.14) | 4.23 (3.94-4.51) | 3.40 (3.16-3.63) | 2.38 (2.19-2.57) | 2.66 (2.40-2.92) | 1.74 (1.40-2.08) | 1.87 (1.55-2.18) | 2.24 (1.87-2.61) | -0.50 (-0.58 to -0.43) | -0.11 (-0.24 to 0.02) | **0.39 (0.24 to 0.54)** |
|  |  | Mid-low | 7.34 (6.81-7.86) | 7.04 (6.51-7.57) | 6.23 (5.77-6.69) | 4.74 (4.25-5.23) | 7.42 (6.60-8.24) | 4.29 (3.39-5.20) | 4.95 (3.96-5.94) | 5.09 (4.06-6.11) | -0.34 (-0.53 to -0.14) | **-0.64 (-1.05 to -0.22)** | -0.30 (-0.76 to 0.16) |
|  |  | low | 13.60 (12.53-14.67) | 13.15 (11.95-14.36) | 13.98 (12.54-15.41) | 10.75 (9.22-12.28) | 12.94 (10.57-15.32) | 8.08 (5.09-11.06) | 10.01 (7.04-12.98) | 13.69 (10.15-17.23) | -0.34 (-0.84 to 0.15) | 0.46 (-0.90 to 1.83) | 0.80 (-0.65 to 2.25) |
|  | High school (10-12^th^) | High | 7.18 (6.25-8.12) | 6.20 (5.36-7.04) | 4.88 (4.23-5.53) | 3.68 (3.16-4.19) | 3.21 (2.68-3.74) | 2.60 (1.88-3.32) | 2.44 (1.57-3.31) | 4.24 (3.22-5.25) | 0.70 (0.34 to 1.06) | 0.17 (-0.67 to 1.02) | -0.53 (-1.45 to 0.39) |
|  |  | Mid-high | 4.21 (3.83-4.59) | 3.24 (2.95-3.54) | 2.56 (2.32-2.80) | 1.68 (1.50-1.85) | 1.96 (1.72-2.21) | 1.82 (1.44-2.20) | 1.71 (1.37-2.04) | 2.00 (1.61-2.39) | -0.60 (-0.70 to -0.51) | 0.00 (-0.15 to 0.14) | **0.60 (0.43 to 0.77)** |
|  |  | Middle | 4.21 (3.83-4.59) | 3.24 (2.95-3.54) | 2.56 (2.32-2.80) | 1.68 (1.50-1.85) | 1.96 (1.72-2.21) | 1.82 (1.44-2.20) | 1.71 (1.37-2.04) | 2.00 (1.61-2.39) | -0.55 (-0.62 to -0.49) | -0.07 (-0.17 to 0.03) | **0.48 (0.36 to 0.60)** |
|  |  | Mid-low | 5.52 (5.08-5.95) | 4.36 (4.02-4.70) | 3.32 (3.05-3.60) | 2.67 (2.36-2.97) | 3.99 (3.51-4.48) | 2.89 (2.28-3.50) | 3.42 (2.62-4.22) | 2.81 (2.06-3.55) | -0.59 (-0.72 to -0.45) | -0.32 (-0.6 to -0.04) | 0.27 (-0.04 to 0.58) |
|  |  | low | 10.27 (9.32-11.22) | 10.08 (9.25-10.91) | 7.84 (7.13-8.55) | 7.54 (6.61-8.46) | 8.87 (7.32-10.42) | 5.41 (3.71-7.10) | 7.28 (5.38-9.17) | 8.09 (5.84-10.34) | -0.70 (-1.06 to -0.34) | -0.17 (-1.02 to 0.67) | 0.53 (-0.39 to 1.45) |
| Residential area | Rural | High | 6.29 (5.46-7.11) | 6.26 (5.39-7.14) | 5.47 (4.59-6.35) | 3.69 (3.14-4.24) | 3.05 (2.42-3.68) | 1.29 (0.71-1.88) | 2.30 (1.46-3.15) | 3.57 (2.72-4.42) | **0.44 (0.08 to 0.80)** | -0.16 (-1.10 to 0.79) | -0.60 (-1.61 to 0.41) |
|  |  | Mid-high | 4.27 (3.90-4.65) | 3.69 (3.34-4.03) | 2.90 (2.59-3.22) | 2.03 (1.79-2.28) | 2.15 (1.81-2.48) | 1.86 (1.41-2.31) | 1.70 (1.29-2.12) | 2.35 (1.81-2.89) | -0.65 (-0.73 to -0.58) | -0.10 (-0.22 to 0.02) | **0.55 (0.41 to 0.69)** |
|  |  | Middle | 4.27 (3.90-4.65) | 3.69 (3.34-4.03) | 2.90 (2.59-3.22) | 2.03 (1.79-2.28) | 2.15 (1.81-2.48) | 1.86 (1.41-2.31) | 1.70 (1.29-2.12) | 2.35 (1.81-2.89) | -0.53 (-0.59 to -0.47) | -0.08 (-0.18 to 0.02) | **0.45 (0.33 to 0.57)** |
|  |  | Mid-low | 6.01 (5.46-6.56) | 5.47 (5.01-5.92) | 4.33 (3.90-4.75) | 3.55 (3.07-4.04) | 5.19 (4.47-5.91) | 3.65 (2.69-4.61) | 3.83 (2.91-4.74) | 3.45 (2.46-4.45) | -0.60 (-0.74 to -0.46) | **-0.33 (-0.62 to -0.03)** | 0.27 (-0.06 to 0.60) |
|  |  | low | 12.52 (11.31-13.74) | 11.86 (10.81-12.92) | 9.41 (8.19-10.64) | 8.52 (7.20-9.83) | 8.34 (6.26-10.42) | 6.85 (4.46-9.23) | 6.31 (4.05-8.56) | 9.70 (6.54-12.85) | -0.44 (-0.80 to -0.08) | 0.16 (-0.79 to 1.10) | 0.60 (-0.41 to 1.61) |
|  | Urban | High | 7.42 (6.77-8.08) | 6.33 (5.70-6.95) | 4.79 (4.34-5.25) | 3.15 (2.81-3.48) | 3.40 (3.02-3.78) | 2.15 (1.68-2.61) | 2.31 (1.76-2.85) | 3.36 (2.72-4.00) | **1.26 (0.78 to 1.74)** | -0.25 (-1.41 to 0.92) | **-1.51 (-2.77 to -0.25)** |
|  |  | Mid-high | 4.71 (4.42-5.00) | 3.86 (3.61-4.12) | 3.08 (2.88-3.29) | 2.05 (1.89-2.20) | 2.40 (2.18-2.61) | 1.74 (1.44-2.05) | 1.83 (1.56-2.11) | 2.05 (1.74-2.36) | -0.6 (-0.71 to -0.49) | 0.05 (-0.15 to 0.25) | **0.65 (0.42 to 0.88)** |
|  |  | Middle | 4.71 (4.42-5.00) | 3.86 (3.61-4.12) | 3.08 (2.88-3.29) | 2.05 (1.89-2.20) | 2.40 (2.18-2.61) | 1.74 (1.44-2.05) | 1.83 (1.56-2.11) | 2.05 (1.74-2.36) | -0.6 (-0.69 to -0.52) | -0.06 (-0.21 to 0.08) | **0.54 (0.37 to 0.71)** |
|  |  | Mid-low | 6.42 (6.00-6.84) | 5.45 (5.06-5.83) | 4.42 (4.12-4.72) | 3.25 (2.94-3.56) | 5.22 (4.69-5.76) | 3.34 (2.73-3.95) | 4.12 (3.29-4.94) | 3.97 (3.19-4.74) | -0.46 (-0.64 to -0.27) | **-0.52 (-0.91 to -0.14)** | -0.06 (-0.49 to 0.37) |
|  |  | low | 11.19 (10.31-12.07) | 11.00 (10.11-11.89) | 10.10 (9.29-10.91) | 8.41 (7.42-9.40) | 11.02 (9.37-12.68) | 6.03 (4.08-7.97) | 9.32 (7.16-11.48) | 10.81 (8.28-13.34) | -1.26 (-1.74 to -0.78) | 0.25 (-0.92 to 1.41) | **1.51 (0.25 to 2.77)** |
| Recent alcohol consumption | No | High | 5.49 (4.96-6.01) | 4.75 (4.26-5.24) | 3.91 (3.53-4.30) | 2.52 (2.27-2.78) | 2.23 (1.93-2.53) | 1.34 (1.02-1.67) | 1.64 (1.27-2.02) | 2.47 (2.03-2.92) | **0.56 (0.27 to 0.84)** | -0.29 (-1.03 to 0.45) | **-0.85 (-1.64 to -0.06)** |
|  |  | Mid-high | 3.65 (3.41-3.88) | 3.09 (2.88-3.29) | 2.44 (2.28-2.61) | 1.77 (1.64-1.89) | 1.90 (1.72-2.08) | 1.48 (1.24-1.72) | 1.51 (1.29-1.74) | 1.76 (1.50-2.02) | -0.48 (-0.55 to -0.42) | -0.04 (-0.14 to 0.06) | **0.44 (0.32 to 0.56)** |
|  |  | Middle | 3.65 (3.41-3.88) | 3.09 (2.88-3.29) | 2.44 (2.28-2.61) | 1.77 (1.64-1.89) | 1.90 (1.72-2.08) | 1.48 (1.24-1.72) | 1.51 (1.29-1.74) | 1.76 (1.50-2.02) | -0.41 (-0.46 to -0.37) | -0.03 (-0.11 to 0.05) | **0.38 (0.29 to 0.47)** |
|  |  | Mid-low | 4.78 (4.48-5.09) | 4.24 (3.97-4.51) | 3.59 (3.35-3.83) | 2.84 (2.57-3.10) | 4.27 (3.85-4.69) | 2.78 (2.28-3.29) | 3.16 (2.56-3.76) | 3.21 (2.59-3.83) | **-0.32 (-0.43 to -0.22)** | **-0.30 (-0.53 to -0.06)** | 0.02 (-0.24 to 0.28) |
|  |  | low | 8.25 (7.52-8.97) | 8.28 (7.56-9.01) | 7.37 (6.63-8.10) | 6.14 (5.34-6.93) | 6.66 (5.41-7.91) | 4.44 (3.10-5.78) | 5.11 (3.59-6.62) | 7.58 (5.54-9.61) | **-0.56 (-0.84 to -0.27)** | 0.29 (-0.45 to 1.03) | **0.85 (0.06 to 1.64)** |
|  | Yes | High | 12.05 (10.76-13.34) | 12.08 (10.67-13.48) | 10.34 (8.97-11.71) | 7.59 (6.46-8.73) | 8.68 (7.42-9.94) | 6.86 (4.67-9.06) | 8.05 (5.46-10.65) | 9.66 (7.20-12.13) | **0.29 (-0.35 to 0.93)** | -0.58 (-2.50 to 1.34) | -0.87 (-2.89 to 1.15) |
|  |  | Mid-high | 7.60 (7.00-8.20) | 7.02 (6.38-7.66) | 6.36 (5.75-6.97) | 3.75 (3.29-4.21) | 4.91 (4.25-5.58) | 4.58 (3.28-5.89) | 4.51 (3.32-5.71) | 4.88 (3.83-5.93) | **-0.88 (-1.07 to -0.68)** | -0.02 (-0.42 to 0.38) | **0.86 (0.42 to 1.31)** |
|  |  | Middle | 7.60 (7.00-8.20) | 7.02 (6.38-7.66) | 6.36 (5.75-6.97) | 3.75 (3.29-4.21) | 4.91 (4.25-5.58) | 4.58 (3.28-5.89) | 4.51 (3.32-5.71) | 4.88 (3.83-5.93) | **-0.72 (-0.88 to -0.57)** | -0.20 (-0.52 to 0.12) | **0.52 (0.16 to 0.88)** |
|  |  | Mid-low | 9.64 (8.86-10.42) | 9.25 (8.46-10.04) | 7.31 (6.66-7.96) | 5.36 (4.60-6.13) | 8.90 (7.66-10.13) | 7.29 (5.34-9.24) | 8.78 (6.58-10.98) | 6.64 (4.67-8.61) | **-0.75 (-1.05 to -0.46)** | -0.56 (-1.29 to 0.18) | 0.19 (-0.60 to 0.98) |
|  |  | low | 17.36 (15.92-18.80) | 17.61 (16.20-19.02) | 15.73 (14.25-17.21) | 14.58 (12.62-16.54) | 18.84 (15.74-21.94) | 14.24 (9.18-19.30) | 20.05 (14.47-25.64) | 19.66 (14.37-24.95) | -0.29 (-0.93 to 0.35) | 0.58 (-1.34 to 2.50) | 0.87 (-1.15 to 2.89) |
| Smoking status | No | High | 6.04 (5.52-6.56) | 4.66 (4.22-5.09) | 3.83 (3.47-4.19) | 2.51 (2.26-2.75) | 2.53 (2.23-2.84) | 1.52 (1.18-1.86) | 1.88 (1.46-2.31) | 2.57 (2.11-3.03) | **0.55 (0.28 to 0.83)** | 0.05 (-0.63 to 0.73) | -0.50 (-1.23 to 0.23) |
|  |  | Mid-high | 3.93 (3.70-4.16) | 3.29 (3.08-3.49) | 2.58 (2.42-2.74) | 1.86 (1.73-1.99) | 2.10 (1.93-2.28) | 1.65 (1.40-1.90) | 1.69 (1.47-1.92) | 1.86 (1.60-2.11) | **-0.51 (-0.58 to -0.45)** | -0.07 (-0.17 to 0.03) | **0.44 (0.32 to 0.56)** |
|  |  | Middle | 3.93 (3.70-4.16) | 3.29 (3.08-3.49) | 2.58 (2.42-2.74) | 1.86 (1.73-1.99) | 2.10 (1.93-2.28) | 1.65 (1.40-1.90) | 1.69 (1.47-1.92) | 1.86 (1.60-2.11) | **-0.43 (-0.48 to -0.38)** | -0.07 (-0.15 to 0.01) | **0.36 (0.27 to 0.45)** |
|  |  | Mid-low | 5.27 (4.96-5.58) | 4.63 (4.34-4.93) | 3.67 (3.43-3.91) | 2.85 (2.60-3.09) | 4.76 (4.33-5.18) | 3.16 (2.67-3.66) | 3.51 (2.92-4.10) | 3.36 (2.77-3.95) | **-0.39 (-0.50 to -0.28)** | **-0.40 (-0.63 to -0.17)** | -0.01 (-0.26 to 0.24) |
|  |  | low | 8.68 (7.99-9.38) | 8.44 (7.76-9.12) | 7.06 (6.38-7.73) | 5.88 (5.15-6.61) | 7.86 (6.64-9.08) | 5.55 (4.09-7.00) | 6.42 (4.89-7.95) | 7.54 (5.72-9.37) | **-0.55 (-0.83 to -0.28)** | -0.05 (-0.73 to 0.63) | 0.50 (-0.23 to 1.23) |
|  | Yes | High | 14.15 (12.23-16.06) | 15.52 (13.47-17.57) | 13.68 (11.79-15.57) | 11.44 (9.76-13.11) | 13.26 (10.93-15.59) | 15.45 (9.19-21.71) | 12.14 (7.30-16.98) | 17.14 (12.32-21.95) | 0.15 (-0.72 to 1.02) | -2.46 (-5.42 to 0.50) | -2.61 (-5.70 to 0.48) |
|  |  | Mid-high | 10.11 (9.09-11.13) | 8.38 (7.47-9.28) | 7.72 (6.83-8.61) | 4.86 (4.05-5.68) | 6.89 (5.48-8.30) | 7.89 (4.74-11.05) | 5.54 (3.24-7.83) | 10.45 (7.34-13.57) | **-1.13 (-1.48 to -0.77)** | 0.87 (-0.16 to 1.90) | **2.00 (0.91 to 3.09)** |
|  |  | Middle | 10.11 (9.09-11.13) | 8.38 (7.47-9.28) | 7.72 (6.83-8.61) | 4.86 (4.05-5.68) | 6.89 (5.48-8.30) | 7.89 (4.74-11.05) | 5.54 (3.24-7.83) | 10.45 (7.34-13.57) | **-0.93 (-1.20 to -0.66)** | 0.33 (-0.44 to 1.10) | **1.26 (0.44 to 2.08)** |
|  |  | Mid-low | 11.87 (10.57-13.17) | 10.14 (9.13-11.16) | 8.90 (7.97-9.84) | 7.98 (6.67-9.29) | 10.81 (8.72-12.91) | 13.62 (7.68-19.55) | 14.23 (8.76-19.71) | 12.22 (7.61-16.82) | **-0.82 (-1.31 to -0.34)** | 0.70 (-0.87 to 2.27) | 1.52 (-0.12 to 3.16) |
|  |  | low | 20.55 (18.66-22.45) | 19.88 (18.15-21.61) | 18.74 (16.90-20.57) | 18.64 (16.03-21.24) | 23.16 (18.40-27.93) | 18.03 (8.27-27.80) | 26.87 (16.82-36.91) | 30.07 (22.16-37.97) | -0.15 (-1.02 to 0.72) | 2.46 (-0.50 to 5.42) | 2.61 (-0.48 to 5.70) |
| Parental educational attainment | High school diploma or less | High | 8.04 (7.08-9.00) | 8.10 (7.05-9.16) | 6.81 (5.93-7.69) | 4.56 (3.87-5.26) | 4.25 (3.62-4.88) | 2.30 (1.63-2.97) | 2.33 (1.70-2.97) | 4.68 (3.66-5.70) | **0.55 (0.20 to 0.89)** | -0.12 (-1.02 to 0.78) | -0.67 (-1.63 to 0.29) |
|  |  | Mid-high | 4.56 (4.20-4.91) | 4.15 (3.79-4.51) | 3.22 (2.92-3.52) | 2.14 (1.89-2.39) | 2.26 (1.96-2.56) | 1.69 (1.29-2.09) | 1.70 (1.34-2.06) | 2.35 (1.93-2.78) | **-0.65 (-0.76 to -0.55)** | 0.03 (-0.14 to 0.19) | **0.68 (0.48 to 0.88)** |
|  |  | Middle | 4.56 (4.20-4.91) | 4.15 (3.79-4.51) | 3.22 (2.92-3.52) | 2.14 (1.89-2.39) | 2.26 (1.96-2.56) | 1.69 (1.29-2.09) | 1.70 (1.34-2.06) | 2.35 (1.93-2.78) | **-0.51 (-0.58 to -0.44)** | -0.09 (-0.21 to 0.02) | **0.42 (0.29 to 0.55)** |
|  |  | Mid-low | 6.08 (5.71-6.46) | 5.41 (5.06-5.76) | 4.30 (3.99-4.60) | 3.48 (3.14-3.81) | 5.28 (4.73-5.83) | 3.00 (2.42-3.58) | 4.14 (3.35-4.94) | 4.02 (3.19-4.84) | **-0.48 (-0.62 to -0.35)** | -0.30 (-0.61 to 0.01) | 0.18 (-0.16 to 0.52) |
|  |  | low | 11.64 (10.84-12.44) | 11.07 (10.27-11.88) | 9.98 (9.20-10.76) | 9.16 (8.13-10.19) | 10.55 (8.93-12.17) | 7.22 (5.29-9.15) | 8.04 (6.14-9.93) | 10.99 (8.61-13.36) | **-0.55 (-0.89 to -0.20)** | 0.12 (-0.78 to 1.02) | 0.67 (-0.29 to 1.63) |
|  | Bachelor's degree or higher | High | 6.55 (5.92-7.18) | 5.57 (4.99-6.14) | 4.27 (3.83-4.71) | 2.85 (2.56-3.14) | 2.74 (2.35-3.12) | 1.64 (1.19-2.10) | 2.28 (1.66-2.91) | 2.68 (2.11-3.26) | **1.14 (0.61 to 1.68)** | -0.29 (-1.58 to 1.00) | **-1.43 (-2.83 to -0.03)** |
|  |  | Mid-high | 4.61 (4.29-4.93) | 3.64 (3.39-3.90) | 2.95 (2.75-3.15) | 2.01 (1.85-2.16) | 2.36 (2.13-2.59) | 1.84 (1.54-2.13) | 1.86 (1.56-2.15) | 2.02 (1.68-2.35) | **-0.63 (-0.71 to -0.54)** | -0.1 (-0.23 to 0.03) | **0.53 (0.37 to 0.69)** |
|  |  | Middle | 4.61 (4.29-4.93) | 3.64 (3.39-3.90) | 2.95 (2.75-3.15) | 2.01 (1.85-2.16) | 2.36 (2.13-2.59) | 1.84 (1.54-2.13) | 1.86 (1.56-2.15) | 2.02 (1.68-2.35) | **-0.60 (-0.67 to -0.54)** | -0.06 (-0.17 to 0.05) | **0.54 (0.41 to 0.67)** |
|  |  | Mid-low | 6.82 (6.13-7.51) | 5.54 (5.04-6.04) | 4.55 (4.16-4.95) | 3.17 (2.79-3.55) | 5.10 (4.40-5.80) | 4.16 (3.26-5.06) | 3.81 (2.80-4.82) | 3.47 (2.59-4.35) | **-0.70 (-0.90 to -0.50)** | **-0.53 (-0.89 to -0.17)** | 0.17 (-0.24 to 0.58) |
|  |  | low | 11.67 (10.09-13.24) | 11.84 (10.45-13.24) | 9.60 (8.37-10.84) | 6.90 (5.68-8.11) | 9.14 (7.06-11.22) | 4.06 (2.10-6.01) | 8.80 (5.59-12.00) | 9.06 (5.42-12.71) | **-1.14 (-1.68 to -0.61)** | 0.29 (-1.00 to 1.58) | **1.43 (0.03 to 2.83)** |
| Academic achievement | Low | High | 6.83 (6.17-7.48) | 5.88 (5.32-6.44) | 4.93 (4.39-5.46) | 3.48 (3.09-3.86) | 3.23 (2.81-3.64) | 1.83 (1.35-2.31) | 2.45 (1.82-3.09) | 2.96 (2.34-3.59) | 0.09 (-0.51 to 0.70) | 0.8 (-0.66 to 2.26) | 0.71 (-0.87 to 2.29) |
|  |  | Mid-high | 3.75 (3.46-4.04) | 2.90 (2.65-3.15) | 2.44 (2.23-2.65) | 1.71 (1.54-1.87) | 1.74 (1.51-1.96) | 1.33 (1.03-1.63) | 1.37 (1.08-1.66) | 1.90 (1.57-2.23) | **0.53 (0.45 to 0.61)** | -0.05 (-0.18 to 0.07) | **-0.58 (-0.73 to -0.43)** |
|  |  | Middle | 3.75 (3.46-4.04) | 2.90 (2.65-3.15) | 2.44 (2.23-2.65) | 1.71 (1.54-1.87) | 1.74 (1.51-1.96) | 1.33 (1.03-1.63) | 1.37 (1.08-1.66) | 1.90 (1.57-2.23) | **0.43 (0.35 to 0.50)** | 0.1 (-0.04 to 0.23) | **-0.33 (-0.48 to -0.18)** |
|  |  | Mid-low | 4.94 (4.37-5.50) | 4.25 (3.72-4.79) | 3.38 (2.96-3.80) | 2.97 (2.51-3.43) | 4.32 (3.50-5.15) | 2.85 (1.86-3.83) | 3.68 (2.47-4.88) | 2.92 (1.82-4.02) | **0.37 (0.16 to 0.57)** | 0.36 (-0.07 to 0.80) | -0.01 (-0.49 to 0.47) |
|  |  | low | 9.48 (8.02-10.93) | 7.50 (6.22-8.78) | 7.24 (5.89-8.60) | 7.69 (5.96-9.41) | 10.43 (7.61-13.24) | 6.88 (2.96-10.81) | 9.54 (6.08-13.00) | 7.07 (3.36-10.78) | 0.09 (-0.51 to 0.70) | 0.8 (-0.66 to 2.26) | 0.71 (-0.87 to 2.29) |
|  | Middle | High | 5.10 (4.00-6.21) | 4.79 (3.55-6.04) | 3.63 (2.81-4.45) | 2.04 (1.57-2.52) | 2.55 (1.89-3.21) | 1.43 (0.71-2.15) | 1.50 (0.73-2.27) | 3.35 (2.19-4.51) | 0.52 (-0.13 to 1.17) | -0.02 (-1.93 to 1.89) | -0.54 (-2.56 to 1.48) |
|  |  | Mid-high | 4.46 (4.04-4.89) | 3.45 (3.05-3.84) | 2.71 (2.42-2.99) | 1.85 (1.61-2.09) | 2.44 (2.10-2.78) | 1.63 (1.19-2.06) | 1.89 (1.46-2.31) | 2.01 (1.56-2.46) | **0.57 (0.45 to 0.68)** | 0.11 (-0.07 to 0.28) | **-0.46 (-0.67 to -0.25)** |
|  |  | Middle | 4.46 (4.04-4.89) | 3.45 (3.05-3.84) | 2.71 (2.42-2.99) | 1.85 (1.61-2.09) | 2.44 (2.10-2.78) | 1.63 (1.19-2.06) | 1.89 (1.46-2.31) | 2.01 (1.56-2.46) | **0.49 (0.41 to 0.58)** | 0.06 (-0.06 to 0.18) | **-0.43 (-0.58 to -0.28)** |
|  |  | Mid-low | 5.83 (5.14-6.53) | 4.93 (4.42-5.44) | 3.68 (3.24-4.11) | 2.98 (2.52-3.44) | 4.19 (3.40-4.98) | 2.90 (2.05-3.75) | 3.54 (2.45-4.63) | 3.65 (2.51-4.79) | **0.63 (0.41 to 0.85)** | 0.11 (-0.32 to 0.55) | **-0.52 (-1.01 to -0.03)** |
|  |  | low | 9.48 (7.97-10.99) | 8.39 (6.88-9.90) | 6.98 (5.60-8.36) | 5.39 (3.84-6.94) | 10.15 (6.92-13.39) | 3.95 (1.33-6.57) | 6.35 (2.44-10.25) | 10.21 (4.89-15.54) | 0.52 (-0.13 to 1.17) | -0.02 (-1.93 to 1.89) | -0.54 (-2.56 to 1.48) |
|  | High | High | 9.39 (8.10-10.68) | 8.89 (7.31-10.47) | 6.14 (5.28-7.01) | 3.85 (3.20-4.50) | 4.32 (3.43-5.20) | 2.58 (1.68-3.49) | 2.62 (1.72-3.51) | 5.08 (3.70-6.45) | **0.94 (0.57 to 1.31)** | -0.51 (-1.45 to 0.44) | **-1.45 (-2.46 to -0.44)** |
|  |  | Mid-high | 6.70 (6.12-7.29) | 6.18 (5.65-6.70) | 4.66 (4.24-5.09) | 3.06 (2.72-3.40) | 3.57 (3.09-4.04) | 2.96 (2.27-3.65) | 2.73 (2.09-3.36) | 2.88 (2.26-3.51) | **0.96 (0.80 to 1.12)** | 0.23 (-0.03 to 0.48) | **-0.73 (-1.03 to -0.43)** |
|  |  | Middle | 6.70 (6.12-7.29) | 6.18 (5.65-6.70) | 4.66 (4.24-5.09) | 3.06 (2.72-3.40) | 3.57 (3.09-4.04) | 2.96 (2.27-3.65) | 2.73 (2.09-3.36) | 2.88 (2.26-3.51) | **0.71 (0.62 to 0.79)** | 0.09 (-0.07 to 0.24) | **-0.62 (-0.80 to -0.44)** |
|  |  | Mid-low | 7.25 (6.76-7.74) | 6.29 (5.85-6.73) | 5.20 (4.83-5.57) | 3.71 (3.32-4.11) | 6.13 (5.49-6.77) | 3.98 (3.17-4.79) | 4.38 (3.43-5.33) | 4.29 (3.38-5.19) | **0.62 (0.46 to 0.79)** | **0.55 (0.20 to 0.90)** | -0.07 (-0.46 to 0.32) |
|  |  | low | 12.87 (11.94-13.79) | 12.89 (12.01-13.78) | 11.24 (10.39-12.09) | 9.48 (8.43-10.53) | 10.01 (8.36-11.66) | 6.75 (4.85-8.65) | 8.31 (6.16-10.45) | 11.36 (8.83-13.89) | **0.94 (0.57 to 1.31)** | -0.51 (-1.45 to 0.44) | **-1.45 (-2.46 to -0.44)** |

Abbreviations: CI, Confidence Interval; KYRBS, the Korea Youth Risk Behavior Survey.
Numbers in bold indicate a significant difference (p< 0.05).

**Table S4.** Comparative analysis of adolescents’ mental health indicators with various influencing factors: wOR approach based on KYRBS, 2006–2022

| Variables | | | Perceived stress level | | | | | | Sadness | | | | | | Suicidal ideation | | | | | | Suicidal attempt | | | | | |
| --- | --- | --- | --- | --- | --- | --- | --- | --- | --- | --- | --- | --- | --- | --- | --- | --- | --- | --- | --- | --- | --- | --- | --- | --- | --- | --- |
|  |  |  | 2020 vs 2019 | | 2021 vs 2020 | | 2022 vs 2021 | | 2020 vs 2019 | | 2021 vs 2020 | | 2022 vs 2021 | | 2020 vs 2019 | | 2021 vs 2020 | | 2022 vs 2021 | | 2020 vs 2019 | | 2021 vs 2020 | | 2022 vs 2021 | |
|  |  |  | wOR (95% CI) | p-value | wOR (95% CI) | p-value | wOR (95% CI) | p-value | wOR (95% CI) | p-value | wOR (95% CI) | p-value | wOR (95% CI) | p-value | wOR (95% CI) | p-value | wOR (95% CI) | p-value | wOR (95% CI) | p-value | wOR (95% CI) | p-value | wOR (95% CI) | p-value | wOR (95% CI) | p-value |
| Grade level | Middle school (7-9^th^) | High | **0.72 (0.64-0.82)** | **<0.001** | **1.61 (1.42-1.83)** | **<0.001** | **1.13 (1.01-1.27)** | **0.031** | **0.78 (0.69-0.88)** | **<0.001** | **1.42 (1.26-1.60)** | **<0.001** | 1.05 (0.94-1.18) | 0.404 | **0.70 (0.59-0.83)** | **<0.001** | **1.49 (1.25-1.77)** | **<0.001** | **1.29 (1.09-1.53)** | **0.003** | **0.43 (0.31-0.59)** | **<0.001** | **1.50 (1.04-2.15)** | **0.029** | 1.33 (0.98-1.81) | 0.067 |
|  |  | Mid-high | **0.75 (0.69-0.81)** | **<0.001** | **1.34 (1.24-1.45)** | **<0.001** | **1.14 (1.05-1.22)** | **<0.001** | **0.82 (0.76-0.90)** | **<0.001** | **1.21 (1.11-1.31)** | **<0.001** | **1.10 (1.01-1.18)** | **0.022** | **0.73 (0.65-0.82)** | **<0.001** | **1.34 (1.20-1.50)** | **<0.001** | **1.17 (1.05-1.30)** | **0.004** | **0.62 (0.49-0.80)** | **<0.001** | 1.08 (0.83-1.40) | 0.586 | 1.21 (0.95-1.53) | 0.129 |
|  |  | Middle | **0.72 (0.67-0.77)** | **<0.001** | **1.28 (1.20-1.37)** | **<0.001** | **1.17 (1.10-1.25)** | **<0.001** | **0.81 (0.76-0.87)** | **<0.001** | **1.13 (1.05-1.21)** | **0.001** | **1.14 (1.06-1.22)** | **<0.001** | **0.67 (0.60-0.73)** | **<0.001** | **1.41 (1.28-1.55)** | **<0.001** | **1.16 (1.06-1.27)** | **0.001** | **0.55 (0.46-0.66)** | **<0.001** | **1.24 (1.02-1.50)** | **0.031** | **1.35 (1.13-1.62)** | **0.001** |
|  |  | Mid-low | **0.77 (0.68-0.88)** | **<0.001** | **1.17 (1.03-1.33)** | **0.016** | 1.13 (0.99-1.28) | 0.076 | **0.81 (0.70-0.93)** | **0.003** | 1.15 (1.00-1.32) | 0.052 | **1.18 (1.03-1.36)** | **0.020** | **0.69 (0.59-0.81)** | **<0.001** | **1.25 (1.06-1.47)** | **0.008** | 1.14 (0.96-1.34) | 0.134 | **0.54 (0.41-0.71)** | **<0.001** | 1.16 (0.86-1.57) | 0.339 | 1.03 (0.76-1.39) | 0.847 |
|  |  | low | **0.68 (0.51-0.91)** | **0.009** | 1.29 (0.96-1.75) | 0.095 | 1.35 (0.99-1.84) | 0.062 | **0.67 (0.50-0.89)** | **0.006** | 1.29 (0.95-1.74) | 0.103 | 1.05 (0.77-1.43) | 0.749 | **0.59 (0.43-0.82)** | **0.001** | 1.40 (1.00-1.94) | 0.047 | 1.14 (0.83-1.56) | 0.417 | **0.50 (0.31-0.82)** | **0.005** | 1.27 (0.75-2.13) | 0.373 | 1.43 (0.91-2.23) | 0.118 |
|  | High school (10-12^th^) | High | **0.82 (0.71-0.95)** | **0.007** | **1.27 (1.10-1.46)** | **0.001** | **1.21 (1.05-1.38)** | **0.006** | 0.97 (0.84-1.13) | 0.727 | 0.96 (0.82-1.12) | 0.593 | **1.25 (1.07-1.46)** | **0.004** | 0.84 (0.68-1.05) | 0.125 | 1.02 (0.82-1.28) | 0.841 | **1.39 (1.13-1.70)** | **0.002** | 0.89 (0.61-1.30) | 0.555 | 0.94 (0.59-1.49) | 0.780 | **1.77 (1.14-2.75)** | **0.011** |
|  |  | Mid-high | **0.83 (0.76-0.91)** | **<0.001** | **1.16 (1.06-1.26)** | **<0.001** | 1.05 (0.97-1.15) | 0.209 | **0.88 (0.80-0.96)** | **0.004** | 0.99 (0.90-1.08) | 0.783 | **1.11 (1.02-1.21)** | **0.022** | 1.00 (0.87-1.14) | 0.944 | 1.04 (0.90-1.19) | 0.618 | 1.04 (0.92-1.17) | 0.58 | 1.00 (0.75-1.33) | 0.972 | 0.94 (0.70-1.25) | 0.661 | 1.18 (0.89-1.56) | 0.257 |
|  |  | Middle | **0.83 (0.77-0.89)** | **<0.001** | **1.15 (1.07-1.23)** | **<0.001** | 1.07 (1.00-1.15) | 0.064 | 0.93 (0.86-1.00) | 0.050 | 1.04 (0.96-1.12) | 0.362 | 1.07 (0.99-1.14) | 0.079 | 0.98 (0.89-1.08) | 0.667 | 1.03 (0.93-1.15) | 0.559 | **1.11 (1.01-1.23)** | **0.039** | 0.93 (0.76-1.14) | 0.474 | 0.96 (0.78-1.19) | 0.709 | 1.07 (0.86-1.33) | 0.569 |
|  |  | Mid-low | **0.81 (0.72-0.90)** | **<0.001** | **1.20 (1.06-1.35)** | **0.003** | 1.05 (0.93-1.19) | 0.422 | **0.86 (0.76-0.96)** | **0.010** | 1.08 (0.95-1.21) | 0.233 | 1.05 (0.92-1.19) | 0.469 | **0.84 (0.72-0.98)** | **0.022** | **1.22 (1.04-1.43)** | **0.014** | 1.12 (0.96-1.31) | 0.145 | **0.71 (0.54-0.95)** | **0.019** | 1.19 (0.86-1.65) | 0.294 | 0.82 (0.57-1.18) | 0.274 |
|  |  | low | 0.89 (0.70-1.12) | 0.324 | 1.00 (0.79-1.25) | 0.966 | **1.40 (1.09-1.80)** | **0.009** | 0.92 (0.75-1.13) | 0.414 | 1.00 (0.79-1.25) | 0.972 | 1.09 (0.85-1.41) | 0.500 | 0.82 (0.63-1.06) | 0.133 | 1.18 (0.91-1.53) | 0.227 | 1.12 (0.85-1.48) | 0.433 | **0.61 (0.40-0.93)** | **0.021** | 1.37 (0.89-2.12) | 0.152 | 1.12 (0.74-1.69) | 0.585 |
| Residential area | Rural | High | **0.80 (0.68-0.94)** | **0.006** | **1.31 (1.13-1.53)** | **<0.001** | **1.25 (1.08-1.46)** | **0.004** | 0.89 (0.75-1.04) | 0.149 | **1.20 (1.01-1.42)** | **0.037** | 1.13 (0.97-1.33) | 0.124 | **0.75 (0.59-0.96)** | **0.024** | **1.29 (1.02-1.63)** | **0.034** | **1.31 (1.06-1.61)** | **0.011** | **0.42 (0.24-0.74)** | **0.002** | 1.80 (0.99-3.25) | 0.052 | 1.57 (1.00-2.46) | 0.049 |
|  |  | Mid-high | **0.79 (0.70-0.88)** | **<0.001** | **1.24 (1.11-1.39)** | **<0.001** | 1.07 (0.96-1.18) | 0.220 | **0.81 (0.72-0.91)** | **<0.001** | **1.13 (1.01-1.26)** | **0.027** | 1.11 (1.00-1.24) | 0.057 | 0.91 (0.77-1.07) | 0.249 | 1.16 (1.00-1.35) | 0.058 | 1.03 (0.89-1.20) | 0.692 | 0.81 (0.59-1.11) | 0.190 | 0.91 (0.65-1.30) | 0.614 | 1.39 (0.99-1.95) | 0.059 |
|  |  | Middle | **0.76 (0.70-0.83)** | **<0.001** | **1.20 (1.10-1.30)** | **<0.001** | 1.08 (0.99-1.18) | 0.078 | 0.91 (0.83-1.00) | 0.043 | 1.07 (0.97-1.17) | 0.164 | 1.06 (0.97-1.16) | 0.213 | **0.88 (0.78-0.99)** | **0.030** | **1.19 (1.06-1.34)** | **0.005** | 1.03 (0.92-1.15) | 0.670 | **0.72 (0.57-0.92)** | **0.008** | **1.30 (1.02-1.65)** | **0.031** | 0.96 (0.76-1.21) | 0.732 |
|  |  | Mid-low | **0.85 (0.73-0.98)** | **0.027** | **1.21 (1.05-1.41)** | **0.010** | 0.97 (0.84-1.13) | 0.707 | **0.86 (0.74-0.99)** | **0.041** | **1.18 (1.02-1.36)** | **0.030** | 0.91 (0.77-1.06) | 0.226 | **0.78 (0.65-0.95)** | **0.013** | **1.32 (1.10-1.58)** | **0.003** | 0.95 (0.79-1.14) | 0.579 | **0.68 (0.48-0.96)** | **0.029** | 1.05 (0.73-1.52) | 0.795 | 0.90 (0.61-1.33) | 0.593 |
|  |  | low | 0.94 (0.68-1.31) | 0.729 | 1.08 (0.80-1.46) | 0.606 | 1.13 (0.83-1.54) | 0.437 | 0.95 (0.71-1.26) | 0.706 | 1.12 (0.84-1.49) | 0.433 | 0.99 (0.72-1.35) | 0.925 | 0.83 (0.59-1.18) | 0.304 | 1.18 (0.85-1.63) | 0.325 | 1.00 (0.71-1.41) | 1.000 | 0.88 (0.53-1.46) | 0.609 | 0.92 (0.54-1.56) | 0.746 | 1.60 (0.94-2.70) | 0.081 |
|  | Urban | High | **0.74 (0.66-0.83)** | **<0.001** | **1.53 (1.36-1.72)** | **<0.001** | **1.12 (1.01-1.24)** | **0.031** | **0.83 (0.75-0.93)** | **0.002** | **1.22 (1.09-1.37)** | **<0.001** | **1.12 (1.00-1.25)** | **0.050** | **0.75 (0.64-0.89)** | **0.001** | **1.28 (1.07-1.51)** | **0.005** | **1.33 (1.14-1.56)** | **<0.001** | **0.64 (0.49-0.84)** | **0.002** | 1.08 (0.78-1.49) | 0.658 | **1.47 (1.08-2.01)** | **0.014** |
|  |  | Mid-high | **0.78 (0.73-0.84)** | **<0.001** | **1.24 (1.16-1.33)** | **<0.001** | **1.11 (1.04-1.18)** | **0.002** | **0.86 (0.80-0.93)** | **<0.001** | 1.08 (1.00-1.16) | 0.039 | **1.10 (1.02-1.18)** | **0.008** | **0.82 (0.74-0.91)** | **<0.001** | **1.20 (1.08-1.33)** | **0.001** | **1.14 (1.04-1.26)** | **0.006** | **0.74 (0.59-0.93)** | **0.011** | 1.05 (0.83-1.33) | 0.668 | 1.12 (0.90-1.39) | 0.300 |
|  |  | Middle | **0.78 (0.74-0.83)** | **<0.001** | **1.21 (1.14-1.28)** | **<0.001** | **1.14 (1.07-1.20)** | **<0.001** | **0.85 (0.80-0.91)** | **<0.001** | **1.08 (1.01-1.15)** | **0.024** | **1.12 (1.06-1.19)** | **<0.001** | **0.79 (0.72-0.86)** | **<0.001** | **1.21 (1.11-1.32)** | **<0.001** | **1.19 (1.09-1.29)** | **<0.001** | **0.69 (0.59-0.82)** | **<0.001** | 1.01 (0.84-1.21) | 0.933 | **1.38 (1.16-1.64)** | **<0.001** |
|  |  | Mid-low | **0.77 (0.69-0.85)** | **<0.001** | **1.17 (1.05-1.30)** | **0.004** | **1.15 (1.03-1.29)** | **0.015** | **0.83 (0.74-0.93)** | **0.001** | 1.07 (0.95-1.19) | 0.286 | **1.24 (1.10-1.39)** | **<0.001** | **0.77 (0.68-0.89)** | **<0.001** | **1.18 (1.02-1.37)** | **0.025** | **1.26 (1.09-1.45)** | **0.002** | **0.60 (0.47-0.77)** | **<0.001** | 1.24 (0.94-1.64) | 0.129 | 0.96 (0.72-1.29) | 0.797 |
|  |  | low | **0.75 (0.60-0.94)** | **0.012** | 1.09 (0.86-1.37) | 0.483 | **1.54 (1.19-1.97)** | **<0.001** | **0.77 (0.63-0.95)** | **0.015** | 1.07 (0.85-1.36) | 0.547 | 1.12 (0.88-1.44) | 0.357 | **0.70 (0.54-0.89)** | **0.004** | 1.30 (1.00-1.69) | 0.049 | 1.23 (0.95-1.60) | 0.119 | **0.47 (0.31-0.71)** | **<0.001** | **1.60 (1.05-2.46)** | **0.031** | 1.18 (0.82-1.70) | 0.377 |
| Recent alcohol consumption | No | High | **0.78 (0.71-0.87)** | **<0.001** | **1.48 (1.34-1.65)** | **<0.001** | **1.15 (1.05-1.27)** | **0.003** | 0.95 (0.86-1.06) | 0.336 | **1.21 (1.09-1.35)** | **<0.001** | 1.09 (0.99-1.21) | 0.087 | **0.83 (0.71-0.97)** | **0.018** | **1.27 (1.09-1.49)** | **0.003** | **1.30 (1.13-1.51)** | **<0.001** | **0.58 (0.43-0.79)** | **<0.001** | 1.23 (0.88-1.72) | 0.232 | **1.52 (1.13-2.04)** | **0.006** |
|  |  | Mid-high | **0.79 (0.74-0.85)** | **<0.001** | **1.27 (1.19-1.35)** | **<0.001** | **1.10 (1.03-1.16)** | **0.002** | **0.86 (0.80-0.92)** | **<0.001** | **1.10 (1.03-1.17)** | **0.003** | **1.09 (1.02-1.16)** | **0.007** | **0.84 (0.76-0.92)** | **<0.001** | **1.25 (1.14-1.37)** | **<0.001** | 1.08 (0.99-1.18) | 0.097 | **0.76 (0.61-0.94)** | **0.012** | 1.02 (0.82-1.28) | 0.845 | 1.17 (0.94-1.44) | 0.155 |
|  |  | Middle | **0.77 (0.74-0.81)** | **<0.001** | **1.23 (1.17-1.29)** | **<0.001** | **1.11 (1.05-1.16)** | **<0.001** | **0.89 (0.84-0.94)** | **<0.001** | **1.09 (1.03-1.15)** | **0.003** | **1.10 (1.04-1.16)** | **<0.001** | **0.81 (0.75-0.87)** | **<0.001** | **1.25 (1.16-1.35)** | **<0.001** | **1.11 (1.04-1.20)** | **0.003** | **0.75 (0.64-0.88)** | **<0.001** | 1.17 (0.99-1.37) | 0.060 | 1.14 (0.97-1.34) | 0.104 |
|  |  | Mid-low | **0.78 (0.71-0.85)** | **<0.001** | **1.20 (1.09-1.32)** | **<0.001** | **1.11 (1.01-1.22)** | **0.032** | **0.87 (0.79-0.96)** | **0.005** | 1.08 (0.98-1.19) | 0.144 | **1.15 (1.04-1.28)** | **0.008** | **0.80 (0.71-0.91)** | **0.001** | **1.21 (1.07-1.37)** | **0.002** | **1.18 (1.04-1.34)** | **0.013** | **0.65 (0.51-0.82)** | **<0.001** | 1.14 (0.87-1.49) | 0.339 | 1.02 (0.77-1.34) | 0.911 |
|  |  | low | 0.85 (0.69-1.04) | 0.115 | 1.11 (0.90-1.35) | 0.327 | **1.38 (1.10-1.72)** | **0.005** | 0.90 (0.74-1.10) | 0.308 | 1.09 (0.90-1.34) | 0.376 | 1.02 (0.82-1.27) | 0.886 | 0.79 (0.62-1.00) | 0.046 | 1.16 (0.91-1.47) | 0.228 | 1.26 (0.99-1.61) | 0.06 | **0.62 (0.40-0.94)** | **0.026** | 1.16 (0.74-1.81) | 0.516 | 1.52 (0.99-2.33) | 0.053 |
|  | Yes | High | **0.74 (0.59-0.93)** | **0.009** | **1.34 (1.04-1.71)** | **0.023** | 1.09 (0.85-1.39) | 0.497 | **0.69 (0.55-0.87)** | **0.002** | 1.23 (0.95-1.58) | 0.121 | 1.14 (0.90-1.45) | 0.276 | **0.73 (0.55-0.97)** | **0.032** | 1.32 (0.97-1.79) | 0.079 | 1.23 (0.94-1.62) | 0.137 | 0.84 (0.55-1.27) | 0.399 | 1.19 (0.73-1.94) | 0.489 | 1.22 (0.78-1.92) | 0.384 |
|  |  | Mid-high | **0.82 (0.71-0.96)** | **0.011** | 1.08 (0.92-1.27) | 0.337 | 1.04 (0.90-1.21) | 0.617 | 0.94 (0.81-1.09) | 0.420 | 1.05 (0.89-1.23) | 0.566 | 1.02 (0.88-1.18) | 0.772 | 1.05 (0.87-1.26) | 0.644 | 0.94 (0.76-1.16) | 0.579 | 1.16 (0.96-1.41) | 0.131 | 0.96 (0.66-1.40) | 0.837 | 0.98 (0.66-1.48) | 0.938 | 1.08 (0.76-1.55) | 0.657 |
|  |  | Middle | 0.90 (0.80-1.01) | 0.068 | 1.00 (0.88-1.14) | 0.946 | **1.14 (1.01-1.30)** | **0.038** | 0.99 (0.88-1.12) | 0.906 | 0.99 (0.87-1.13) | 0.920 | 1.01 (0.89-1.14) | 0.882 | 1.01 (0.86-1.17) | 0.94 | 0.98 (0.83-1.17) | 0.83 | 1.14 (0.98-1.34) | 0.099 | **0.75 (0.57-0.97)** | **0.030** | 0.89 (0.66-1.21) | 0.464 | 1.34 (1.00-1.79) | 0.047 |
|  |  | Mid-low | 0.95 (0.77-1.17) | 0.641 | 1.10 (0.88-1.38) | 0.418 | 0.94 (0.75-1.18) | 0.583 | 0.85 (0.70-1.04) | 0.107 | 1.23 (0.98-1.53) | 0.073 | 0.88 (0.71-1.10) | 0.273 | **0.78 (0.62-0.98)** | **0.035** | 1.29 (1.00-1.66) | 0.048 | 0.97 (0.76-1.25) | 0.824 | 0.69 (0.48-1.00) | 0.048 | 1.22 (0.82-1.82) | 0.319 | 0.74 (0.49-1.12) | 0.158 |
|  |  | low | 0.85 (0.58-1.24) | 0.388 | 0.98 (0.65-1.47) | 0.910 | 1.35 (0.89-2.06) | 0.156 | 0.78 (0.54-1.12) | 0.182 | 1.01 (0.68-1.51) | 0.955 | 1.26 (0.84-1.90) | 0.270 | 0.78 (0.53-1.13) | 0.182 | **1.53 (1.01-2.31)** | **0.045** | 0.85 (0.56-1.28) | 0.429 | 0.67 (0.40-1.10) | 0.115 | 1.51 (0.88-2.60) | 0.135 | 0.98 (0.60-1.58) | 0.919 |
| Smoking status | No | High | **0.76 (0.68-0.83)** | **<0.001** | **1.49 (1.35-1.65)** | **<0.001** | **1.14 (1.05-1.25)** | **0.003** | **0.87 (0.79-0.96)** | **0.005** | **1.21 (1.09-1.33)** | **<0.001** | 1.10 (1.00-1.20) | 0.059 | **0.79 (0.68-0.91)** | **0.001** | **1.27 (1.10-1.46)** | **0.001** | **1.26 (1.10-1.45)** | **0.001** | **0.58 (0.44-0.77)** | **<0.001** | 1.24 (0.90-1.71) | 0.190 | **1.38 (1.03-1.85)** | **0.033** |
|  |  | Mid-high | **0.78 (0.74-0.83)** | **<0.001** | **1.24 (1.17-1.31)** | **<0.001** | **1.10 (1.04-1.16)** | **0.001** | **0.85 (0.80-0.91)** | **<0.001** | **1.09 (1.02-1.16)** | **0.008** | **1.09 (1.02-1.15)** | **0.007** | **0.85 (0.78-0.93)** | **0.001** | **1.18 (1.08-1.29)** | **<0.001** | **1.09 (1.01-1.19)** | **0.039** | **0.78 (0.64-0.94)** | **0.011** | 1.03 (0.84-1.26) | 0.798 | 1.10 (0.90-1.34) | 0.354 |
|  |  | Middle | **0.78 (0.74-0.82)** | **<0.001** | **1.20 (1.14-1.26)** | **<0.001** | **1.11 (1.06-1.17)** | **<0.001** | **0.87 (0.83-0.92)** | **<0.001** | **1.07 (1.02-1.13)** | **0.011** | **1.09 (1.04-1.15)** | **<0.001** | **0.81 (0.75-0.87)** | **<0.001** | **1.21 (1.12-1.30)** | **<0.001** | **1.12 (1.05-1.20)** | **0.001** | **0.73 (0.63-0.83)** | **<0.001** | 1.10 (0.95-1.27) | 0.222 | **1.17 (1.01-1.36)** | **0.040** |
|  |  | Mid-low | **0.79 (0.72-0.86)** | **<0.001** | **1.17 (1.07-1.28)** | **<0.001** | 1.10 (1.00-1.21) | 0.040 | **0.84 (0.76-0.92)** | **<0.001** | 1.08 (0.98-1.18) | 0.126 | **1.12 (1.01-1.23)** | **0.029** | **0.79 (0.71-0.89)** | **<0.001** | **1.19 (1.06-1.33)** | **0.004** | **1.16 (1.03-1.30)** | **0.015** | **0.64 (0.52-0.79)** | **<0.001** | 1.11 (0.88-1.41) | 0.374 | 0.96 (0.74-1.23) | 0.723 |
|  |  | low | **0.82 (0.67-0.99)** | **0.038** | 1.11 (0.92-1.34) | 0.269 | **1.35 (1.10-1.67)** | **0.005** | 0.85 (0.71-1.01) | 0.071 | 1.04 (0.87-1.25) | 0.662 | 1.05 (0.86-1.29) | 0.646 | **0.75 (0.61-0.93)** | **0.008** | 1.23 (0.99-1.53) | 0.057 | 1.14 (0.91-1.42) | 0.270 | **0.63 (0.44-0.90)** | **0.012** | 1.17 (0.80-1.70) | 0.416 | 1.19 (0.83-1.71) | 0.353 |
|  | Yes | High | 1.14 (0.76-1.73) | 0.529 | 0.75 (0.48-1.19) | 0.219 | 1.31 (0.87-1.97) | 0.200 | 1.00 (0.66-1.51) | 0.998 | 0.98 (0.62-1.56) | 0.944 | 1.24 (0.84-1.83) | 0.272 | 0.87 (0.55-1.37) | 0.539 | 1.04 (0.63-1.72) | 0.870 | **1.60 (1.06-2.41)** | **0.027** | 1.09 (0.62-1.94) | 0.759 | 0.76 (0.39-1.46) | 0.406 | 1.50 (0.85-2.63) | 0.163 |
|  |  | Mid-high | 0.96 (0.69-1.33) | 0.793 | 1.34 (0.94-1.89) | 0.102 | 0.93 (0.69-1.25) | 0.628 | 0.93 (0.68-1.28) | 0.669 | 1.11 (0.79-1.54) | 0.551 | 1.30 (0.98-1.72) | 0.067 | 1.32 (0.98-1.79) | 0.07 | **1.19 (1.06-1.34)** | **0.005** | 1.23 (0.93-1.62) | 0.154 | 0.93 (0.53-1.63) | 0.808 | 0.68 (0.37-1.27) | 0.227 | **1.99 (1.15-3.45)** | **0.014** |
|  |  | Middle | 0.85 (0.66-1.08) | 0.178 | 1.24 (0.96-1.59) | 0.099 | 1.20 (0.96-1.51) | 0.115 | 1.22 (0.93-1.61) | 0.148 | 0.88 (0.67-1.16) | 0.358 | 1.22 (0.96-1.55) | 0.107 | 1.32 (0.98-1.79) | 0.07 | 0.92 (0.67-1.26) | 0.590 | 1.23 (0.93-1.62) | 0.154 | 0.82 (0.51-1.32) | 0.406 | 0.86 (0.52-1.42) | 0.556 | **1.57 (1.03-2.39)** | **0.038** |
|  |  | Mid-low | 1.05 (0.67-1.64) | 0.847 | 1.36 (0.86-2.17) | 0.188 | 0.81 (0.53-1.23) | 0.317 | 1.36 (0.89-2.07) | 0.159 | 1.24 (0.81-1.91) | 0.324 | 1.02 (0.67-1.53) | 0.938 | 0.82 (0.51-1.30) | 0.386 | 1.47 (0.90-2.39) | 0.122 | 0.95 (0.62-1.45) | 0.820 | 0.91 (0.50-1.67) | 0.761 | 1.05 (0.54-2.07) | 0.881 | 0.84 (0.45-1.56) | 0.578 |
|  |  | low | 1.09 (0.57-2.08) | 0.800 | 0.69 (0.34-1.41) | 0.312 | 1.53 (0.82-2.86) | 0.187 | 0.92 (0.49-1.74) | 0.800 | 1.38 (0.66-2.85) | 0.391 | 1.00 (0.52-1.94) | 0.994 | 1.00 (0.55-1.85) | 0.990 | 1.12 (0.56-2.24) | 0.760 | 0.99 (0.53-1.84) | 0.976 | 0.60 (0.28-1.30) | 0.196 | 1.67 (0.73-3.85) | 0.229 | 1.17 (0.62-2.21) | 0.626 |
| Parental educational attainment | High school diploma or less | High | **0.75 (0.66-0.85)** | **<0.001** | **1.46 (1.29-1.66)** | **<0.001** | **1.14 (1.02-1.28)** | **0.026** | **0.84 (0.74-0.95)** | **0.007** | **1.14 (1.01-1.29)** | **0.032** | 1.12 (0.99-1.26) | 0.078 | **0.70 (0.59-0.83)** | **<0.001** | **1.25 (1.05-1.49)** | **0.014** | **1.28 (1.08-1.51)** | **0.004** | **0.60 (0.42-0.86)** | **0.006** | 1.40 (0.94-2.08) | 0.099 | 1.18 (0.83-1.69) | 0.365 |
|  |  | Mid-high | **0.77 (0.71-0.83)** | **<0.001** | **1.25 (1.16-1.34)** | **<0.001** | **1.13 (1.06-1.21)** | **<0.001** | **0.81 (0.75-0.88)** | **<0.001** | 1.08 (1.00-1.17) | 0.060 | **1.13 (1.05-1.21)** | **0.002** | 0.89 (0.61-1.32) | 0.572 | 1.19 (0.80-1.77) | 0.392 | 1.34 (0.96-1.88) | 0.084 | **0.78 (0.61-0.98)** | **0.032** | 1.01 (0.80-1.28) | 0.926 | 1.09 (0.86-1.38) | 0.483 |
|  |  | Middle | **0.76 (0.71-0.81)** | **<0.001** | **1.25 (1.17-1.33)** | **<0.001** | **1.08 (1.01-1.15)** | **0.025** | **0.83 (0.77-0.89)** | **<0.001** | 1.07 (0.99-1.14) | 0.084 | **1.09 (1.02-1.16)** | **0.011** | **0.75 (0.68-0.83)** | **<0.001** | **1.27 (1.15-1.39)** | **<0.001** | 1.09 (1.00-1.18) | 0.051 | **0.75 (0.61-0.92)** | **0.005** | 1.01 (0.82-1.25) | 0.923 | **1.29 (1.06-1.58)** | **0.012** |
|  |  | Mid-low | **0.83 (0.72-0.96)** | **0.013** | 1.13 (0.99-1.30) | 0.071 | 1.11 (0.96-1.27) | 0.150 | 0.87 (0.75-1.01) | 0.064 | 0.99 (0.86-1.14) | 0.856 | 1.22 (1.06-1.40) | 0.006 | 0.86 (0.72-1.03) | 0.092 | 1.18 (0.99-1.39) | 0.061 | 1.04 (0.88-1.23) | 0.665 | 0.80 (0.58-1.13) | 0.203 | 0.91 (0.64-1.30) | 0.615 | 0.91 (0.62-1.33) | 0.619 |
|  |  | low | 0.89 (0.64-1.24) | 0.496 | 1.37 (0.96-1.96) | 0.086 | 0.97 (0.66-1.41) | 0.864 | 0.81 (0.58-1.14) | 0.225 | 1.39 (0.97-1.97) | 0.069 | 0.90 (0.62-1.30) | 0.564 | **0.63 (0.44-0.91)** | **0.014** | **1.70 (1.16-2.49)** | **0.007** | 0.91 (0.61-1.35) | 0.622 | **0.35 (0.19-0.65)** | **0.001** | **2.28 (1.20-4.33)** | **0.012** | 1.03 (0.57-1.87) | 0.915 |
|  | Bachelor's degree or higher | High | **0.75 (0.66-0.85)** | **<0.001** | **1.47 (1.29-1.68)** | **<0.001** | **1.19 (1.05-1.34)** | **0.007** | **0.83 (0.72-0.96)** | **0.011** | **1.33 (1.14-1.56)** | **<0.001** | 1.14 (0.99-1.32) | 0.068 | **0.79 (0.65-0.97)** | **0.025** | **1.33 (1.07-1.64)** | **0.009** | **1.41 (1.15-1.72)** | **0.001** | **0.62 (0.43-0.88)** | **0.008** | 1.02 (0.68-1.53) | 0.936 | **2.05 (1.43-2.94)** | **<0.001** |
|  |  | Mid-high | **0.80 (0.74-0.87)** | **<0.001** | **1.24 (1.13-1.35)** | **<0.001** | 1.04 (0.95-1.13) | 0.413 | **0.88 (0.81-0.97)** | **0.007** | **1.12 (1.02-1.23)** | **0.019** | 1.06 (0.96-1.16) | 0.240 | **0.80 (0.71-0.89)** | **<0.001** | **1.19 (1.07-1.32)** | **0.001** | 1.11 (1.00-1.22) | 0.046 | **0.74 (0.55-0.99)** | **0.041** | 1.01 (0.73-1.39) | 0.958 | **1.39 (1.05-1.85)** | **0.022** |
|  |  | Middle | **0.79 (0.74-0.84)** | **<0.001** | **1.16 (1.09-1.24)** | **<0.001** | **1.16 (1.08-1.23)** | **<0.001** | **0.90 (0.84-0.96)** | **0.001** | **1.08 (1.01-1.16)** | **0.019** | **1.11 (1.04-1.19)** | **0.002** | **0.86 (0.79-0.94)** | **0.001** | **1.15 (1.05-1.25)** | **0.004** | **1.17 (1.07-1.28)** | **0.001** | **0.68 (0.57-0.81)** | **<0.001** | 1.18 (0.97-1.43) | 0.091 | 1.17 (0.97-1.40) | 0.101 |
|  |  | Mid-low | **0.77 (0.69-0.86)** | **<0.001** | **1.22 (1.09-1.36)** | **<0.001** | 1.07 (0.95-1.20) | 0.252 | **0.82 (0.73-0.91)** | **<0.001** | **1.18 (1.05-1.33)** | **0.004** | 1.04 (0.93-1.18) | 0.487 | **0.72 (0.63-0.83)** | **<0.001** | **1.27 (1.10-1.46)** | **0.001** | **1.22 (1.05-1.41)** | **0.010** | **0.53 (0.42-0.68)** | **<0.001** | **1.40 (1.05-1.85)** | **0.020** | 0.97 (0.72-1.30) | 0.826 |
|  |  | low | **0.78 (0.63-0.97)** | **0.025** | 1.02 (0.82-1.27) | 0.852 | **1.55 (1.24-1.95)** | **<0.001** | 0.83 (0.68-1.01) | 0.060 | 1.01 (0.82-1.23) | 0.951 | 1.15 (0.92-1.44) | 0.232 | **0.78 (0.61-0.99)** | **0.042** | 1.13 (0.89-1.43) | 0.331 | 1.26 (0.99-1.62) | 0.063 | **0.66 (0.46-0.96)** | **0.028** | 1.12 (0.76-1.65) | 0.555 | 1.41 (0.99-2.01) | 0.055 |
| Academic achievement | Low | High | **0.75 (0.67-0.84)** | **<0.001** | **1.52 (1.34-1.71)** | **<0.001** | 1.11 (0.99-1.24) | 0.066 | **0.82 (0.73-0.93)** | **0.002** | **1.24 (1.10-1.39)** | **<0.001** | 1.05 (0.94-1.18) | 0.386 | **0.80 (0.67-0.95)** | **0.009** | **1.31 (1.11-1.56)** | **0.002** | **1.19 (1.01-1.40)** | **0.041** | **0.57 (0.41-0.79)** | **<0.001** | 1.35 (0.93-1.97) | 0.119 | 1.21 (0.86-1.71) | 0.267 |
|  |  | Mid-high | **0.78 (0.72-0.84)** | **<0.001** | **1.27 (1.18-1.38)** | **<0.001** | **1.13 (1.05-1.22)** | **0.002** | **0.86 (0.79-0.94)** | **<0.001** | 1.03 (0.95-1.13) | 0.464 | **1.14 (1.05-1.24)** | **0.002** | 0.89 (0.79-1.01) | 0.077 | **1.19 (1.04-1.36)** | **0.01** | 1.12 (0.98-1.27) | 0.095 | **0.74 (0.55-0.99)** | **0.046** | 1.03 (0.75-1.41) | 0.865 | **1.40 (1.05-1.85)** | **0.020** |
|  |  | Middle | **0.76 (0.70-0.82)** | **<0.001** | **1.26 (1.17-1.37)** | **<0.001** | **1.11 (1.03-1.20)** | **0.009** | **0.86 (0.79-0.94)** | **<0.001** | 1.08 (0.99-1.19) | 0.081 | **1.12 (1.02-1.23)** | **0.016** | **0.84 (0.74-0.95)** | **0.004** | **1.22 (1.08-1.39)** | **0.002** | **1.22 (1.08-1.37)** | **0.001** | **0.68 (0.51-0.89)** | **0.005** | 1.03 (0.75-1.40) | 0.858 | **1.38 (1.01-1.88)** | **0.043** |
|  |  | Mid-low | **0.70 (0.59-0.84)** | **<0.001** | **1.32 (1.10-1.60)** | **0.004** | 1.04 (0.86-1.27) | 0.670 | 0.88 (0.74-1.04) | 0.130 | 1.03 (0.85-1.24) | 0.781 | 1.19 (0.98-1.45) | 0.079 | 0.83 (0.67-1.02) | 0.077 | 1.22 (0.97-1.54) | 0.096 | 1.03 (0.81-1.30) | 0.824 | 0.68 (0.42-1.09) | 0.105 | 1.30 (0.80-2.13) | 0.290 | 0.79 (0.47-1.32) | 0.363 |
|  |  | low | 0.89 (0.60-1.31) | 0.540 | 1.40 (0.94-2.09) | 0.098 | 1.04 (0.67-1.61) | 0.853 | 0.77 (0.52-1.14) | 0.195 | **1.79 (1.19-2.70)** | **0.006** | 1.07 (0.71-1.61) | 0.754 | 0.76 (0.48-1.21) | 0.250 | **1.93 (1.22-3.06)** | **0.005** | 0.84 (0.53-1.34) | 0.472 | 0.61 (0.28-1.32) | 0.213 | 1.43 (0.69-2.96) | 0.341 | 0.72 (0.36-1.44) | 0.355 |
|  | Middle | High | **0.73 (0.61-0.88)** | **0.001** | **1.56 (1.29-1.89)** | **<0.001** | **1.23 (1.02-1.49)** | **0.033** | 0.89 (0.72-1.10) | 0.292 | **1.28 (1.03-1.60)** | **0.027** | **1.27 (1.02-1.57)** | **0.030** | **0.64 (0.47-0.88)** | **0.006** | **1.42 (1.01-1.98)** | **0.042** | 1.37 (1.00-1.86) | 0.047 | 0.58 (0.31-1.10) | 0.097 | 1.05 (0.51-2.19) | 0.889 | **2.28 (1.21-4.28)** | **0.011** |
|  |  | Mid-high | **0.79 (0.72-0.87)** | **<0.001** | **1.19 (1.08-1.31)** | **<0.001** | 1.06 (0.96-1.16) | 0.254 | **0.89 (0.80-0.99)** | **0.033** | 1.11 (0.99-1.23) | 0.074 | 1.07 (0.96-1.20) | 0.218 | **0.87 (0.77-0.98)** | **0.022** | **1.15 (1.02-1.30)** | **0.027** | **1.18 (1.05-1.33)** | **0.006** | **0.67 (0.48-0.95)** | **0.022** | 1.16 (0.81-1.66) | 0.406 | 1.07 (0.77-1.47) | 0.698 |
|  |  | Middle | **0.76 (0.71-0.83)** | **<0.001** | **1.24 (1.14-1.34)** | **<0.001** | **1.11 (1.03-1.19)** | **0.008** | **0.84 (0.77-0.91)** | **<0.001** | 1.08 (0.99-1.16) | 0.072 | **1.09 (1.01-1.18)** | **0.036** | **0.77 (0.69-0.86)** | **<0.001** | **1.20 (1.07-1.34)** | **0.001** | 1.08 (0.97-1.19) | 0.174 | **0.66 (0.52-0.85)** | **0.001** | 1.20 (0.93-1.56) | 0.160 | 1.06 (0.83-1.36) | 0.635 |
|  |  | Mid-low | **0.80 (0.68-0.93)** | **0.004** | 1.17 (0.99-1.38) | 0.061 | 1.03 (0.86-1.23) | 0.747 | 0.89 (0.75-1.06) | 0.180 | 1.09 (0.91-1.31) | 0.354 | 0.98 (0.81-1.18) | 0.831 | **0.75 (0.61-0.92)** | **0.006** | 1.23 (0.99-1.51) | 0.058 | 1.11 (0.89-1.40) | 0.355 | **0.63 (0.42-0.94)** | **0.022** | 1.23 (0.79-1.91) | 0.358 | 1.03 (0.66-1.63) | 0.890 |
|  |  | low | 0.90 (0.60-1.35) | 0.608 | 1.01 (0.65-1.59) | 0.952 | **1.78 (1.08-2.91)** | **0.023** | 0.90 (0.59-1.37) | 0.621 | 1.14 (0.73-1.77) | 0.562 | 0.92 (0.57-1.49) | 0.738 | 0.73 (0.43-1.22) | 0.223 | 1.54 (0.90-2.64) | 0.114 | 1.52 (0.91-2.53) | 0.110 | 0.43 (0.18-1.02) | 0.056 | 1.65 (0.64-4.27) | 0.304 | 1.68 (0.70-4.03) | 0.246 |
|  | High | High | **0.77 (0.64-0.93)** | **0.006** | **1.27 (1.06-1.53)** | **0.012** | **1.26 (1.05-1.51)** | **0.014** | 0.86 (0.70-1.05) | 0.140 | 1.15 (0.94-1.41) | 0.185 | **1.26 (1.03-1.53)** | **0.026** | **0.73 (0.56-0.95)** | **0.021** | 1.10 (0.82-1.46) | 0.531 | **1.79 (1.37-2.35)** | **<0.001** | 0.62 (0.39-1.01) | 0.054 | 1.01 (0.61-1.68) | 0.955 | **1.99 (1.27-3.13)** | **0.003** |
|  |  | Mid-high | **0.78 (0.70-0.88)** | **<0.001** | **1.28 (1.14-1.45)** | **<0.001** | 1.07 (0.96-1.20) | 0.242 | **0.76 (0.68-0.85)** | **<0.001** | **1.24 (1.10-1.40)** | **<0.001** | 1.07 (0.95-1.19) | 0.279 | **0.77 (0.66-0.90)** | **0.001** | **1.39 (1.19-1.63)** | **<0.001** | 0.99 (0.86-1.15) | 0.893 | 0.86 (0.63-1.18) | 0.339 | 0.92 (0.66-1.29) | 0.625 | 1.06 (0.76-1.47) | 0.727 |
|  |  | Middle | **0.79 (0.73-0.85)** | **<0.001** | **1.14 (1.06-1.22)** | **<0.001** | **1.13 (1.05-1.22)** | **<0.001** | **0.90 (0.83-0.97)** | **0.007** | 1.07 (0.99-1.16) | 0.076 | **1.10 (1.02-1.19)** | **0.010** | **0.82 (0.75-0.91)** | **<0.001** | **1.20 (1.09-1.33)** | **<0.001** | **1.13 (1.02-1.24)** | **0.014** | **0.73 (0.60-0.88)** | **0.001** | 1.07 (0.88-1.31) | 0.484 | **1.26 (1.04-1.54)** | **0.020** |
|  |  | Mid-low | **0.84 (0.75-0.94)** | **0.002** | **1.13 (1.01-1.27)** | **0.041** | **1.14 (1.01-1.30)** | **0.037** | **0.80 (0.71-0.91)** | **<0.001** | 1.13 (1.00-1.28) | 0.044 | **1.16 (1.02-1.31)** | **0.021** | **0.77 (0.66-0.89)** | **0.001** | **1.23 (1.06-1.43)** | **0.008** | **1.22 (1.05-1.41)** | **0.009** | **0.61 (0.47-0.80)** | **<0.001** | 1.10 (0.81-1.50) | 0.531 | 0.98 (0.71-1.34) | 0.888 |
|  |  | low | **0.76 (0.60-0.96)** | **0.021** | 1.04 (0.83-1.30) | 0.763 | **1.40 (1.10-1.77)** | **0.006** | 0.81 (0.65-1.00) | 0.051 | 0.94 (0.74-1.18) | 0.589 | 1.13 (0.89-1.45) | 0.314 | **0.73 (0.57-0.93)** | **0.012** | 1.05 (0.82-1.34) | 0.71 | 1.21 (0.93-1.57) | 0.15 | **0.59 (0.40-0.86)** | **0.007** | 1.25 (0.83-1.89) | 0.287 | 1.42 (0.97-2.06) | 0.071 |

Abbreviations: CI, Confidence Interval; KYRBS, the Korea Youth Risk Behavior Survey; wOR, weighted odds ratio.

Numbers in bold indicate a significant difference (p< 0.05).

**Table S5.** Two-year comparative analysis of adolescents’ mental health indicators with various influencing factors, before and during the COVID–19 period: using wOR (95% CI) based on KYRBS, 2006–2022

| Income level | Variables | | Overall (2006 to 2022) | 2019 | 2020 vs 2019 | 2020 | 2021 vs 2020 | 2021 | 2022 vs 2021 | 2022 |
| --- | --- | --- | --- | --- | --- | --- | --- | --- | --- | --- |
| **High income** | **Perceived stress level** | | | | | | | | | |
|  | Sex | Male | 1.00 (Reference) | 1.00 (Reference) | 1.00 (Reference) | 1.00 (Reference) | 1.00 (Reference) | 1.00 (Reference) | 1.00 (Reference) | 1.00 (Reference) |
|  |  | Female | **1.51 (1.46-1.56)** | **1.94 (1.79-2.11)** | **0.77 (0.66-0.91)** | **1.50 (1.31-1.72)** | 1.11 (0.92-1.33) | **1.66 (1.46-1.87)** | 0.85 (0.72-1.00) | **1.41 (1.26-1.58)** |
|  | Grade level | Middle school  (7-9^th^) | 1.00 (Reference) | 1.00 (Reference) | 1.00 (Reference) | 1.00 (Reference) | 1.00 (Reference) | 1.00 (Reference) | 1.00 (Reference) | 1.00 (Reference) |
|  |  | High school  (10-12^th^) | **1.38 (1.34-1.43)** | **1.40 (1.28-1.52)** | 1.12 (0.95-1.32) | **1.57 (1.36-1.81)** | **0.78 (0.65-0.95)** | **1.23 (1.09-1.40)** | 1.07 (0.89-1.27) | **1.31 (1.16-1.48)** |
|  | Residential area | Rural | 1.00 (Reference) | 1.00 (Reference) | 1.00 (Reference) | 1.00 (Reference) | 1.00 (Reference) | 1.00 (Reference) | 1.00 (Reference) | 1.00 (Reference) |
|  |  | Urban | 1.04 (1.00-1.08) | 1.00 (0.92-1.09) | 1.12 (0.95-1.32) | 1.12 (0.97-1.29) | 0.86 (0.71-1.04) | 0.96 (0.85-1.09) | 1.01 (0.85-1.2) | 0.97 (0.86-1.08) |
|  | Recent alcohol consumption | No | 1.00 (Reference) | 1.00 (Reference) | 1.00 (Reference) | 1.00 (Reference) | 1.00 (Reference) | 1.00 (Reference) | 1.00 (Reference) | 1.00 (Reference) |
|  |  | Yes | **1.59 (1.53-1.66)** | **1.72 (1.56-1.91)** | 0.97 (0.79-1.20) | **1.67 (1.39-2.01)** | 0.9 (0.69-1.18) | **1.51 (1.24-1.83)** | 0.94 (0.72-1.22) | **1.42 (1.19-1.70)** |
|  | Smoking status | No | 1.00 (Reference) | 1.00 (Reference) | 1.00 (Reference) | 1.00 (Reference) | 1.00 (Reference) | 1.00 (Reference) | 1.00 (Reference) | 1.00 (Reference) |
|  |  | Yes | **1.66 (1.58-1.75)** | **1.68 (1.45-1.94)** | **1.70 (1.17-2.49)** | **2.86 (2.02-4.07)** | **0.50 (0.32-0.80)** | **1.44 (1.06-1.96)** | 1.15 (0.76-1.74) | **1.65 (1.25-2.18)** |
|  | Parental educational attainment | High school diploma or less | 1.00 (Reference) | 1.00 (Reference) | 1.00 (Reference) | 1.00 (Reference) | 1.00 (Reference) | 1.00 (Reference) | 1.00 (Reference) | 1.00 (Reference) |
|  |  | Bachelor's degree or higher | 0.98 (0.95-1.01) | 1.04 (0.96-1.12) | 1.00 (0.86-1.16) | 1.04 (0.91-1.17) | 0.99 (0.83-1.18) | 1.03 (0.91-1.16) | 0.96 (0.82-1.13) | 0.99 (0.89-1.10) |
|  | Academic achievement | Low | **1.08 (1.03-1.12)** | 1.08 (0.97-1.21) | 1.03 (0.86-1.24) | 1.11 (0.96-1.29) | 0.97 (0.78-1.21) | 1.08 (0.92-1.27) | 0.91 (0.73-1.13) | 0.98 (0.84-1.13) |
|  |  | Middle | 1.00 (Reference) | 1.00 (Reference) | 1.00 (Reference) | 1.00 (Reference) | 1.00 (Reference) | 1.00 (Reference) | 1.00 (Reference) | 1.00 (Reference) |
|  |  | High | **1.35 (1.29-1.42)** | **1.38 (1.21-1.57)** | 1.08 (0.86-1.36) | **1.49 (1.23-1.80)** | 0.81 (0.62-1.06) | **1.21 (1.01-1.46)** | 1.03 (0.79-1.33) | **1.24 (1.03-1.49)** |
|  | **Sadness** | | | | | | | | | |
|  | Sex | Male | 1.00 (Reference) | 1.00 (Reference) | 1.00 (Reference) | 1.00 (Reference) | 1.00 (Reference) | 1.00 (Reference) | 1.00 (Reference) | 1.00 (Reference) |
|  |  | Female | **1.41 (1.36-1.46)** | **1.82 (1.66-1.98)** | 0.80 (0.68-0.95) | **1.46 (1.27-1.68)** | 1.01 (0.84-1.23) | **1.48 (1.31-1.69)** | 1.04 (0.87-1.25) | **1.54 (1.35-1.75)** |
|  | Grade level | Middle school  (7-9^th^) | 1.00 (Reference) | 1.00 (Reference) | 1.00 (Reference) | 1.00 (Reference) | 1.00 (Reference) | 1.00 (Reference) | 1.00 (Reference) | 1.00 (Reference) |
|  |  | High school  (10-12^th^) | **1.32 (1.27-1.37)** | **1.27 (1.16-1.39)** | **1.23 (1.04-1.45)** | **1.56 (1.36-1.79)** | 0.67 (0.55-0.82) | 1.05 (0.92-1.21) | 1.19 (0.98-1.44) | **1.25 (1.10-1.43)** |
|  | Residential area | Rural | 1.00 (Reference) | 1.00 (Reference) | 1.00 (Reference) | 1.00 (Reference) | 1.00 (Reference) | 1.00 (Reference) | 1.00 (Reference) | 1.00 (Reference) |
|  |  | Urban | 1.02 (0.98-1.06) | 0.99 (0.91-1.08) | 1.16 (0.99-1.37) | 1.15 (1.00-1.32) | 0.94 (0.78-1.14) | 1.08 (0.95-1.23) | 0.96 (0.80-1.16) | 1.04 (0.91-1.18) |
|  | Recent alcohol consumption | No | 1.00 (Reference) | 1.00 (Reference) | 1.00 (Reference) | 1.00 (Reference) | 1.00 (Reference) | 1.00 (Reference) | 1.00 (Reference) | 1.00 (Reference) |
|  |  | Yes | **2.35 (2.25-2.45)** | **2.54 (2.28-2.84)** | 0.82 (0.65-1.03) | **2.08 (1.71-2.53)** | 1.01 (0.77-1.33) | **2.10 (1.73-2.55)** | 1.05 (0.81-1.35) | **2.20 (1.86-2.60)** |
|  | Smoking status | No | 1.00 (Reference) | 1.00 (Reference) | 1.00 (Reference) | 1.00 (Reference) | 1.00 (Reference) | 1.00 (Reference) | 1.00 (Reference) | 1.00 (Reference) |
|  |  | Yes | **2.41 (2.29-2.54)** | **2.31 (1.98-2.71)** | 1.31 (0.88-1.94) | **3.02 (2.10-4.33)** | 0.82 (0.51-1.31) | **2.46 (1.82-3.33)** | 1.13 (0.77-1.68) | **2.79 (2.17-3.58)** |
|  | Parental educational attainment | High school diploma or less | 1.00 (Reference) | 1.00 (Reference) | 1.00 (Reference) | 1.00 (Reference) | 1.00 (Reference) | 1.00 (Reference) | 1.00 (Reference) | 1.00 (Reference) |
|  |  | Bachelor's degree or higher | **1.05 (1.01-1.08)** | **1.12 (1.03-1.23)** | 1.03 (0.87-1.22) | 1.15 (0.99-1.33) | 0.85 (0.70-1.04) | 0.98 (0.86-1.11) | 0.98 (0.82-1.18) | 0.96 (0.84-1.10) |
|  | Academic achievement | Low | 1.00 (0.96-1.05) | 1.03 (0.91-1.16) | 0.92 (0.74-1.14) | 0.95 (0.80-1.14) | 0.97 (0.76-1.24) | 0.92 (0.77-1.09) | 0.83 (0.65-1.05) | 0.76 (0.64-0.90) |
|  |  | Middle | 1.00 (Reference) | 1.00 (Reference) | 1.00 (Reference) | 1.00 (Reference) | 1.00 (Reference) | 1.00 (Reference) | 1.00 (Reference) | 1.00 (Reference) |
|  |  | High | **1.38 (1.31-1.45)** | **1.34 (1.16-1.55)** | 1.08 (0.84-1.40) | **1.45 (1.17-1.80)** | 0.90 (0.66-1.21) | **1.30 (1.06-1.61)** | 0.99 (0.74-1.33) | **1.29 (1.05-1.58)** |
|  | **Suicidal ideation** | | | | | | | | | |
|  | Sex | Male | 1.00 (Reference) | 1.00 (Reference) | 1.00 (Reference) | 1.00 (Reference) | 1.00 (Reference) | 1.00 (Reference) | 1.00 (Reference) | 1.00 (Reference) |
|  |  | Female | 0.71 (0.68-0.74) | **1.82 (1.62-2.05)** | 0.76 (0.60-0.97) | **1.38 (1.12-1.71)** | 1.24 (0.94-1.64) | **1.71 (1.42-2.06)** | 1.05 (0.81-1.35) | **1.79 (1.50-2.13)** |
|  | Grade level | Middle school  (7-9^th^) | 1.00 (Reference) | 1.00 (Reference) | 1.00 (Reference) | 1.00 (Reference) | 1.00 (Reference) | 1.00 (Reference) | 1.00 (Reference) | 1.00 (Reference) |
|  |  | High school  (10-12^th^) | **0.90 (0.86-0.95)** | **1.20 (1.05-1.36)** | 1.17 (0.91-1.49) | **1.40 (1.14-1.73)** | **0.69 (0.52-0.92)** | 0.97 (0.80-1.17) | 1.07 (0.83-1.39) | 1.04 (0.87-1.24) |
|  | Residential area | Rural | 1.00 (Reference) | 1.00 (Reference) | 1.00 (Reference) | 1.00 (Reference) | 1.00 (Reference) | 1.00 (Reference) | 1.00 (Reference) | 1.00 (Reference) |
|  |  | Urban | **0.94 (0.90-0.99)** | 1.04 (0.91-1.18) | 0.95 (0.75-1.22) | 0.99 (0.80-1.21) | 1.14 (0.86-1.51) | 1.13 (0.93-1.36) | 0.93 (0.72-1.20) | 1.05 (0.88-1.25) |
|  | Recent alcohol consumption | No | 1.00 (Reference) | 1.00 (Reference) | 1.00 (Reference) | 1.00 (Reference) | 1.00 (Reference) | 1.00 (Reference) | 1.00 (Reference) | 1.00 (Reference) |
|  |  | Yes | **0.43 (0.41-0.45)** | **2.66 (2.34-3.03)** | 0.90 (0.67-1.19) | **2.38 (1.84-3.07)** | 1.03 (0.74-1.46) | **2.46 (1.96-3.08)** | 0.94 (0.69-1.29) | **2.32 (1.88-2.87)** |
|  | Smoking status | No | 1.00 (Reference) | 1.00 (Reference) | 1.00 (Reference) | 1.00 (Reference) | 1.00 (Reference) | 1.00 (Reference) | 1.00 (Reference) | 1.00 (Reference) |
|  |  | Yes | **0.37 (0.35-0.39)** | **2.92 (2.44-3.49)** | 1.34 (0.87-2.07) | **3.91 (2.63-5.80)** | 0.82 (0.49-1.39) | **3.22 (2.30-4.51)** | 1.27 (0.83-1.95) | **4.08 (3.12-5.32)** |
|  | Parental educational attainment | High school diploma or less | 1.00 (Reference) | 1.00 (Reference) | 1.00 (Reference) | 1.00 (Reference) | 1.00 (Reference) | 1.00 (Reference) | 1.00 (Reference) | 1.00 (Reference) |
|  |  | Bachelor's degree or higher | 1.00 (0.96-1.05) | **1.15 (1.02-1.30)** | 0.97 (0.77-1.21) | 1.11 (0.92-1.34) | 0.94 (0.73-1.21) | 1.04 (0.86-1.27) | 0.91 (0.71-1.17) | 0.95 (0.80-1.13) |
|  | Academic achievement | Low | 0.86 (0.81-0.92) | 1.17 (0.99-1.38) | 1.20 (0.87-1.65) | **1.40 (1.06-1.84)** | 0.92 (0.64-1.34) | **1.29 (1.01-1.66)** | 0.87 (0.61-1.23) | 1.12 (0.88-1.43) |
|  |  | Middle | 1.00 (Reference) | 1.00 (Reference) | 1.00 (Reference) | 1.00 (Reference) | 1.00 (Reference) | 1.00 (Reference) | 1.00 (Reference) | 1.00 (Reference) |
|  |  | High | 0.68 (0.63-0.73) | **1.56 (1.29-1.87)** | 1.06 (0.74-1.54) | **1.66 (1.21-2.28)** | 0.78 (0.51-1.2) | 1.29 (0.96-1.72) | 1.31 (0.87-1.97) | **1.69 (1.27-2.24)** |
|  | **Suicidal attempt** | | | | | | | | | |
|  | Sex | Male | 1.00 (Reference) | 1.00 (Reference) | 1.00 (Reference) | 1.00 (Reference) | 1.00 (Reference) | 1.00 (Reference) | 1.00 (Reference) | 1.00 (Reference) |
|  |  | Female | **1.21 (1.12-1.30)** | **1.43 (1.16-1.77)** | 0.77 (0.48-1.24) | 1.10 (0.72-1.68) | 1.70 (0.97-2.97) | **1.87 (1.30-2.68)** | 0.76 (0.47-1.24) | **1.42 (1.02-1.98)** |
|  | Grade level | Middle school  (7-9^th^) | 1.00 (Reference) | 1.00 (Reference) | 1.00 (Reference) | 1.00 (Reference) | 1.00 (Reference) | 1.00 (Reference) | 1.00 (Reference) | 1.00 (Reference) |
|  |  | High school  (10-12^th^) | 1.02 (0.94-1.10) | 0.95 (0.77-1.17) | **1.85 (1.19-2.9)** | **1.76 (1.19-2.62)** | 0.63 (0.35-1.13) | 1.10 (0.71-1.70) | 1.33 (0.77-2.28) | **1.46 (1.06-2.01)** |
|  | Residential area | Rural | 1.00 (Reference) | 1.00 (Reference) | 1.00 (Reference) | 1.00 (Reference) | 1.00 (Reference) | 1.00 (Reference) | 1.00 (Reference) | 1.00 (Reference) |
|  |  | Urban | 1.01 (0.93-1.10) | 1.15 (0.94-1.41) | **0.61 (0.39-0.95)** | 0.70 (0.47-1.03) | 1.27 (0.73-2.23) | 0.89 (0.60-1.34) | 1.24 (0.74-2.07) | 1.10 (0.80-1.52) |
|  | Recent alcohol consumption | No | 1.00 (Reference) | 1.00 (Reference) | 1.00 (Reference) | 1.00 (Reference) | 1.00 (Reference) | 1.00 (Reference) | 1.00 (Reference) | 1.00 (Reference) |
|  |  | Yes | **3.25 (3.02-3.50)** | **4.17 (3.38-5.15)** | 1.3 (0.81-2.07) | **5.41 (3.57-8.22)** | 0.97 (0.54-1.73) | **5.24 (3.51-7.83)** | 0.81 (0.48-1.36) | **4.22 (3.02-5.89)** |
|  | Smoking status | No | 1.00 (Reference) | 1.00 (Reference) | 1.00 (Reference) | 1.00 (Reference) | 1.00 (Reference) | 1.00 (Reference) | 1.00 (Reference) | 1.00 (Reference) |
|  |  | Yes | **4.44 (4.09-4.82)** | **5.88 (4.62-7.49)** | **2.01 (1.11-3.65)** | **11.82 (6.86-20.36)** | 0.61 (0.29-1.28) | **7.21 (4.34-11.97)** | 1.09 (0.57-2.07) | **7.84 (5.29-11.61)** |
|  | Parental educational attainment | High school diploma or less | 1.00 (Reference) | 1.00 (Reference) | 1.00 (Reference) | 1.00 (Reference) | 1.00 (Reference) | 1.00 (Reference) | 1.00 (Reference) | 1.00 (Reference) |
|  |  | Bachelor's degree or higher | **0.69 (0.64-0.75)** | **0.64 (0.51-0.79)** | 1.11 (0.69-1.78) | 0.71 (0.47-1.09) | 1.38 (0.78-2.46) | 0.98 (0.66-1.45) | **0.57 (0.34-0.95)** | **0.56 (0.41-0.78)** |
|  | Academic achievement | Low | **1.40 (1.25-1.57)** | 1.27 (0.95-1.71) | 1.02 (0.53-1.97) | 1.29 (0.71-2.32) | 1.28 (0.57-2.89) | 1.65 (0.94-2.88) | 0.53 (0.27-1.07) | 0.88 (0.58-1.34) |
|  |  | Middle | 1.00 (Reference) | 1.00 (Reference) | 1.00 (Reference) | 1.00 (Reference) | 1.00 (Reference) | 1.00 (Reference) | 1.00 (Reference) | 1.00 (Reference) |
|  |  | High | **1.92 (1.68-2.00)** | **1.72 (1.23-2.41)** | 1.06 (0.54-2.11) | **1.83 (1.01-3.33)** | 0.96 (0.4-2.29) | 1.76 (0.94-3.32) | 0.88 (0.4-1.89) | 1.54 (0.99-2.41) |
| **Mid-high income** | **Perceived stress level** | | | | | | | | | |
|  | Sex | Male | 1.00 (Reference) | 1.00 (Reference) | 1.00 (Reference) | 1.00 (Reference) | 1.00 (Reference) | 1.00 (Reference) | 1.00 (Reference) | 1.00 (Reference) |
|  |  | Female | **1.71 (1.68-1.75)** | **2.13 (2.02-2.25)** | **0.83 (0.75-0.92)** | **1.77 (1.63-1.92)** | 0.94 (0.84-1.05) | **1.66 (1.53-1.79)** | 0.98 (0.88-1.09) | **1.63 (1.51-1.75)** |
|  | Grade level | Middle school  (7-9^th^) | 1.00 (Reference) | 1.00 (Reference) | 1.00 (Reference) | 1.00 (Reference) | 1.00 (Reference) | 1.00 (Reference) | 1.00 (Reference) | 1.00 (Reference) |
|  |  | High school  (10-12^th^) | **1.33 (1.30-1.36)** | **1.32 (1.24-1.40)** | **1.12 (1.01-1.24)** | **1.48 (1.36-1.61)** | **0.87 (0.77-0.97)** | **1.28 (1.18-1.39)** | 0.93 (0.83-1.04) | **1.19 (1.10-1.28)** |
|  | Residential area | Rural | 1.00 (Reference) | 1.00 (Reference) | 1.00 (Reference) | 1.00 (Reference) | 1.00 (Reference) | 1.00 (Reference) | 1.00 (Reference) | 1.00 (Reference) |
|  |  | Urban | **1.06 (1.03-1.08)** | 1.01 (0.95-1.08) | 1.04 (0.93-1.16) | 1.05 (0.96-1.14) | 0.93 (0.83-1.05) | 0.98 (0.90-1.06) | 0.99 (0.89-1.11) | 0.97 (0.90-1.05) |
|  | Recent alcohol consumption | No | 1.00 (Reference) | 1.00 (Reference) | 1.00 (Reference) | 1.00 (Reference) | 1.00 (Reference) | 1.00 (Reference) | 1.00 (Reference) | 1.00 (Reference) |
|  |  | Yes | **1.50 (1.46-1.53)** | **1.48 (1.38-1.59)** | 1.07 (0.93-1.23) | **1.58 (1.41-1.79)** | 0.85 (0.73-1.01) | **1.35 (1.21-1.51)** | 0.95 (0.81-1.11) | **1.28 (1.15-1.43)** |
|  | Smoking status | No | 1.00 (Reference) | 1.00 (Reference) | 1.00 (Reference) | 1.00 (Reference) | 1.00 (Reference) | 1.00 (Reference) | 1.00 (Reference) | 1.00 (Reference) |
|  |  | Yes | **1.45 (1.40-1.50)** | **1.29 (1.14-1.45)** | 1.19 (0.9-1.59) | **1.54 (1.19-2.01)** | 1.08 (0.77-1.53) | **1.67 (1.33-2.09)** | 0.84 (0.63-1.14) | **1.41 (1.16-1.72)** |
|  | Parental educational attainment | High school diploma or less | 1.00 (Reference) | 1.00 (Reference) | 1.00 (Reference) | 1.00 (Reference) | 1.00 (Reference) | 1.00 (Reference) | 1.00 (Reference) | 1.00 (Reference) |
|  |  | Bachelor's degree or higher | 1.04 (1.02-1.06) | 1.02 (0.97-1.08) | 0.98 (0.89-1.08) | 1.00 (0.93-1.08) | 1.01 (0.91-1.13) | 1.01 (0.94-1.10) | 1.09 (0.98-1.21) | **1.10 (1.03-1.19)** |
|  | Academic achievement | Low | **0.97 (0.94-0.99)** | 0.94 (0.89-1.00) | 0.96 (0.87-1.06) | **0.90 (0.83-0.98)** | 1.07 (0.95-1.2) | 0.96 (0.89-1.04) | 1.07 (0.96-1.2) | 1.03 (0.95-1.11) |
|  |  | Middle | 1.00 (Reference) | 1.00 (Reference) | 1.00 (Reference) | 1.00 (Reference) | 1.00 (Reference) | 1.00 (Reference) | 1.00 (Reference) | 1.00 (Reference) |
|  |  | High | **1.31 (1.28-1.34)** | **1.24 (1.16-1.33)** | 0.95 (0.84-1.08) | **1.18 (1.06-1.31)** | 1.08 (0.93-1.24) | **1.27 (1.15-1.40)** | 1.01 (0.88-1.16) | **1.28 (1.16-1.42)** |
|  | **sadness** | | | | | | | | | |
|  | Sex | Male | 1.00 (Reference) | 1.00 (Reference) | 1.00 (Reference) | 1.00 (Reference) | 1.00 (Reference) | 1.00 (Reference) | 1.00 (Reference) | 1.00 (Reference) |
|  |  | Female | **1.58 (1.55-1.61)** | **1.85 (1.74-1.96)** | 1.01 (0.91-1.12) | **1.87 (1.71-2.03)** | **0.79 (0.7-0.89)** | **1.47 (1.35-1.59)** | 1.09 (0.97-1.22) | **1.60 (1.48-1.74)** |
|  | Grade level | Middle school  (7-9^th^) | 1.00 (Reference) | 1.00 (Reference) | 1.00 (Reference) | 1.00 (Reference) | 1.00 (Reference) | 1.00 (Reference) | 1.00 (Reference) | 1.00 (Reference) |
|  |  | High school  (10-12^th^) | **1.27 (1.24-1.30)** | **1.23 (1.16-1.31)** | 1.08 (0.97-1.21) | **1.33 (1.22-1.46)** | **0.82 (0.72-0.93)** | **1.09 (1.00-1.19)** | 1.01 (0.9-1.14) | **1.10 (1.02-1.20)** |
|  | Residential area | Rural | 1.00 (Reference) | 1.00 (Reference) | 1.00 (Reference) | 1.00 (Reference) | 1.00 (Reference) | 1.00 (Reference) | 1.00 (Reference) | 1.00 (Reference) |
|  |  | Urban | **1.05 (1.03-1.08)** | **1.04 (0.98-1.10)** | 1.02 (0.92-1.13) | 1.06 (0.97-1.16) | 1.03 (0.91-1.16) | 1.09 (1.00-1.18) | 0.94 (0.83-1.05) | 1.02 (0.94-1.11) |
|  | Recent alcohol consumption | No | 1.00 (Reference) | 1.00 (Reference) | 1.00 (Reference) | 1.00 (Reference) | 1.00 (Reference) | 1.00 (Reference) | 1.00 (Reference) | 1.00 (Reference) |
|  |  | Yes | **2.02 (1.97-2.07)** | **1.96 (1.82-2.10)** | 1.07 (0.92-1.23) | **2.09 (1.85-2.37)** | 0.95 (0.80-1.13) | **1.99 (1.77-2.23)** | 0.94 (0.80-1.10) | **1.87 (1.68-2.07)** |
|  | Smoking status | No | 1.00 (Reference) | 1.00 (Reference) | 1.00 (Reference) | 1.00 (Reference) | 1.00 (Reference) | 1.00 (Reference) | 1.00 (Reference) | 1.00 (Reference) |
|  |  | Yes | **2.04 (1.97-2.11)** | **1.86 (1.65-2.09)** | 1.22 (0.92-1.6) | **2.26 (1.76-2.90)** | 1.02 (0.73-1.42) | **2.30 (1.84-2.88)** | 1.19 (0.89-1.59) | **2.74 (2.29-3.28)** |
|  | Parental educational attainment | High school diploma or less | 1.00 (Reference) | 1.00 (Reference) | 1.00 (Reference) | 1.00 (Reference) | 1.00 (Reference) | 1.00 (Reference) | 1.00 (Reference) | 1.00 (Reference) |
|  |  | Bachelor's degree or higher | **1.06 (1.04-1.08)** | **1.09 (1.03-1.15)** | 0.95 (0.86-1.04) | 1.03 (0.95-1.12) | 0.96 (0.85-1.08) | 0.99 (0.91-1.08) | 1.07 (0.95-1.20) | 1.06 (0.98-1.15) |
|  | Academic achievement | Low | **0.85 (0.83-0.87)** | **0.86 (0.81-0.92)** | **0.99 (0.88-1.11)** | **0.85 (0.77-0.93)** | 0.93 (0.81-1.06) | **0.79 (0.72-0.87)** | 1.06 (0.93-1.21) | **0.84 (0.77-0.92)** |
|  |  | Middle | 1.00 (Reference) | 1.00 (Reference) | 1.00 (Reference) | 1.00 (Reference) | 1.00 (Reference) | 1.00 (Reference) | 1.00 (Reference) | 1.00 (Reference) |
|  |  | High | **1.33 (1.30-1.37)** | **1.39 (1.29-1.50)** | 0.89 (0.77-1.02) | **1.23 (1.10-1.39)** | 1.13 (0.96-1.34) | **1.39 (1.23-1.56)** | 0.99 (0.85-1.16) | **1.38 (1.24-1.53)** |
|  | **Suicidal ideation** | | | | | | | | | |
|  | Sex | Male | 1.00 (Reference) | 1.00 (Reference) | 1.00 (Reference) | 1.00 (Reference) | 1.00 (Reference) | 1.00 (Reference) | 1.00 (Reference) | 1.00 (Reference) |
|  |  | Female | **1.41 (1.34-1.47)** | **2.05 (1.90-2.23)** | 1.00 (0.87-1.16) | **2.05 (1.82-2.32)** | 1.06 (0.91-1.24) | **1.67 (1.49-1.87)** | 1.06 (0.91-1.24) | **1.77 (1.58-1.97)** |
|  | Grade level | Middle school  (7-9^th^) | 1.00 (Reference) | 1.00 (Reference) | 1.00 (Reference) | 1.00 (Reference) | 1.00 (Reference) | 1.00 (Reference) | 1.00 (Reference) | 1.00 (Reference) |
|  |  | High school  (10-12^th^) | **1.11 (1.06-1.16)** | **0.91 (0.84-0.99)** | **1.32 (1.13-1.54)** | **1.20 (1.06-1.37)** | **0.78 (0.65-0.92)** | 0.93 (0.83-1.04) | 0.88 (0.75-1.04) | **0.82 (0.73-0.92)** |
|  | Residential area | Rural | 1.00 (Reference) | 1.00 (Reference) | 1.00 (Reference) | 1.00 (Reference) | 1.00 (Reference) | 1.00 (Reference) | 1.00 (Reference) | 1.00 (Reference) |
|  |  | Urban | **1.06 (1.01-1.11)** | 1.03 (0.96-1.12) | 1.01 (0.87-1.18) | 1.04 (0.91-1.18) | 1.03 (0.86-1.23) | 1.07 (0.95-1.20) | 0.96 (0.82-1.14) | 1.03 (0.92-1.16) |
|  | Recent alcohol consumption | No | 1.00 (Reference) | 1.00 (Reference) | 1.00 (Reference) | 1.00 (Reference) | 1.00 (Reference) | 1.00 (Reference) | 1.00 (Reference) | 1.00 (Reference) |
|  |  | Yes | **2.33 (2.22-2.45)** | **1.88 (1.71-2.06)** | **1.29 (1.08-1.55)** | **2.43 (2.08-2.83)** | **0.76 (0.61-0.95)** | **1.84 (1.56-2.16)** | 1.08 (0.87-1.33) | **1.98 (1.73-2.26)** |
|  | Smoking status | No | 1.00 (Reference) | 1.00 (Reference) | 1.00 (Reference) | 1.00 (Reference) | 1.00 (Reference) | 1.00 (Reference) | 1.00 (Reference) | 1.00 (Reference) |
|  |  | Yes | **2.74 (2.58-2.90)** | **1.87 (1.61-2.17)** | 1.17 (0.83-1.64) | **2.18 (1.60-2.96)** | 1.01 (0.67-1.5) | **2.19 (1.69-2.84)** | 1.23 (0.87-1.73) | **2.69 (2.15-3.38)** |
|  | Parental educational attainment | High school diploma or less | 1.00 (Reference) | 1.00 (Reference) | 1.00 (Reference) | 1.00 (Reference) | 1.00 (Reference) | 1.00 (Reference) | 1.00 (Reference) | 1.00 (Reference) |
|  |  | Bachelor's degree or higher | 1.00 (0.96-1.05) | **1.16 (1.07-1.26)** | 0.94 (0.82-1.08) | 1.09 (0.98-1.23) | 1.00 (0.85-1.18) | 1.09 (0.97-1.22) | 1.17 (0.99-1.38) | 1.08 (0.97-1.20) |
|  | Academic achievement | Low | 1.16 (1.09-1.23) | **0.90 (0.83-0.98)** | 1.17 (0.99-1.38) | 1.05 (0.90-1.21) | 0.82 (0.67-1.00) | **0.86 (0.76-0.98)** | 1.20 (1.00-1.44) | 1.03 (0.90-1.17) |
|  |  | Middle | 1.00 (Reference) | 1.00 (Reference) | 1.00 (Reference) | 1.00 (Reference) | 1.00 (Reference) | 1.00 (Reference) | 1.00 (Reference) | 1.00 (Reference) |
|  |  | High | **1.48 (1.38-1.58)** | **1.35 (1.22-1.50)** | 1.14 (0.93-1.4) | **1.54 (1.29-1.83)** | **0.79 (0.62-0.99)** | **1.21 (1.04-1.41)** | 1.14 (0.92-1.41) | **1.38 (1.19-1.61)** |
|  | **Suicidal attempt** | | | | | | | | | |
|  | Sex | Male | 1.00 (Reference) | 1.00 (Reference) | 1.00 (Reference) | 1.00 (Reference) | 1.00 (Reference) | 1.00 (Reference) | 1.00 (Reference) | 1.00 (Reference) |
|  |  | Female | **1.76 (1.67-1.85)** | **1.97 (1.67-2.32)** | 1.14 (0.83-1.57) | **2.25 (1.72-2.96)** | 0.88 (0.60-1.31) | **1.99 (1.50-2.65)** | 0.92 (0.63-1.33) | **1.82 (1.44-2.31)** |
|  | Grade level | Middle school  (7-9^th^) | 1.00 (Reference) | 1.00 (Reference) | 1.00 (Reference) | 1.00 (Reference) | 1.00 (Reference) | 1.00 (Reference) | 1.00 (Reference) | 1.00 (Reference) |
|  |  | High school  (10-12^th^) | **0.76 (0.72-0.81)** | **0.73 (0.62-0.86)** | **1.44 (1.03-2.01)** | 1.05 (0.78-1.40) | 0.87 (0.58-1.29) | 0.91 (0.70-1.19) | 0.98 (0.68-1.42) | 0.89 (0.69-1.16) |
|  | Residential area | Rural | 1.00 (Reference) | 1.00 (Reference) | 1.00 (Reference) | 1.00 (Reference) | 1.00 (Reference) | 1.00 (Reference) | 1.00 (Reference) | 1.00 (Reference) |
|  |  | Urban | 1.06 (1.00-1.12) | 1.10 (0.94-1.28) | 0.95 (0.68-1.31) | 1.04 (0.78-1.39) | 1.01 (0.69-1.49) | 1.05 (0.81-1.36) | 1.09 (0.76-1.56) | 1.14 (0.89-1.48) |
|  | Recent alcohol consumption | No | 1.00 (Reference) | 1.00 (Reference) | 1.00 (Reference) | 1.00 (Reference) | 1.00 (Reference) | 1.00 (Reference) | 1.00 (Reference) | 1.00 (Reference) |
|  |  | Yes | **2.62 (2.48-2.77)** | **2.67 (2.24-3.17)** | 1.20 (0.82-1.76) | **3.20 (2.28-4.49)** | 0.96 (0.60-1.54) | **3.08 (2.23-4.26)** | 0.93 (0.61-1.41) | **2.86 (2.20-3.71)** |
|  | Smoking status | No | 1.00 (Reference) | 1.00 (Reference) | 1.00 (Reference) | 1.00 (Reference) | 1.00 (Reference) | 1.00 (Reference) | 1.00 (Reference) | 1.00 (Reference) |
|  |  | Yes | **3.26 (3.05-3.48)** | **3.45 (2.73-4.36)** | 1.48 (0.89-2.46) | **5.11 (3.26-8.02)** | 0.67 (0.35-1.27) | **3.40 (2.15-5.39)** | **1.82 (1.01-3.26)** | **6.17 (4.30-8.86)** |
|  | Parental educational attainment | High school diploma or less | 1.00 (Reference) | 1.00 (Reference) | 1.00 (Reference) | 1.00 (Reference) | 1.00 (Reference) | 1.00 (Reference) | 1.00 (Reference) | 1.00 (Reference) |
|  |  | Bachelor's degree or higher | **0.93 (0.88-0.98)** | 1.05 (0.88-1.24) | 1.04 (0.75-1.44) | 1.09 (0.83-1.44) | 1.00 (0.68-1.47) | 1.09 (0.83-1.43) | 0.78 (0.54-1.12) | 0.85 (0.67-1.09) |
|  | Academic achievement | Low | **0.86 (0.81-0.92)** | 0.71 (0.58-0.86) | 1.16 (0.78-1.71) | 0.82 (0.58-1.15) | 0.88 (0.55-1.41) | 0.72 (0.52-1.00) | 1.31 (0.85-2.00) | 0.94 (0.72-1.24) |
|  |  | Middle | 1.00 (Reference) | 1.00 (Reference) | 1.00 (Reference) | 1.00 (Reference) | 1.00 (Reference) | 1.00 (Reference) | 1.00 (Reference) | 1.00 (Reference) |
|  |  | High | **1.70 (1.59-1.82)** | **1.48 (1.21-1.81)** | 1.24 (0.82-1.88) | **1.84 (1.28-2.65)** | 0.79 (0.48-1.30) | **1.46 (1.04-2.03)** | 0.99 (0.63-1.57) | **1.45 (1.06-1.98)** |
| **Middle income** | **Perceived stress level** | | | | | | | | | |
|  | Sex | Male | 1.00 (Reference) | 1.00 (Reference) | 1.00 (Reference) | 1.00 (Reference) | 1.00 (Reference) | 1.00 (Reference) | 1.00 (Reference) | 1.00 (Reference) |
|  |  | Female | **1.68 (1.60-1.75)** | **2.03 (1.94-2.12)** | **0.88 (0.81-0.95)** | **1.78 (1.67-1.90)** | 1.06 (0.97-1.15) | **1.88 (1.77-1.99)** | **0.86 (0.79-0.93)** | **1.61 (1.52-1.71)** |
|  | Grade level | Middle school  (7-9^th^) | 1.00 (Reference) | 1.00 (Reference) | 1.00 (Reference) | 1.00 (Reference) | 1.00 (Reference) | 1.00 (Reference) | 1.00 (Reference) | 1.00 (Reference) |
|  |  | High school  (10-12^th^) | **1.30 (1.24-1.36)** | **1.17 (1.12-1.23)** | **1.14 (1.05-1.24)** | **1.33 (1.24-1.42)** | **0.90 (0.81-0.99)** | **1.19 (1.11-1.27)** | 0.92 (0.84-1.01) | **1.09 (1.02-1.16)** |
|  | Residential area | Rural | 1.00 (Reference) | 1.00 (Reference) | 1.00 (Reference) | 1.00 (Reference) | 1.00 (Reference) | 1.00 (Reference) | 1.00 (Reference) | 1.00 (Reference) |
|  |  | Urban | **1.09 (1.04-1.15)** | 1.03 (0.98-1.08) | 1.01 (0.93-1.1) | 1.04 (0.97-1.12) | 1.00 (0.90-1.11) | 1.04 (0.97-1.12) | 0.94 (0.85-1.04) | 0.98 (0.92-1.05) |
|  | Recent alcohol consumption | No | 1.00 (Reference) | 1.00 (Reference) | 1.00 (Reference) | 1.00 (Reference) | 1.00 (Reference) | 1.00 (Reference) | 1.00 (Reference) | 1.00 (Reference) |
|  |  | Yes | **1.40 (1.31-1.50)** | **1.38 (1.31-1.45)** | **0.82 (0.72-0.93)** | **1.64 (1.50-1.79)** | **0.82 (0.72-0.93)** | **1.34 (1.22-1.47)** | 1.03 (0.91-1.17) | **1.38 (1.27-1.51)** |
|  | Smoking status | No | 1.00 (Reference) | 1.00 (Reference) | 1.00 (Reference) | 1.00 (Reference) | 1.00 (Reference) | 1.00 (Reference) | 1.00 (Reference) | 1.00 (Reference) |
|  |  | Yes | **1.56 (1.37-1.77)** | **1.23 (1.12-1.36)** | 1.11 (0.89-1.37) | **1.36 (1.12-1.64)** | 1.03 (0.80-1.33) | **1.40 (1.18-1.66)** | 1.08 (0.86-1.36) | **1.51 (1.30-1.76)** |
|  | Parental educational attainment | High school diploma or less | 1.00 (Reference) | 1.00 (Reference) | 1.00 (Reference) | 1.00 (Reference) | 1.00 (Reference) | 1.00 (Reference) | 1.00 (Reference) | 1.00 (Reference) |
|  |  | Bachelor's degree or higher | **1.05 (1.01-1.10)** | **1.07 (1.03-1.11)** | 0.94 (0.87-1.00) | 1.00 (0.94-1.06) | 1.07 (0.99-1.16) | **1.07 (1.01-1.13)** | 0.93 (0.85-1.00) | 0.99 (0.94-1.06) |
|  | Academic achievement | Low | 0.97 (0.92-1.02) | 1.03 (0.98-1.08) | 1.00 (0.91-1.10) | 1.03 (0.96-1.12) | 1.03 (0.93-1.14) | 1.06 (0.99-1.13) | 1.00 (0.91-1.10) | 1.06 (0.99-1.14) |
|  |  | Middle | 1.00 (Reference) | 1.00 (Reference) | 1.00 (Reference) | 1.00 (Reference) | 1.00 (Reference) | 1.00 (Reference) | 1.00 (Reference) | 1.00 (Reference) |
|  |  | High | **1.24 (1.17-1.31)** | **1.26 (1.21-1.32)** | 1.05 (0.96-1.14) | **1.32 (1.23-1.42)** | 0.92 (0.83-1.01) | **1.21 (1.14-1.30)** | 1.03 (0.94-1.14) | **1.25 (1.16-1.33)** |
|  | **sadness** | | | | | | | | | |
|  | Sex | Male | 1.00 (Reference) | 1.00 (Reference) | 1.00 (Reference) | 1.00 (Reference) | 1.00 (Reference) | 1.00 (Reference) | 1.00 (Reference) | 1.00 (Reference) |
|  |  | Female | **1.63 (1.56-1.71)** | **1.90 (1.81-1.99)** | 0.95 (0.87-1.03) | **1.80 (1.68-1.93)** | 0.93 (0.85-1.03) | **1.68 (1.57-1.79)** | 0.95 (0.86-1.04) | **1.59 (1.49-1.70)** |
|  | Grade level | Middle school  (7-9^th^) | 1.00 (Reference) | 1.00 (Reference) | 1.00 (Reference) | 1.00 (Reference) | 1.00 (Reference) | 1.00 (Reference) | 1.00 (Reference) | 1.00 (Reference) |
|  |  | High school  (10-12^th^) | **1.16 (1.11-1.22)** | **1.08 (1.03-1.14)** | 1.09 (1.00-1.20) | **1.18 (1.09-1.27)** | 0.92 (0.82-1.02) | **1.08 (1.01-1.17)** | 0.94 (0.85-1.03) | 1.01 (0.95-1.09) |
|  | Residential area | Rural | 1.00 (Reference) | 1.00 (Reference) | 1.00 (Reference) | 1.00 (Reference) | 1.00 (Reference) | 1.00 (Reference) | 1.00 (Reference) | 1.00 (Reference) |
|  |  | Urban | 1.04 (0.99-1.10) | 1.04 (0.99-1.10) | 1.04 (0.95-1.14) | 1.08 (1.00-1.16) | 1.04 (0.94-1.15) | **1.12 (1.04-1.20)** | 0.91 (0.83-1.01) | 1.02 (0.95-1.09) |
|  | Recent alcohol consumption | No | 1.00 (Reference) | 1.00 (Reference) | 1.00 (Reference) | 1.00 (Reference) | 1.00 (Reference) | 1.00 (Reference) | 1.00 (Reference) | 1.00 (Reference) |
|  |  | Yes | **1.98 (1.85-2.12)** | **1.94 (1.83-2.05)** | 1.12 (1.00-1.25) | **2.17 (1.97-2.39)** | 0.92 (0.80-1.05) | **1.99 (1.81-2.18)** | 0.92 (0.81-1.05) | **1.83 (1.67-2.00)** |
|  | Smoking status | No | 1.00 (Reference) | 1.00 (Reference) | 1.00 (Reference) | 1.00 (Reference) | 1.00 (Reference) | 1.00 (Reference) | 1.00 (Reference) | 1.00 (Reference) |
|  |  | Yes | **2.50 (2.21-2.83)** | **1.92 (1.73-2.14)** | **1.40 (1.11-1.77)** | **2.69 (2.18-3.32)** | 0.82 (0.62-1.08) | **2.20 (1.84-2.64)** | 1.12 (0.88-1.43) | **2.46 (2.09-2.89)** |
|  | Parental educational attainment | High school diploma or less | 1.00 (Reference) | 1.00 (Reference) | 1.00 (Reference) | 1.00 (Reference) | 1.00 (Reference) | 1.00 (Reference) | 1.00 (Reference) | 1.00 (Reference) |
|  |  | Bachelor's degree or higher | 1.03 (0.99-1.09) | **1.16 (1.11-1.21)** | **0.9 (0.83-0.97)** | 1.04 (0.98-1.11) | 0.99 (0.91-1.08) | 1.03 (0.97-1.09) | 0.97 (0.89-1.06) | 1.00 (0.95-1.07) |
|  | Academic achievement | Low | **0.83 (0.78-0.87)** | **0.92 (0.87-0.97)** | 1.01 (0.92-1.11) | 0.93 (0.86-1.01) | 1.01 (0.90-1.13) | 0.94 (0.87-1.02) | 1.03 (0.92-1.16) | 0.97 (0.89-1.05) |
|  |  | Middle | 1.00 (Reference) | 1.00 (Reference) | 1.00 (Reference) | 1.00 (Reference) | 1.00 (Reference) | 1.00 (Reference) | 1.00 (Reference) | 1.00 (Reference) |
|  |  | High | **1.33 (1.25-1.42)** | **1.32 (1.26-1.39)** | 1.05 (0.96-1.14) | **1.38 (1.29-1.48)** | 1.00 (0.91-1.11) | **1.38 (1.28-1.48)** | 1.01 (0.91-1.12) | **1.39 (1.29-1.50)** |
|  | **Suicidal ideation** | | | | | | | | | |
|  | Sex | Male | 1.00 (Reference) | 1.00 (Reference) | 1.00 (Reference) | 1.00 (Reference) | 1.00 (Reference) | 1.00 (Reference) | 1.00 (Reference) | 1.00 (Reference) |
|  |  | Female | **1.65 (1.60-1.69)** | **2.05 (1.92-2.18)** | **0.87 (0.77-0.99)** | **1.79 (1.61-1.99)** | 1.07 (0.93-1.23) | **1.91 (1.74-2.10)** | 0.95 (0.83-1.08) | **1.81 (1.65-1.97)** |
|  | Grade level | Middle school  (7-9^th^) | 1.00 (Reference) | 1.00 (Reference) | 1.00 (Reference) | 1.00 (Reference) | 1.00 (Reference) | 1.00 (Reference) | 1.00 (Reference) | 1.00 (Reference) |
|  |  | High school  (10-12^th^) | **0.96 (0.93-0.98)** | **0.78 (0.73-0.83)** | **1.41 (1.25-1.59)** | 1.10 (0.99-1.22) | **0.74 (0.64-0.85)** | **0.81 (0.73-0.89)** | 0.95 (0.84-1.08) | **0.77 (0.71-0.84)** |
|  | Residential area | Rural | 1.00 (Reference) | 1.00 (Reference) | 1.00 (Reference) | 1.00 (Reference) | 1.00 (Reference) | 1.00 (Reference) | 1.00 (Reference) | 1.00 (Reference) |
|  |  | Urban | **1.08 (1.05-1.11)** | 1.01 (0.95-1.08) | 1.11 (0.98-1.26) | **1.12 (1.01-1.25)** | 1.00 (0.86-1.16) | **1.12 (1.01-1.24)** | 0.93 (0.81-1.07) | 1.04 (0.95-1.14) |
|  | Recent alcohol consumption | No | 1.00 (Reference) | 1.00 (Reference) | 1.00 (Reference) | 1.00 (Reference) | 1.00 (Reference) | 1.00 (Reference) | 1.00 (Reference) | 1.00 (Reference) |
|  |  | Yes | **1.91 (1.86-1.97)** | **1.68 (1.57-1.81)** | **1.30 (1.12-1.50)** | **2.18 (1.92-2.48)** | **0.79 (0.66-0.94)** | **1.72 (1.52-1.94)** | 1.02 (0.86-1.21) | **1.76 (1.57-1.98)** |
|  | Smoking status | No | 1.00 (Reference) | 1.00 (Reference) | 1.00 (Reference) | 1.00 (Reference) | 1.00 (Reference) | 1.00 (Reference) | 1.00 (Reference) | 1.00 (Reference) |
|  |  | Yes | **2.08 (2.00-2.17)** | **1.64 (1.45-1.85)** | **1.67 (1.27-2.20)** | **2.74 (2.14-3.51)** | 0.76 (0.55-1.05) | **2.08 (1.69-2.56)** | **1.10 (0.83-1.46)** | **2.28 (1.87-2.76)** |
|  | Parental educational attainment | High school diploma or less | 1.00 (Reference) | 1.00 (Reference) | 1.00 (Reference) | 1.00 (Reference) | 1.00 (Reference) | 1.00 (Reference) | 1.00 (Reference) | 1.00 (Reference) |
|  |  | Bachelor's degree or higher | **1.08 (1.06-1.11)** | **1.19 (1.12-1.26)** | **0.89 (0.80-0.99)** | 1.06 (0.97-1.16) | 1.1 (0.98-1.25) | **1.17 (1.08-1.27)** | 0.93 (0.83-1.04) | **1.09 (1.01-1.18)** |
|  | Academic achievement | Low | 0.97 (0.94-1.00) | 1.03 (0.95-1.10) | 1.01 (0.88-1.16) | 1.04 (0.93-1.17) | 1.02 (0.87-1.19) | 1.06 (0.96-1.18) | 1.14 (0.99-1.32) | **1.21 (1.09-1.34)** |
|  |  | Middle | 1.00 (Reference) | 1.00 (Reference) | 1.00 (Reference) | 1.00 (Reference) | 1.00 (Reference) | 1.00 (Reference) | 1.00 (Reference) | 1.00 (Reference) |
|  |  | High | **1.43 (1.38-1.48)** | **1.37 (1.28-1.46)** | 1.02 (0.91-1.15) | **1.40 (1.26-1.54)** | 1.00 (0.87-1.14) | **1.40 (1.28-1.53)** | 1.05 (0.92-1.19) | **1.47 (1.34-1.61)** |
|  | **Suicidal attempt** | | | | | | | | | |
|  | Sex | Male | 1.00 (Reference) | 1.00 (Reference) | 1.00 (Reference) | 1.00 (Reference) | 1.00 (Reference) | 1.00 (Reference) | 1.00 (Reference) | 1.00 (Reference) |
|  |  | Female | **1.99 (1.71-2.32)** | **2.41 (2.09-2.77)** | 0.93 (0.72-1.21) | **2.25 (1.81-2.80)** | 0.88 (0.66-1.19) | **1.99 (1.63-2.44)** | 0.95 (0.71-1.25) | **1.88 (1.54-2.29)** |
|  | Grade level | Middle school  (7-9^th^) | 1.00 (Reference) | 1.00 (Reference) | 1.00 (Reference) | 1.00 (Reference) | 1.00 (Reference) | 1.00 (Reference) | 1.00 (Reference) | 1.00 (Reference) |
|  |  | High school  (10-12^th^) | 0.94 (0.80-1.10) | **0.55 (0.49-0.63)** | **1.73 (1.36-2.2)** | 0.95 (0.77-1.16) | 0.77 (0.58-1.03) | **0.73 (0.60-0.90)** | 0.80 (0.60-1.05) | **0.58 (0.48-0.71)** |
|  | Residential area | Rural | 1.00 (Reference) | 1.00 (Reference) | 1.00 (Reference) | 1.00 (Reference) | 1.00 (Reference) | 1.00 (Reference) | 1.00 (Reference) | 1.00 (Reference) |
|  |  | Urban | 0.95 (0.80-1.12) | 1.04 (0.92-1.18) | 1.04 (0.82-1.32) | 1.08 (0.88-1.33) | 1.07 (0.80-1.44) | 1.16 (0.94-1.42) | 1.08 (0.81-1.44) | **1.25 (1.03-1.53)** |
|  | Recent alcohol consumption | No | 1.00 (Reference) | 1.00 (Reference) | 1.00 (Reference) | 1.00 (Reference) | 1.00 (Reference) | 1.00 (Reference) | 1.00 (Reference) | 1.00 (Reference) |
|  |  | Yes | **3.05 (2.56-3.63)** | **2.79 (2.47-3.15)** | 1.13 (0.85-1.48) | **3.14 (2.45-4.02)** | 0.76 (0.54-1.08) | **2.40 (1.88-3.06)** | 1.17 (0.85-1.62) | **2.81 (2.27-3.49)** |
|  | Smoking status | No | 1.00 (Reference) | 1.00 (Reference) | 1.00 (Reference) | 1.00 (Reference) | 1.00 (Reference) | 1.00 (Reference) | 1.00 (Reference) | 1.00 (Reference) |
|  |  | Yes | **5.06 (3.98-6.43)** | **3.39 (2.86-4.03)** | 1.35 (0.86-2.1) | **4.57 (3.03-6.89)** | 0.78 (0.46-1.32) | **3.58 (2.58-4.97)** | 1.34 (0.85-2.1) | **4.79 (3.53-6.51)** |
|  | Parental educational attainment | High school diploma or less | 1.00 (Reference) | 1.00 (Reference) | 1.00 (Reference) | 1.00 (Reference) | 1.00 (Reference) | 1.00 (Reference) | 1.00 (Reference) | 1.00 (Reference) |
|  |  | Bachelor's degree or higher | 1.00 (0.86-1.16) | 0.89 (0.79-0.99) | 1.12 (0.89-1.42) | 1.00 (0.81-1.22) | 0.85 (0.64-1.13) | 0.85 (0.70-1.04) | 1.12 (0.85-1.46) | 0.95 (0.79-1.14) |
|  | Academic achievement | Low | 0.84 (0.70-1.00) | 1.03 (0.88-1.19) | 0.9 (0.66-1.24) | 0.93 (0.70-1.23) | 0.86 (0.58-1.28) | 0.80 (0.60-1.05) | 1.29 (0.87-1.9) | 1.03 (0.79-1.35) |
|  |  | Middle | 1.00 (Reference) | 1.00 (Reference) | 1.00 (Reference) | 1.00 (Reference) | 1.00 (Reference) | 1.00 (Reference) | 1.00 (Reference) | 1.00 (Reference) |
|  |  | High | **1.57 (1.29-1.90)** | **1.67 (1.46-1.91)** | 0.99 (0.75-1.31) | **1.66 (1.30-2.11)** | 0.89 (0.65-1.23) | **1.48 (1.19-1.83)** | 1.19 (0.87-1.63) | **1.76 (1.39-2.22)** |
| **Mid-low income** | **Perceived stress level** | | | | | | | | | |
|  | Sex | Male | 1.00 (Reference) | 1.00 (Reference) | 1.00 (Reference) | 1.00 (Reference) | 1.00 (Reference) | 1.00 (Reference) | 1.00 (Reference) | 1.00 (Reference) |
|  |  | Female | **1.84 (1.80-1.89)** | **2.20 (2.03-2.38)** | 0.88 (0.76-1.01) | **1.93 (1.72-2.16)** | 0.89 (0.75-1.06) | **1.72 (1.52-1.95)** | 0.92 (0.77-1.1) | **1.58 (1.38-1.80)** |
|  | Grade level | Middle school  (7-9^th^) | 1.00 (Reference) | 1.00 (Reference) | 1.00 (Reference) | 1.00 (Reference) | 1.00 (Reference) | 1.00 (Reference) | 1.00 (Reference) | 1.00 (Reference) |
|  |  | High school  (10-12^th^) | 1.01 (0.99-1.04) | 1.00 (0.92-1.09) | 0.92 (0.79-1.07) | 0.92 (0.81-1.04) | 1.02 (0.86-1.22) | 0.94 (0.83-1.06) | 0.94 (0.78-1.12) | 0.88 (0.77-1.00) |
|  | Residential area | Rural | 1.00 (Reference) | 1.00 (Reference) | 1.00 (Reference) | 1.00 (Reference) | 1.00 (Reference) | 1.00 (Reference) | 1.00 (Reference) | 1.00 (Reference) |
|  |  | Urban | 1.00 (0.98-1.03) | 1.02 (0.94-1.11) | 1.02 (0.88-1.19) | 1.04 (0.92-1.18) | 1.14 (0.95-1.35) | **1.18 (1.04-1.33)** | 0.75 (0.63-0.9) | 0.89 (0.78-1.01) |
|  | Recent alcohol consumption | No | 1.00 (Reference) | 1.00 (Reference) | 1.00 (Reference) | 1.00 (Reference) | 1.00 (Reference) | 1.00 (Reference) | 1.00 (Reference) | 1.00 (Reference) |
|  |  | Yes | **1.32 (1.29-1.36)** | **1.27 (1.15-1.41)** | 1.16 (0.95-1.41) | **1.47 (1.24-1.74)** | 0.92 (0.72-1.17) | **1.35 (1.14-1.60)** | 0.84 (0.66-1.08) | 1.14 (0.95-1.36) |
|  | Smoking status | No | 1.00 (Reference) | 1.00 (Reference) | 1.00 (Reference) | 1.00 (Reference) | 1.00 (Reference) | 1.00 (Reference) | 1.00 (Reference) | 1.00 (Reference) |
|  |  | Yes | **1.26 (1.21-1.30)** | 1.03 (0.88-1.19) | 1.29 (0.87-1.91) | 1.33 (0.93-1.91) | 1.17 (0.73-1.86) | **1.55 (1.15-2.09)** | 0.74 (0.48-1.13) | 1.14 (0.84-1.54) |
|  | Parental educational attainment | High school diploma or less | 1.00 (Reference) | 1.00 (Reference) | 1.00 (Reference) | 1.00 (Reference) | 1.00 (Reference) | 1.00 (Reference) | 1.00 (Reference) | 1.00 (Reference) |
|  |  | Bachelor's degree or higher | **1.04 (1.01-1.06)** | 1.01 (0.93-1.09) | 1.08 (0.94-1.25) | 1.09 (0.96-1.22) | 0.93 (0.78-1.1) | 1.01 (0.89-1.14) | 1.04 (0.87-1.24) | 1.05 (0.92-1.19) |
|  | Academic achievement | Low | 0.98 (0.95-1.01) | 0.95 (0.86-1.06) | 0.98 (0.81-1.19) | 0.93 (0.79-1.10) | 1.13 (0.88-1.44) | 1.05 (0.88-1.26) | 1.02 (0.79-1.32) | 1.07 (0.89-1.28) |
|  |  | Middle | 1.00 (Reference) | 1.00 (Reference) | 1.00 (Reference) | 1.00 (Reference) | 1.00 (Reference) | 1.00 (Reference) | 1.00 (Reference) | 1.00 (Reference) |
|  |  | High | **1.16 (1.13-1.20)** | **1.13 (1.03-1.24)** | 1.05 (0.9-1.24) | **1.19 (1.04-1.36)** | 0.97 (0.79-1.18) | 1.15 (0.99-1.33) | 1.11 (0.89-1.39) | **1.28 (1.08-1.50)** |
|  | **sadness** | | | | | | | | | |
|  | Sex | Male | 1.00 (Reference) | 1.00 (Reference) | 1.00 (Reference) | 1.00 (Reference) | 1.00 (Reference) | 1.00 (Reference) | 1.00 (Reference) | 1.00 (Reference) |
|  |  | Female | **1.72 (1.68-1.77)** | **1.94 (1.79-2.11)** | 0.91 (0.78-1.06) | **1.76 (1.55-2.01)** | 1.01 (0.84-1.22) | **1.78 (1.56-2.03)** | 0.92 (0.77-1.11) | **1.64 (1.44-1.87)** |
|  | Grade level | Middle school  (7-9^th^) | 1.00 (Reference) | 1.00 (Reference) | 1.00 (Reference) | 1.00 (Reference) | 1.00 (Reference) | 1.00 (Reference) | 1.00 (Reference) | 1.00 (Reference) |
|  |  | High school  (10-12^th^) | 1.00 (0.97-1.02) | 0.93 (0.85-1.02) | 1.03 (0.89-1.20) | 0.96 (0.85-1.09) | 0.94 (0.78-1.13) | 0.90 (0.79-1.03) | 0.89 (0.74-1.07) | 0.80 (0.70-0.91) |
|  | Residential area | Rural | 1.00 (Reference) | 1.00 (Reference) | 1.00 (Reference) | 1.00 (Reference) | 1.00 (Reference) | 1.00 (Reference) | 1.00 (Reference) | 1.00 (Reference) |
|  |  | Urban | 1.02 (1.00-1.05) | 0.99 (0.91-1.09) | 1.1 (0.94-1.29) | 1.09 (0.96-1.24) | 1.02 (0.85-1.23) | 1.11 (0.97-1.27) | 0.88 (0.73-1.07) | 0.98 (0.85-1.12) |
|  | Recent alcohol consumption | No | 1.00 (Reference) | 1.00 (Reference) | 1.00 (Reference) | 1.00 (Reference) | 1.00 (Reference) | 1.00 (Reference) | 1.00 (Reference) | 1.00 (Reference) |
|  |  | Yes | **1.78 (1.73-1.83)** | **1.75 (1.59-1.93)** | 1.04 (0.86-1.25) | **1.82 (1.55-2.13)** | 1.14 (0.89-1.45) | **2.07 (1.73-2.48)** | **0.77 (0.60-0.98)** | **1.59 (1.34-1.88)** |
|  | Smoking status | No | 1.00 (Reference) | 1.00 (Reference) | 1.00 (Reference) | 1.00 (Reference) | 1.00 (Reference) | 1.00 (Reference) | 1.00 (Reference) | 1.00 (Reference) |
|  |  | Yes | **1.76 (1.70-1.83)** | **1.55 (1.33-1.79)** | **1.48 (1.03-2.13)** | **2.29 (1.64-3.19)** | 1.15 (0.74-1.79) | **2.64 (1.98-3.53)** | 0.91 (0.60-1.39) | **2.41 (1.78-3.27)** |
|  | Parental educational attainment | High school diploma or less | 1.00 (Reference) | 1.00 (Reference) | 1.00 (Reference) | 1.00 (Reference) | 1.00 (Reference) | 1.00 (Reference) | 1.00 (Reference) | 1.00 (Reference) |
|  |  | Bachelor's degree or higher | **1.08 (1.05-1.11)** | 1.08 (0.99-1.17) | 1.07 (0.92-1.23) | **1.15 (1.02-1.30)** | **0.84 (0.70-0.99)** | 0.96 (0.85-1.09) | 1.17 (0.98-1.39) | 1.12 (0.99-1.27) |
|  | Academic achievement | Low | **0.91 (0.88-0.94)** | 0.89 (0.80-1.00) | 1.12 (0.92-1.38) | 1.00 (0.84-1.18) | 0.94 (0.73-1.22) | 0.94 (0.77-1.14) | 1.21 (0.93-1.58) | 1.14 (0.95-1.36) |
|  |  | Middle | 1.00 (Reference) | 1.00 (Reference) | 1.00 (Reference) | 1.00 (Reference) | 1.00 (Reference) | 1.00 (Reference) | 1.00 (Reference) | 1.00 (Reference) |
|  |  | High | **1.21 (1.17-1.24)** | **1.18 (1.06-1.30)** | 0.98 (0.81-1.17) | 1.15 (0.99-1.34) | 1.04 (0.83-1.29) | **1.19 (1.02-1.40)** | 1.19 (0.95-1.48) | **1.41 (1.20-1.65)** |
|  | **Suicidal ideation** | | | | | | | | | |
|  | Sex | Male | 1.00 (Reference) | 1.00 (Reference) | 1.00 (Reference) | 1.00 (Reference) | 1.00 (Reference) | 1.00 (Reference) | 1.00 (Reference) | 1.00 (Reference) |
|  |  | Female | **1.89 (1.83-1.95)** | **2.08 (1.88-2.30)** | 0.95 (0.79-1.15) | **1.98 (1.69-2.31)** | 1.07 (0.86-1.34) | **2.12 (1.81-2.48)** | 0.89 (0.71-1.11) | **1.88 (1.61-2.19)** |
|  | Grade level | Middle school  (7-9^th^) | 1.00 (Reference) | 1.00 (Reference) | 1.00 (Reference) | 1.00 (Reference) | 1.00 (Reference) | 1.00 (Reference) | 1.00 (Reference) | 1.00 (Reference) |
|  |  | High school  (10-12^th^) | **0.77 (0.74-0.79)** | **0.68 (0.61-0.75)** | 1.10 (0.91-1.33) | **0.75 (0.64-0.88)** | 0.97 (0.77-1.22) | **0.73 (0.62-0.86)** | 0.99 (0.79-1.24) | **0.72 (0.62-0.85)** |
|  | Residential area | Rural | 1.00 (Reference) | 1.00 (Reference) | 1.00 (Reference) | 1.00 (Reference) | 1.00 (Reference) | 1.00 (Reference) | 1.00 (Reference) | 1.00 (Reference) |
|  |  | Urban | 1.02 (0.99-1.05) | 0.97 (0.88-1.08) | 1.08 (0.89-1.31) | 1.05 (0.89-1.23) | 1.10 (0.87-1.38) | 1.15 (0.97-1.36) | 0.95 (0.75-1.20) | 1.09 (0.93-1.28) |
|  | Recent alcohol consumption | No | 1.00 (Reference) | 1.00 (Reference) | 1.00 (Reference) | 1.00 (Reference) | 1.00 (Reference) | 1.00 (Reference) | 1.00 (Reference) | 1.00 (Reference) |
|  |  | Yes | **1.61 (1.56-1.66)** | **1.77 (1.57-1.99)** | 1.01 (0.81-1.25) | **1.78 (1.49-2.14)** | 1.07 (0.81-1.40) | **1.90 (1.55-2.32)** | 0.83 (0.62-1.09) | **1.57 (1.29-1.91)** |
|  | Smoking status | No | 1.00 (Reference) | 1.00 (Reference) | 1.00 (Reference) | 1.00 (Reference) | 1.00 (Reference) | 1.00 (Reference) | 1.00 (Reference) | 1.00 (Reference) |
|  |  | Yes | **1.63 (1.56-1.70)** | **1.69 (1.42-2.00)** | 1.34 (0.88-2.03) | **2.26 (1.54-3.30)** | 1.24 (0.76-2.02) | **2.79 (2.04-3.81)** | 0.82 (0.53-1.27) | **2.30 (1.70-3.10)** |
|  | Parental educational attainment | High school diploma or less | 1.00 (Reference) | 1.00 (Reference) | 1.00 (Reference) | 1.00 (Reference) | 1.00 (Reference) | 1.00 (Reference) | 1.00 (Reference) | 1.00 (Reference) |
|  |  | Bachelor's degree or higher | **1.06 (1.03-1.09)** | 1.07 (0.97-1.18) | 1.17 (0.98-1.40) | **1.25 (1.07-1.45)** | 0.93 (0.75-1.15) | 1.16 (1.00-1.34) | 0.85 (0.68-1.06) | 0.99 (0.84-1.17) |
|  | Academic achievement | Low | 0.97 (0.93-1.01) | 0.88 (0.76-1.01) | 1.19 (0.93-1.53) | 1.05 (0.86-1.29) | 0.99 (0.72-1.35) | 1.04 (0.82-1.32) | 0.92 (0.67-1.28) | 0.96 (0.77-1.21) |
|  |  | Middle | 1.00 (Reference) | 1.00 (Reference) | 1.00 (Reference) | 1.00 (Reference) | 1.00 (Reference) | 1.00 (Reference) | 1.00 (Reference) | 1.00 (Reference) |
|  |  | High | **1.21 (1.17-1.25)** | 1.13 (1.00-1.27) | 1.02 (0.82-1.26) | 1.15 (0.96-1.37) | 1.00 (0.78-1.29) | 1.15 (0.96-1.38) | 1.1 (0.84-1.43) | **1.26 (1.03-1.53)** |
|  | **Suicidal attempt** | | | | | | | | | |
|  | Sex | Male | 1.00 (Reference) | 1.00 (Reference) | 1.00 (Reference) | 1.00 (Reference) | 1.00 (Reference) | 1.00 (Reference) | 1.00 (Reference) | 1.00 (Reference) |
|  |  | Female | **2.15 (2.02-2.28)** | **2.39 (1.98-2.87)** | 1.00 (0.67-1.49) | **2.39 (1.68-3.40)** | 1.11 (0.69-1.81) | **2.66 (1.91-3.70)** | 0.77 (0.48-1.25) | **2.06 (1.45-2.92)** |
|  | Grade level | Middle school  (7-9^th^) | 1.00 (Reference) | 1.00 (Reference) | 1.00 (Reference) | 1.00 (Reference) | 1.00 (Reference) | 1.00 (Reference) | 1.00 (Reference) | 1.00 (Reference) |
|  |  | High school  (10-12^th^) | **0.60 (0.57-0.63)** | **0.52 (0.44-0.62)** | 1.27 (0.90-1.80) | **0.66 (0.49-0.90)** | 1.03 (0.66-1.61) | **0.68 (0.49-0.94)** | 0.79 (0.49-1.28) | **0.54 (0.38-0.76)** |
|  | Residential area | Rural | 1.00 (Reference) | 1.00 (Reference) | 1.00 (Reference) | 1.00 (Reference) | 1.00 (Reference) | 1.00 (Reference) | 1.00 (Reference) | 1.00 (Reference) |
|  |  | Urban | 1.02 (0.97-1.09) | 1.02 (0.86-1.22) | 1.00 (0.70-1.43) | 1.02 (0.75-1.39) | 1.16 (0.73-1.83) | 1.18 (0.84-1.65) | 0.77 (0.48-1.24) | 0.91 (0.65-1.28) |
|  | Recent alcohol consumption | No | 1.00 (Reference) | 1.00 (Reference) | 1.00 (Reference) | 1.00 (Reference) | 1.00 (Reference) | 1.00 (Reference) | 1.00 (Reference) | 1.00 (Reference) |
|  |  | Yes | **2.27 (2.15-2.40)** | **2.19 (1.83-2.62)** | 1.26 (0.85-1.86) | **2.75 (1.94-3.88)** | 1.07 (0.67-1.73) | **2.95 (2.12-4.10)** | 0.73 (0.44-1.2) | **2.15 (1.48-3.12)** |
|  | Smoking status | No | 1.00 (Reference) | 1.00 (Reference) | 1.00 (Reference) | 1.00 (Reference) | 1.00 (Reference) | 1.00 (Reference) | 1.00 (Reference) | 1.00 (Reference) |
|  |  | Yes | **2.60 (2.43-2.79)** | **2.43 (1.93-3.06)** | **1.99 (1.12-3.52)** | **4.83 (2.87-8.14)** | 0.95 (0.47-1.92) | **4.57 (2.83-7.37)** | 0.88 (0.45-1.70) | **4.01 (2.54-6.33)** |
|  | Parental educational attainment | High school diploma or less | 1.00 (Reference) | 1.00 (Reference) | 1.00 (Reference) | 1.00 (Reference) | 1.00 (Reference) | 1.00 (Reference) | 1.00 (Reference) | 1.00 (Reference) |
|  |  | Bachelor's degree or higher | 1.00 (0.95-1.06) | 0.96 (0.80-1.16) | **1.46 (1.03-2.06)** | **1.40 (1.05-1.88)** | 0.66 (0.42-1.03) | 0.92 (0.65-1.29) | 0.94 (0.58-1.51) | 0.86 (0.62-1.20) |
|  | Academic achievement | Low | 0.90 (0.83-0.98) | 1.03 (0.78-1.38) | 0.95 (0.55-1.64) | 0.98 (0.62-1.57) | 1.06 (0.55-2.06) | 1.04 (0.65-1.68) | 0.76 (0.38-1.52) | 0.79 (0.48-1.32) |
|  |  | Middle | 1.00 (Reference) | 1.00 (Reference) | 1.00 (Reference) | 1.00 (Reference) | 1.00 (Reference) | 1.00 (Reference) | 1.00 (Reference) | 1.00 (Reference) |
|  |  | High | **1.31 (1.23-1.41)** | **1.50 (1.20-1.87)** | 0.93 (0.6-1.44) | 1.39 (0.95-2.03) | 0.90 (0.52-1.55) | 1.25 (0.85-1.84) | 0.94 (0.55-1.64) | 1.18 (0.80-1.75) |
| **Low income** | **Perceived stress level** | | | | | | | | | |
|  | Sex | Male | 1.00 (Reference) | 1.00 (Reference) | 1.00 (Reference) | 1.00 (Reference) | 1.00 (Reference) | 1.00 (Reference) | 1.00 (Reference) | 1.00 (Reference) |
|  |  | Female | **1.89 (1.80-1.97)** | **2.04 (1.70-2.44)** | 0.9 (0.66-1.22) | **1.83 (1.43-2.36)** | 1.18 (0.81-1.7) | **2.15 (1.64-2.82)** | 0.69 (0.47-1.03) | **1.49 (1.11-1.99)** |
|  | Grade level | Middle school  (7-9^th^) | 1.00 (Reference) | 1.00 (Reference) | 1.00 (Reference) | 1.00 (Reference) | 1.00 (Reference) | 1.00 (Reference) | 1.00 (Reference) | 1.00 (Reference) |
|  |  | High school  (10-12^th^) | **1.08 (1.03-1.13)** | 1.00 (0.83-1.20) | 1.22 (0.89-1.68) | 1.22 (0.94-1.58) | 0.77 (0.53-1.13) | 0.94 (0.71-1.24) | 1.04 (0.70-1.56) | 0.98 (0.73-1.31) |
|  | Residential area | Rural | 1.00 (Reference) | 1.00 (Reference) | 1.00 (Reference) | 1.00 (Reference) | 1.00 (Reference) | 1.00 (Reference) | 1.00 (Reference) | 1.00 (Reference) |
|  |  | Urban | 1.04 (1.00-1.09) | 1.04 (0.87-1.25) | 1.13 (0.83-1.53) | 1.17 (0.91-1.50) | 0.86 (0.59-1.23) | 1.00 (0.76-1.30) | 1.12 (0.75-1.66) | 1.12 (0.84-1.50) |
|  | Recent alcohol consumption | No | 1.00 (Reference) | 1.00 (Reference) | 1.00 (Reference) | 1.00 (Reference) | 1.00 (Reference) | 1.00 (Reference) | 1.00 (Reference) | 1.00 (Reference) |
|  |  | Yes | **1.34 (1.28-1.40)** | **1.33 (1.11-1.61)** | 1.23 (0.86-1.76) | **1.63 (1.20-2.22)** | 0.88 (0.57-1.37) | **1.44 (1.05-1.98)** | 0.99 (0.62-1.58) | 1.42 (1.00-2.00) |
|  | Smoking status | No | 1.00 (Reference) | 1.00 (Reference) | 1.00 (Reference) | 1.00 (Reference) | 1.00 (Reference) | 1.00 (Reference) | 1.00 (Reference) | 1.00 (Reference) |
|  |  | Yes | **1.26 (1.20-1.33)** | 1.06 (0.83-1.36) | 1.81 (1.00-3.27) | **1.92 (1.12-3.28)** | 0.63 (0.30-1.29) | 1.20 (0.73-1.95) | 1.13 (0.58-2.19) | 1.35 (0.86-2.11) |
|  | Parental educational attainment | High school diploma or less | 1.00 (Reference) | 1.00 (Reference) | 1.00 (Reference) | 1.00 (Reference) | 1.00 (Reference) | 1.00 (Reference) | 1.00 (Reference) | 1.00 (Reference) |
|  |  | Bachelor's degree or higher | **1.06 (1.01-1.11)** | 1.07 (0.89-1.29) | 1.11 (0.80-1.55) | 1.19 (0.90-1.56) | 1.34 (0.88-2.03) | **1.59 (1.16-2.18)** | 0.62 (0.40-0.97) | 0.99 (0.73-1.34) |
|  | Academic achievement | Low | **0.92 (0.85-0.99)** | 0.98 (0.75-1.29) | 1.00 (0.62-1.63) | 0.98 (0.66-1.48) | 1.39 (0.76-2.54) | 1.36 (0.87-2.13) | 0.59 (0.30-1.16) | 0.80 (0.48-1.32) |
|  |  | Middle | 1.00 (Reference) | 1.00 (Reference) | 1.00 (Reference) | 1.00 (Reference) | 1.00 (Reference) | 1.00 (Reference) | 1.00 (Reference) | 1.00 (Reference) |
|  |  | High | **1.10 (1.04-1.17)** | 1.24 (0.98-1.57) | 1.08 (0.72-1.63) | 1.34 (0.96-1.89) | 1.02 (0.62-1.68) | 1.37 (0.95-1.97) | 0.79 (0.46-1.34) | 1.08 (0.73-1.59) |
|  | **sadness** | | | | | | | | | |
|  | Sex | Male | 1.00 (Reference) | 1.00 (Reference) | 1.00 (Reference) | 1.00 (Reference) | 1.00 (Reference) | 1.00 (Reference) | 1.00 (Reference) | 1.00 (Reference) |
|  |  | Female | **1.71 (1.63-1.79)** | **1.86 (1.58-2.20)** | **1.02 (0.75-1.37)** | **1.89 (1.47-2.42)** | 0.98 (0.68-1.4) | **1.85 (1.42-2.39)** | 0.78 (0.54-1.14) | **1.45 (1.10-1.90)** |
|  | Grade level | Middle school  (7-9^th^) | 1.00 (Reference) | 1.00 (Reference) | 1.00 (Reference) | 1.00 (Reference) | 1.00 (Reference) | 1.00 (Reference) | 1.00 (Reference) | 1.00 (Reference) |
|  |  | High school  (10-12^th^) | 1.03 (0.98-1.07) | 0.91 (0.77-1.09) | 1.25 (0.92-1.71) | 1.14 (0.88-1.48) | 0.77 (0.53-1.13) | 0.88 (0.67-1.16) | 1.05 (0.70-1.55) | 0.92 (0.69-1.22) |
|  | Residential area | Rural | 1.00 (Reference) | 1.00 (Reference) | 1.00 (Reference) | 1.00 (Reference) | 1.00 (Reference) | 1.00 (Reference) | 1.00 (Reference) | 1.00 (Reference) |
|  |  | Urban | **1.07 (1.02-1.12)** | 0.98 (0.83-1.15) | 1.00 (0.74-1.34) | 0.98 (0.77-1.26) | 0.89 (0.62-1.28) | 0.87 (0.66-1.14) | 1.18 (0.80-1.75) | 1.03 (0.78-1.36) |
|  | Recent alcohol consumption | No | 1.00 (Reference) | 1.00 (Reference) | 1.00 (Reference) | 1.00 (Reference) | 1.00 (Reference) | 1.00 (Reference) | 1.00 (Reference) | 1.00 (Reference) |
|  |  | Yes | **1.78 (1.70-1.87)** | **1.83 (1.52-2.20)** | 0.96 (0.67-1.39) | **1.76 (1.28-2.41)** | 0.92 (0.59-1.43) | **1.62 (1.19-2.21)** | 1.24 (0.78-1.97) | **2.01 (1.43-2.83)** |
|  | Smoking status | No | 1.00 (Reference) | 1.00 (Reference) | 1.00 (Reference) | 1.00 (Reference) | 1.00 (Reference) | 1.00 (Reference) | 1.00 (Reference) | 1.00 (Reference) |
|  |  | Yes | **1.77 (1.68-1.87)** | **1.64 (1.30-2.07)** | 1.23 (0.69-2.19) | **2.01 (1.18-3.41)** | 1.32 (0.63-2.77) | **2.65 (1.57-4.46)** | 0.96 (0.48-1.91) | **2.53 (1.61-3.98)** |
|  | Parental educational attainment | High school diploma or less | 1.00 (Reference) | 1.00 (Reference) | 1.00 (Reference) | 1.00 (Reference) | 1.00 (Reference) | 1.00 (Reference) | 1.00 (Reference) | 1.00 (Reference) |
|  |  | Bachelor's degree or higher | **1.10 (1.05-1.16)** | 1.11 (0.92-1.34) | 1.00 (0.72-1.39) | 1.11 (0.84-1.45) | 1.37 (0.92-2.04) | **1.52 (1.14-2.03)** | 0.78 (0.51-1.2) | 1.19 (0.87-1.63) |
|  | Academic achievement | Low | **0.92 (0.85-0.99)** | 0.94 (0.71-1.24) | 0.78 (0.47-1.30) | 0.73 (0.47-1.11) | 1.56 (0.84-2.90) | 1.14 (0.73-1.78) | 1.16 (0.61-2.20) | 1.32 (0.83-2.09) |
|  |  | Middle | 1.00 (Reference) | 1.00 (Reference) | 1.00 (Reference) | 1.00 (Reference) | 1.00 (Reference) | 1.00 (Reference) | 1.00 (Reference) | 1.00 (Reference) |
|  |  | High | **1.13 (1.07-1.20)** | 1.15 (0.91-1.45) | 1.03 (0.67-1.57) | 1.18 (0.83-1.68) | 0.83 (0.50-1.38) | 0.98 (0.68-1.40) | 1.22 (0.72-2.08) | 1.20 (0.81-1.77) |
|  | **Suicidal ideation** | | | | | | | | | |
|  | Sex | Male | 1.00 (Reference) | 1.00 (Reference) | 1.00 (Reference) | 1.00 (Reference) | 1.00 (Reference) | 1.00 (Reference) | 1.00 (Reference) | 1.00 (Reference) |
|  |  | Female | **1.81 (1.73-1.89)** | **1.75 (1.45-2.10)** | 1.18 (0.83-1.66) | **2.06 (1.54-2.75)** | 1.02 (0.69-1.52) | **2.11 (1.61-2.76)** | 1.01 (0.68-1.52) | **2.14 (1.58-2.89)** |
|  | Grade level | Middle school  (7-9^th^) | 1.00 (Reference) | 1.00 (Reference) | 1.00 (Reference) | 1.00 (Reference) | 1.00 (Reference) | 1.00 (Reference) | 1.00 (Reference) | 1.00 (Reference) |
|  |  | High school  (10-12^th^) | **0.81 (0.78-0.85)** | **0.66 (0.54-0.80)** | 1.26 (0.88-1.81) | 0.83 (0.61-1.12) | 0.84 (0.55-1.29) | **0.70 (0.52-0.94)** | 0.99 (0.65-1.50) | **0.69 (0.51-0.93)** |
|  | Residential area | Rural | 1.00 (Reference) | 1.00 (Reference) | 1.00 (Reference) | 1.00 (Reference) | 1.00 (Reference) | 1.00 (Reference) | 1.00 (Reference) | 1.00 (Reference) |
|  |  | Urban | 1.05 (1.00-1.10) | 0.97 (0.80-1.17) | 1.37 (0.97-1.95) | 1.33 (0.99-1.78) | 0.82 (0.55-1.23) | 1.09 (0.82-1.44) | 1.03 (0.68-1.55) | 1.12 (0.83-1.51) |
|  | Recent alcohol consumption | No | 1.00 (Reference) | 1.00 (Reference) | 1.00 (Reference) | 1.00 (Reference) | 1.00 (Reference) | 1.00 (Reference) | 1.00 (Reference) | 1.00 (Reference) |
|  |  | Yes | **1.75 (1.67-1.84)** | **1.73 (1.42-2.11)** | 1.14 (0.77-1.69) | **1.97 (1.40-2.77)** | 1.32 (0.82-2.12) | **2.59 (1.86-3.62)** | 0.67 (0.42-1.08) | **1.74 (1.24-2.44)** |
|  | Smoking status | No | 1.00 (Reference) | 1.00 (Reference) | 1.00 (Reference) | 1.00 (Reference) | 1.00 (Reference) | 1.00 (Reference) | 1.00 (Reference) | 1.00 (Reference) |
|  |  | Yes | **1.76 (1.67-1.86)** | **1.92 (1.53-2.41)** | 1.51 (0.85-2.67) | **2.89 (1.71-4.88)** | 0.90 (0.44-1.87) | **2.61 (1.58-4.32)** | 0.87 (0.45-1.70) | **2.28 (1.48-3.51)** |
|  | Parental educational attainment | High school diploma or less | 1.00 (Reference) | 1.00 (Reference) | 1.00 (Reference) | 1.00 (Reference) | 1.00 (Reference) | 1.00 (Reference) | 1.00 (Reference) | 1.00 (Reference) |
|  |  | Bachelor's degree or higher | **1.09 (1.04-1.15)** | 1.14 (0.94-1.39) | 0.94 (0.65-1.36) | 1.07 (0.78-1.46) | 1.51 (0.96-2.37) | **1.61 (1.16-2.23)** | 0.71 (0.45-1.15) | 1.15 (0.82-1.62) |
|  | Academic achievement | Low | 0.95 (0.88-1.03) | 1.11 (0.81-1.54) | 1.12 (0.61-2.06) | 1.24 (0.74-2.09) | 1.26 (0.62-2.54) | 1.56 (0.97-2.49) | 0.55 (0.28-1.10) | 0.86 (0.52-1.43) |
|  |  | Middle | 1.00 (Reference) | 1.00 (Reference) | 1.00 (Reference) | 1.00 (Reference) | 1.00 (Reference) | 1.00 (Reference) | 1.00 (Reference) | 1.00 (Reference) |
|  |  | High | **1.16 (1.09-1.24)** | 1.29 (0.99-1.69) | 1.21 (0.73-2.01) | **1.56 (1.01-2.40)** | 0.68 (0.38-1.22) | 1.06 (0.71-1.58) | 0.79 (0.45-1.39) | 0.84 (0.57-1.26) |
|  | **Suicidal attempt** | | | | | | | | | |
|  | Sex | Male | 1.00 (Reference) | 1.00 (Reference) | 1.00 (Reference) | 1.00 (Reference) | 1.00 (Reference) | 1.00 (Reference) | 1.00 (Reference) | 1.00 (Reference) |
|  |  | Female | **1.59 (1.48-1.71)** | **1.48 (1.12-1.95)** | 1.67 (0.98-2.85) | **2.47 (1.56-3.90)** | 0.61 (0.32-1.17) | 1.50 (0.94-2.40) | 1.2 (0.62-2.31) | **1.80 (1.14-2.84)** |
|  | Grade level | Middle school  (7-9^th^) | 1.00 (Reference) | 1.00 (Reference) | 1.00 (Reference) | 1.00 (Reference) | 1.00 (Reference) | 1.00 (Reference) | 1.00 (Reference) | 1.00 (Reference) |
|  |  | High school  (10-12^th^) | **0.66 (0.62-0.71)** | **0.65 (0.49-0.87)** | 1.00 (0.55-1.81) | 0.65 (0.39-1.10) | 1.09 (0.56-2.14) | 0.71 (0.46-1.09) | 0.79 (0.43-1.45) | **0.56 (0.36-0.85)** |
|  | Residential area | Rural | 1.00 (Reference) | 1.00 (Reference) | 1.00 (Reference) | 1.00 (Reference) | 1.00 (Reference) | 1.00 (Reference) | 1.00 (Reference) | 1.00 (Reference) |
|  |  | Urban | 0.97 (0.90-1.05) | 0.84 (0.63-1.12) | 1.06 (0.59-1.92) | 0.89 (0.53-1.49) | 0.67 (0.34-1.32) | **0.60 (0.39-0.92)** | 1.72 (0.93-3.17) | 1.03 (0.66-1.59) |
|  | Recent alcohol consumption | No | 1.00 (Reference) | 1.00 (Reference) | 1.00 (Reference) | 1.00 (Reference) | 1.00 (Reference) | 1.00 (Reference) | 1.00 (Reference) | 1.00 (Reference) |
|  |  | Yes | **2.51 (2.33-2.69)** | **3.25 (2.46-4.30)** | 1.1 (0.62-1.96) | **3.57 (2.15-5.93)** | 1.31 (0.65-2.64) | **4.66 (2.86-7.59)** | 0.64 (0.33-1.25) | **2.99 (1.89-4.71)** |
|  | Smoking status | No | 1.00 (Reference) | 1.00 (Reference) | 1.00 (Reference) | 1.00 (Reference) | 1.00 (Reference) | 1.00 (Reference) | 1.00 (Reference) | 1.00 (Reference) |
|  |  | Yes | **2.98 (2.77-3.21)** | **3.54 (2.59-4.83)** | 1.06 (0.49-2.31) | **3.75 (1.83-7.67)** | 1.43 (0.56-3.62) | **5.35 (2.96-9.68)** | 0.99 (0.47-2.08) | **5.27 (3.35-8.30)** |
|  | Parental educational attainment | High school diploma or less | 1.00 (Reference) | 1.00 (Reference) | 1.00 (Reference) | 1.00 (Reference) | 1.00 (Reference) | 1.00 (Reference) | 1.00 (Reference) | 1.00 (Reference) |
|  |  | Bachelor's degree or higher | 0.95 (0.87-1.03) | 0.85 (0.63-1.15) | 0.64 (0.34-1.21) | 0.54 (0.31-0.96) | 2.04 (0.97-4.29) | 1.10 (0.68-1.79) | 0.74 (0.37-1.48) | 0.81 (0.49-1.34) |
|  | Academic achievement | Low | 1.03 (0.90-1.18) | 1.03 (0.64-1.66) | 1.75 (0.61-5.02) | 1.80 (0.70-4.59) | 0.87 (0.26-2.93) | 1.56 (0.72-3.38) | 0.43 (0.14-1.31) | 0.67 (0.30-1.51) |
|  |  | Middle | 1.00 (Reference) | 1.00 (Reference) | 1.00 (Reference) | 1.00 (Reference) | 1.00 (Reference) | 1.00 (Reference) | 1.00 (Reference) | 1.00 (Reference) |
|  |  | High | **1.52 (1.37-1.69)** | 0.98 (0.66-1.46) | 1.8 (0.77-4.19) | 1.76 (0.83-3.71) | 0.76 (0.27-2.17) | 1.34 (0.64-2.78) | 0.84 (0.32-2.22) | 1.13 (0.60-2.13) |

Abbreviations: CI, Confidence Interval; KYRBS, the Korea Youth Risk Behavior Survey; wOR, weighted odds ratio.
Numbers in bold indicate a significant difference (p< 0.05).
